# Activation of the Silent Secondary Metabolite Production by Introducing Neomycin-Resistance in a Marine-Derived *Penicillium purpurogenum* G59

**DOI:** 10.3390/md13042465

**Published:** 2015-04-22

**Authors:** Chang-Jing Wu, Le Yi, Cheng-Bin Cui, Chang-Wei Li, Nan Wang, Xiao Han

**Affiliations:** 1 State Key Laboratory of Toxicology and Medical Countermeasures, Beijing Institute of Pharmacology and Toxicology, Beijing 100850, China; E-Mails: wucj2009@163.com (C.-J.W.); yileamms@126.com (L.Y.); sdrlcw@126.com (C.-W.L.); ammswang@126.com (N.W.); nullah876@sina.com (X.H.); 2 Key Laboratory of Structure-Based Drug Design & Discovery of Ministry of Education, School of Traditional Chinese Materia Medica, Shenyang Pharmaceutical University, Shenyang 110016, China

**Keywords:** *Penicillium purpurogenum* G59, marine-derived fungus, neomycin resistance, DMSO, antitumor activity, fungal metabolite production

## Abstract

Introduction of neomycin-resistance into a marine-derived, wild-type *Penicillium purpurogenum* G59 resulted in activation of silent biosynthetic pathways for the secondary metabolite production. Upon treatment of G59 spores with neomycin and dimethyl sulfoxide (DMSO), a total of 56 mutants were obtained by single colony isolation. The acquired resistance of mutants to neomycin was testified by the resistance test. In contrast to the G59 strain, the EtOAc extracts of 28 mutants inhibited the human cancer K562 cells, indicating that the 28 mutants have acquired the capability to produce bioactive metabolites. HPLC-photodiode array detector (PDAD)-UV and HPLC-electron spray ionization (ESI)-MS analyses further indicated that diverse secondary metabolites have been newly produced in the bioactive mutant extracts. Followed isolation and characterization demonstrated that five bioactive secondary metabolites, curvularin (**1**), citrinin (**2**), penicitrinone A (**3**), *erythro*-23-*O*-methylneocyclocitrinol (**4**) and 22*E*-7α-methoxy-5α,6α-epoxyergosta-8(14),22-dien-3β-ol (**5**), were newly produced by a mutant, 4-30, compared to the G59 strain. All **1**–**5** were also not yet found in the secondary metabolites of other wild type *P. purpurogenum* strains. Compounds **1**–**5** inhibited human cancer K562, HL-60, HeLa and BGC-823 cells to varying extents. Both present bioassays and chemical investigations demonstrated that the introduction of neomycin-resistance into the marine-derived fungal G59 strain could activate silent secondary metabolite production. The present work not only extended the previous DMSO-mediated method for introducing drug-resistance in fungi both in DMSO concentrations and antibiotics, but also additionally exemplified effectiveness of this method for activating silent fungal secondary metabolites. This method could be applied to other fungal isolates to elicit their metabolic potentials to investigate secondary metabolites from silent biosynthetic pathways.

## 1. Introduction

Fungi from marine habitats have come to be a rich source of new compounds with biological and pharmaceutical properties. A number of structurally novel and bioactive compounds, including a lot of drug leads and other health care ingredients [1,2,3,4], have been increasingly discovered from marine fungi in recent years [1,2,3,4,5]. However, the metabolic potential of fungi has not yet been fully elicited because the biosynthetic pathways of their major secondary metabolites are silenced in laboratory culture conditions [6,7]. There are various strategies that have been developed for activating silent microbial secondary metabolites over last decade [8,9,10,11,12,13,14,15,16,17]. Some of them, such as the one strain-many compounds (OSMAC) [16], co-cultivation [11], and chemical epigenetics [8,17] strategies, have been widely applied by microbial chemists to investigate silent fungal secondary metabolites. The culture-based, simple procedures outlined by these strategies are suited to microbial chemists. The recently developed mutagenesis strategy [14], which also outlined a simple procedure, may also suit to microbial chemists for the discovery of new compounds from silent fungal biosynthetic pathways [14,18,19,20]. Ribosome engineering [21,22] was also able to activate silent biosynthetic pathways by introducing drug-resistant mutation in bacteria to discover new compounds [23,24]. This strategy has recently been extended to fungi [13,15]. We have previously developed two new methods for introducing drug-resistance in fungi to activate silent biosynthetic pathways—the dimethyl sulfoxide (DMSO)-mediated [13] and the ultrasound-mediated [15] methods—extending the ribosome engineering strategy from bacteria to fungi. Although gentamycin, an antibacterial aminoglycoside that attacks the ribosome, has been testified to be applicable for introducing drug-resistance to fungi by the DMSO-mediated method [13], it is still worthwhile to further test other aminoglycosides by the same method. Using this method only gentamicin has so far been tested for introducing drug-resistance into fungi, but other aminoglycosides have already been used in bacteria in ribosome engineering [21,22].

**Figure 1:**
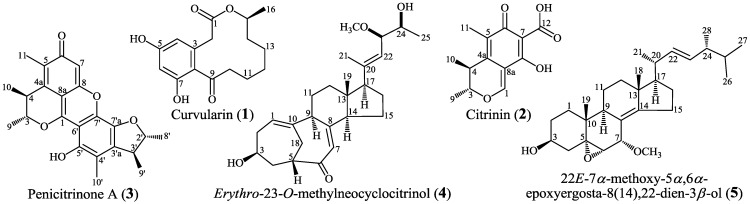
Structures of **1**–**5** newly produced in mutant 4-30 by activating silent metabolites in strain G59.

*Penicillium purpurogenum* G59, a marine-derived fungal strain initially isolated by our group [25], was originally inactive to produce secondary metabolites with antitumor activities in repeated MTT assays using human cancer K562 cells [13,14,18,19,20,25,26]. We have showed that the introduction of gentamicin-resistance into the G59 strain could result in the activated production of silent antitumor secondary metabolites in the G59 strain [13,26]. Generally, fungi are insensitive to antibacterial antibiotics, and the insensitivity of fungi to the antibacterial aminoglycoside antibiotics restricted their application to fungi in ribosome engineering. The low intracellular concentration of aminoglycosides restricted by fungal membrane permeability, coupled with their lower binding affinity to the eukaryotic rRNA than to the prokaryotic rRNA, have been known to be a major cause resulting in the insensitivity of fungi to aminoglycosides. Although the G59 strain was also insensitive to gentamicin, the acquired resistance of G59 strain to gentamicin could be introduced by the DMSO-mediated method, relying on the effect of DMSO on the penetration of antibiotics into cells [13]. As an extension of that work [13], we further tested neomycin in the present study to introduce drug-resistance in the G59 strain, instead of the previously used gentamicin [13], and DMSO was also used as a mediator for introducing drug-resistance. In the present study, the acquired resistance of mutants to neomycin was testified by the resistance tests, and the activated production of silent secondary metabolites in G59 strain by the introduction of neomycin-resistance in mutants was evidenced both by bioassays and HPLC-photodiode array detector (PDAD)-UV and HPLC-electron spray ionization (ESI)-MS analyses. Subsequent isolation and characterization further demonstrated that five bioactive secondary metabolites **1**–**5** (Figure 1) were newly produced in mutants 4-30. We report the results in detail in this paper.

## 2. Results and Discussion

### 2.1. Preliminary Test and Mutant Selection

**Figure 2:**
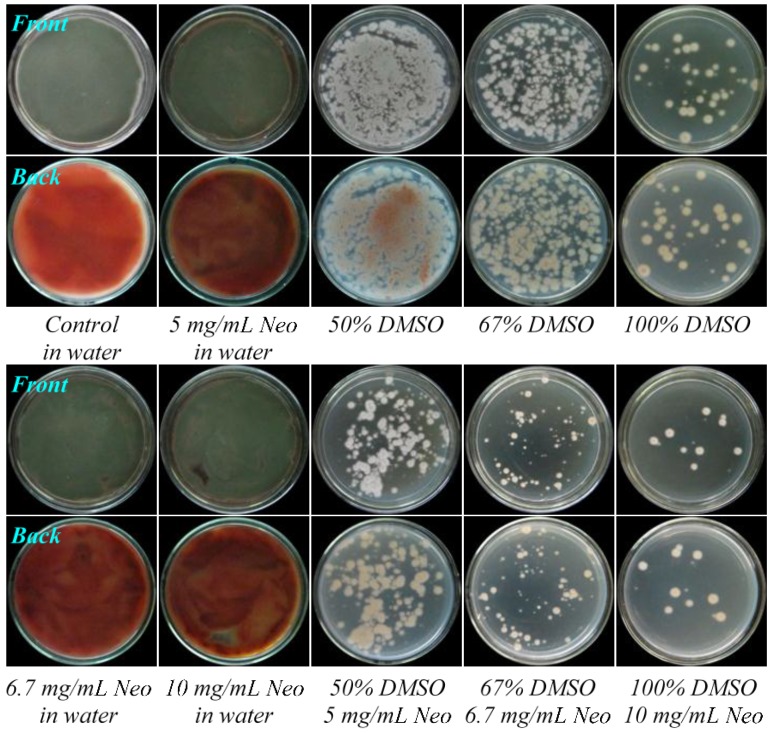
The growth of *P. purpurogenum* G59 on PDA plates by incubation at 28 °C for four days after pretreatment of G59 spores at 4 °C for two days. Descriptions under photographs indicate the spore pretreatment conditions. Neo is the abbreviation of neomycin.

According to our previous experience in the treatment of G59 spores by gentamicin in aqueous DMSO at 4 °C to introduce drug-resistance [13], we tested the introduction of neomycin-resistance in G59 strain by treatment of G59 spores with neomycin in the presence of DMSO at 4 °C for different times. The solubility of neomycin in 100% DMSO restricted its use by up to 10 mg/mL. We set five test groups I–V in Table 1 by combination of neomycin and DMSO, and preliminary tests were carried out for the III–V groups. In the preliminary test, we examined at first the effect of neomycin, DMSO and their combinations on the strain growth on potato dextrose agar (PDA) plates at 28 °C by treatment of G59 spores at 4 °C for two days. The G59 spores in water without neomycin and DMSO were also treated at 4 °C for 2 days as blank control. After treatment, each 50 μL of the treated spores was spread on PDA plates, incubated at 28 °C up to 6 days, and day-by-day growth of the G59 strain on the PDA plates was examined. The treatment of G59 spores with neomycin alone did not inhibit the strain growth on PDA plates at 28 °C, the strain grew as well as the control group (Figure 2), indicating the insensitivity of G59 strain to neomycin, as insensitive to gentamicin [13]. Although treatment of G59 spores with 50%, 67% or 100% DMSO inhibited to some extent the strain growth on PDA plates at 28 °C (Figure 2), the treatment of G59 spores with 5, 6.7 or 10 mg/mL neomycin in combination with 50%, 67% or 100% DMSO (Table 1) inhibited the strain growth more significantly, so as to allow development of resistant colonies on PDA plates at 28 °C (Figure 2), suitable for selecting drug-resistant mutants by single colony isolation.

**Table 1:** Group settings and conditions for treatment of G59 spores at 4 °C by neomycin in combination with dimethyl sulfoxide (DMSO) to select drug-resistant mutants ^a^.

Group	DMSO% (*v*/*v*)	Neomycin (mg/mL)	DMSO% Tested on G59 Strain	Treatment Times (Day)
I	20%	2.0	Previously in [13]	1–60
II	33%	3.3	Newly in present study	1–60
III	50%	5.0	Previously in [13]	1–60
IV	67%	6.7	Newly in present study	1–60
V	100%	10.0	Newly in present study	1–60

^a^ Fresh DMSO was used in all experiments and the used neomycin concentration was restricted up to 10 mg/mL by its solubility in 100% fresh DMSO. Spore suspensions were monitored by the optical density (OD) of 0.35 at 600 nm to keep the same spore density.

**Figure 3:**
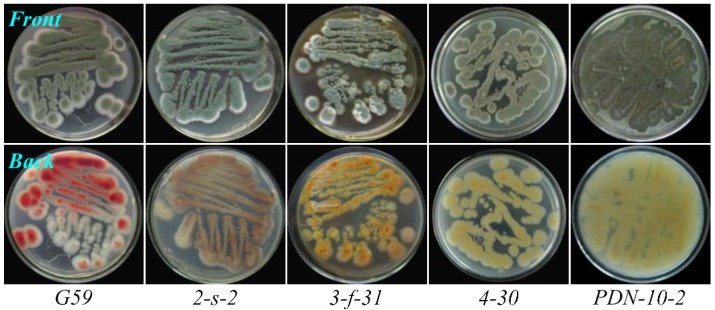
Phenotypes of the parent G59 strain and selected mutants growing on PDA plates by incubation at 28 °C for 4 days. Conditions for pretreatment of the G59 spores at 4 °C for getting the mutants are given as below. 2-s-2: 3.3 mg/mL Neo in 33% DMSO, 60 days; 3-f-31: 5.0 mg/mL Neo in 50% DMSO, 15 days; 4-30: 6.7 mg/mL Neo in 67% DMSO, seven days; PDN-10-2: 10.0 mg/mL Neo in 100% DMSO, one day.

On the basis of the preliminary test results, we carried out mutant selection by treatment of the G59 spores with neomycin in combination with DMSO at 4 °C for 1–60 days, as outlined in Table 1, and a total of 56 mutants were obtained by single colony isolation (Table 2). These mutants showed different phenotypes when growing on PDA plates at 28 °C, as shown examples in Figure 3. Appearances of the mutant colonies observed on PDA plates at 28 °C during mutant selection were not identical with their phenotypes, but once they were picked up, streaked out on fresh PDA plates, and then incubated at 28 °C, their own phenotypes appeared, as shown examples in Figure 3.

**Table 2:** Mutant numbers selected from parent G59 strain by treatment of the G59 spores with neomycin in combination with DMSO at 4 °C for different times ^a^.

Group	DMSO% (*v*/*v*)	Neomycin (mg/mL)	Treatment Time at 4 °C (Day)								Total
			1 Day	2 Day	5 Day	7 Day	10 Day	15 Day	30 Day	60 Day	
I	20%	2.0	NS	NS	NS	NS	NS	1	1	1	3
II	33%	3.3	NS	NS	NS	1	2	2	1	2	8
III	50%	5.0	NS	2	2	3	4	4	2	1	18
IV	67%	6.7	NS	1	3	4	4	2	NC	NC	14
V	100%	10.0	2	1	3	5	2	NC	NC	NC	13
Total			2	4	8	13	12	9	4	4	56

^a^ Fresh G59 spores were treated with neomycin at the given concentration in combination with the given concentration of DMSO at 4 °C for different times (day). Spore suspensions were monitored by their OD of 0.35 at 600 nm to keep the same spore density. Fresh DMSO was used for the spore treatment, and the spore suspensions at the same spore density in water without DMSO and neomycin and in DMSO without neomycin were used as controls, respectively. During the treatment period, each 100 μL portion of the treated spore suspensions was spread on PDA plates, incubated at 28 °C for 5–7 days, and mutant colonies developed on the PDA plates were selected by single colony isolation to obtain the aimed mutants. NS: No single colony could be selected due to the strain growth of test group throughout on the plate as the same as control group. NC: No colonies developed.

### 2.2. Resistance Test

Acquired resistance of mutants to neomycin was testified by the resistance test using four mutants, 3-f-31, 4-30, PDN-10-2 and PDN-v-2, and parent G59 strain. In the resistance test, fresh spores of each mutant and corresponding control G59 strain were treated as treated for selecting the mutant. Namely, the spores were treated with 5.0 mg/mL neomycin in 50% DMSO at 4 °C for 15 days for the mutant 3-f-31, treated with 6.7 mg/mL neomycin in 67% DMSO at 4 °C for seven days for the mutant 4-30, treated with 10.0 mg/mL neomycin in 100% DMSO at 4 °C for one day for the mutant PDN-10-2, and treated with 10.0 mg/mL neomycin in 100% DMSO at 4 °C for seven days for the mutant PDN-v-2. Each 50 μL of the treated spore suspensions was then spread on PDA plates, incubated at 28 °C for six days, and day-by-day growth of the mutants and G59 strain was examined.

**Figure 4:**
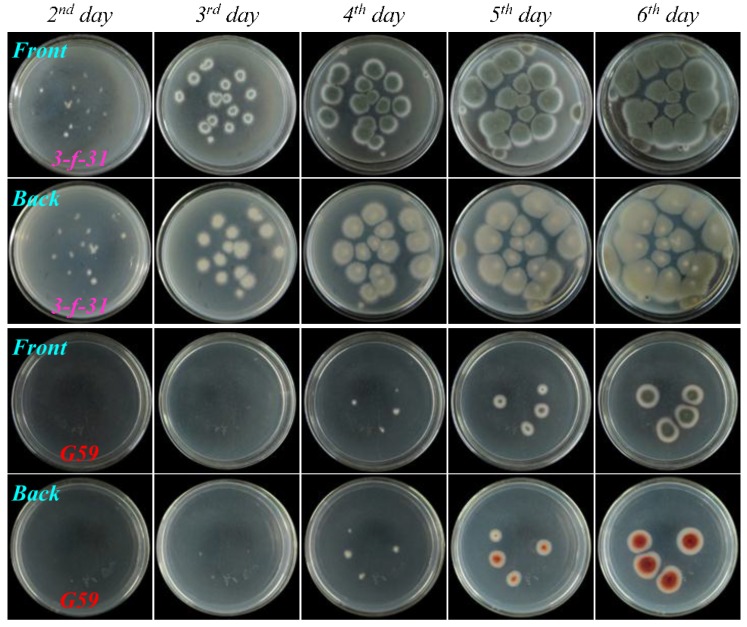
The growth of parent *P. purpurogenum* G59 strain and its mutant 3-f-31 on PDA plates by incubation at 28 °C for different times (day) after treatment of their spores with neomycin. Fresh G59 and 3-f-31 spores were treated with 5.0 mg/mL neomycin in 50% aqueous DMSO at 4 °C for 15 days, as done for selecting the mutant, and each 50 μL portion of the treated spore suspensions was spread on PDA plates, incubated at 28 °C, and photographed at the given incubation times (day).

**Figure 5:**
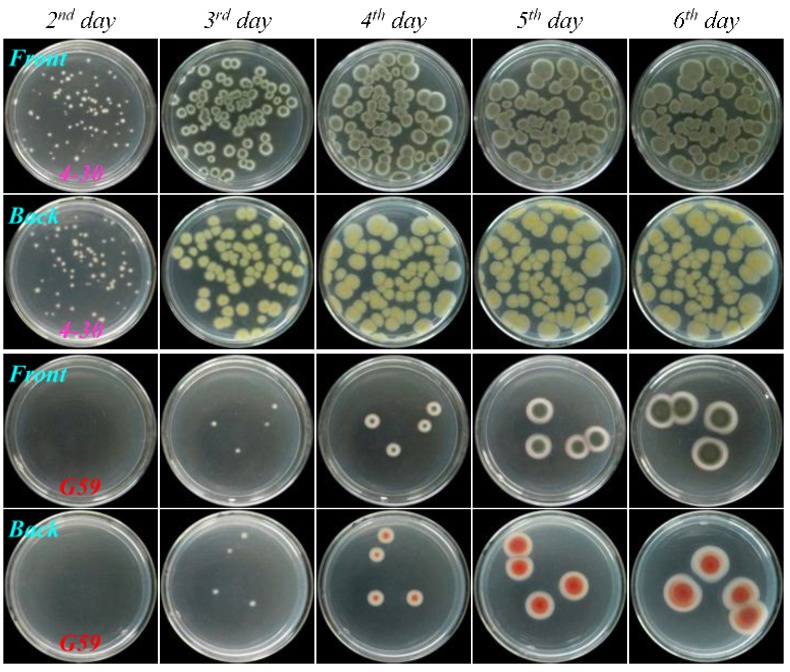
The growth of parent *P. purpurogenum* G59 strain and its mutant 4-30 on PDA plates by incubation at 28 °C for different times (day) after treatment of their spores with neomycin. Fresh G59 and 4-30 spores were treated with 6.7 mg/mL neomycin in 67% aqueous DMSO at 4 °C for seven days, as done for selecting the mutant, and each 50 μL of the treated spore suspensions was spread on PDA plates, incubated at 28 °C, and photographed at the given incubation times (day).

**Figure 6:**
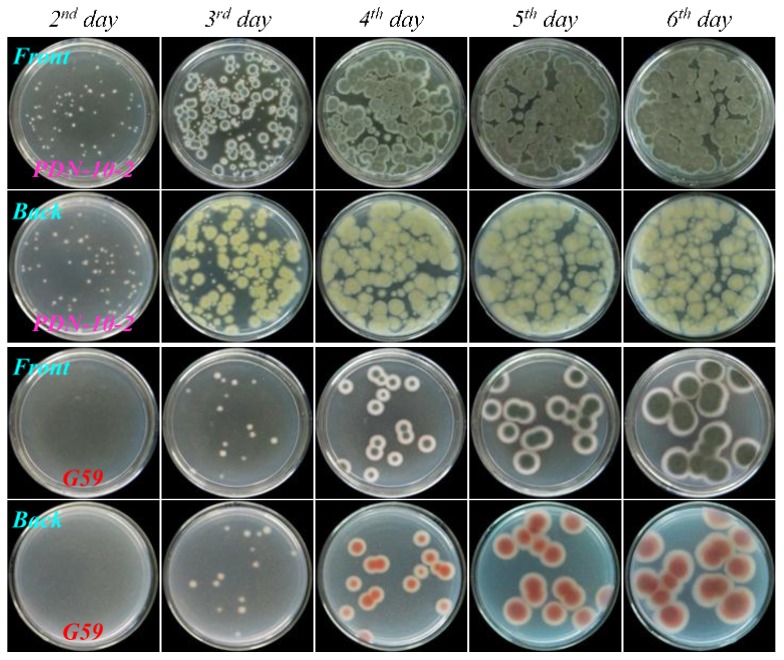
The growth of parent *P. purpurogenum* G59 strain and its mutant PDN-10-2 on PDA plates by incubation at 28 °C for different times (day) after treatment of their spores with neomycin. Fresh G59 and PDN-10-2 spores were treated with 10.0 mg/mL neomycin in 100% fresh DMSO at 4 °C for one day, as done for selecting the mutant, and each 50 μL portion of the treated spore suspensions was spread on PDA plates, incubated at 28 °C, and photographed at the given incubation times (day).

**Figure 7:**
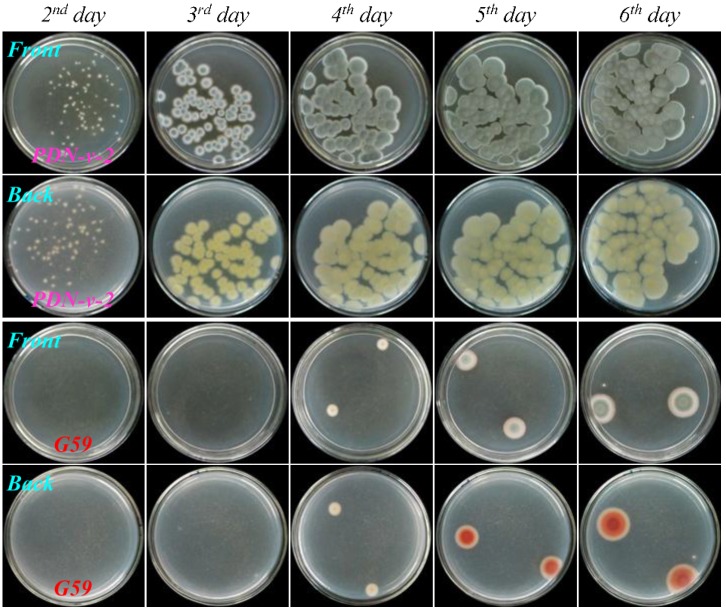
The growth of parent *P. purpurogenum* G59 strain and its mutant PDN-v-2 on PDA plates by incubation at 28 °C for different times (day) after treatment of their spores with neomycin. Fresh G59 and PDN-v-2 spores were treated with 10.0 mg/mL neomycin in 100% fresh DMSO at 4 °C for seven days, as done for selecting the mutant, and each 50 μL portion of the treated spore suspensions was spread on PDA plates, incubated at 28 °C, and photographed at the given incubation times (day).

As shown in Figure 4, Figure 5, Figure 6, and Figure 7, many colonies of the tested four mutants appeared early on the second day of incubation and quickly grew up into a confluent lawn, while several colony species of the G59 strain appeared later on the third day of incubation and grew up into isolated colonies. These results indicated acquired resistance of the mutants to neomycin by comparison with the parent G59 strain.

### 2.3. Estimation of Activated Bioactive Metabolite Production in Mutants by Bioassay

Activated production of bioactive metabolites in mutants was estimated by the MTT assay that has been repeatedly used in our previous studies [13,14,15,18,19,20,27] at first. The mutants and the control G59 strain were fermented at the same time with the same conditions to obtain their ethyl acetate (EtOAc) extracts. Then, the EtOAc extracts were subjected to the MTT assay on K562 cells to evaluate their antitumor activities. Extracts of 28 mutants, 50% of a total 56 mutants, inhibited the K562 cells with the inhibition rate (IR%) values at 100 μg/mL, shown in Table 3. In contrast, the EtOAc extract of the control G59 strain did not show inhibitory effect on K562 cells (an IR% value of 5.5% at 100 μg/mL), similar to our previous MTT assay results at 100 μg/mL [13,14,15,18,19,20,26] and 1000 μg/mL [13,25]. These bioassay data revealed that the 28 mutants produced antitumor metabolites but the control parent G59 strain did not, and thus indicated that the 28 mutants have acquired the metabolic capability to produce bioactive metabolites by the introduction of neomycin resistance.

**Table 3:** MTT assay results on K562 cells for the G59 strain and its mutant samples at 100 μg/mL ^a^.

Strain	Condition for Treatment of the G59 Spores at 4 °C to Select the Mutant			Inhibition Rate (IR%) at 100 μg/mL			
	DMSO% (*v*/*v*)	Neomycin (mg/mL)	Treatment Time (Day)	First	Second	Third	Mean ± SD
G59	–	–	–	4.8	3.9	7.9	5.5 ± 2.1
1-50-1	20	2	15	39.8	35.3	45.5	40.2 ± 5.1
2-2	33	3.3	10	27.7	47.6	30.9	35.4 ± 10.7
2-2-3	33	3.3	10	27.0	34.5	34.6	32.0 ± 4.4
2-50-1	33	3.3	15	32.6	31.4	19.8	27.9 ± 7.1
2-s-2	33	3.3	60	47.7	73.2	38.3	53.1 ± 18.1
2-s-3	33	3.3	60	46.0	59.2	36.0	47.1 ± 11.6
3-4-1	50	5.0	5	34.5	35.2	44.7	38.1 ± 5.7
3-4-2	50	5.0	5	30.8	31.5	41.8	34.7 ± 6.2
3-50-1	50	5.0	10	43.5	27.7	53.1	41.4 ± 12.8
3-50-2	50	5.0	10	69.2	31.7	40.6	47.2 ± 19.6
3-f-1	50	5.0	15	24.8	53.4	40.3	39.5 ± 14.3
3-f-3	50	5.0	15	31.6	33.8	31.5	35.2 ± 1.3
3-f-31	50	5.0	15	55.1	29.9	38.5	41.2 ± 12.8
3-v-1	50	5.0	30	30.4	35.3	44.9	36.9 ± 7.4
3-x-1	50	5.0	30	40.3	70.1	49.7	53.4 ± 15.2
3-s-1	50	5.0	60	24.0	24.7	34.5	27.7 ± 5.9
3-s-2	50	5.0	60	55.1	23.1	49.8	42.7 ± 17.2
4-4	67	6.7	5	42.5	34.3	30.7	35.8 ± 6.0
4-16-2	67	6.7	7	29.4	32.8	29.1	30.4 ± 2.1
4-30	67	6.7	7	50.4	53.1	36.1	46.5 ± 9.1
4-x-1	67	6.7	10	47.3	34.9	51.8	44.7 ± 8.8
4-v-2	67	6.7	10	34.9	38.6	38.9	37.5 ± 2.2
4-s-1	67	6.7	15	68.1	51.4	27.5	49.0 ± 20.4
PDN-10-2	100	10	1	41.3	52.4	23.8	39.2 ± 14.4
PDN-50-1	100	10	5	52.8	25.8	47.2	41.9 ± 14.2
PDN-v-1	100	10	7	29.3	22.9	26.0	26.1 ± 3.2
PDN-v-2	100	10	7	51.2	21.8	36.1	36.4 ± 14.7
PDN-s-2	100	10	10	38.8	35.4	32.2	35.5 ± 3.3

^a^ The IR% values given in this table were from the triplicate MTT tests that were carried out using the EtOAc extracts from three rounds of individual fermentations of the parent G59 strain and the 28 mutants, respectively. The K562 cells were treated with the sample at 37 °C for 24 h and then the IR% was measured by the MTT method.

### 2.4. Chromatographic Analysis of the Activated Secondary Metabolite Production in the Mutants

**Figure 8:**
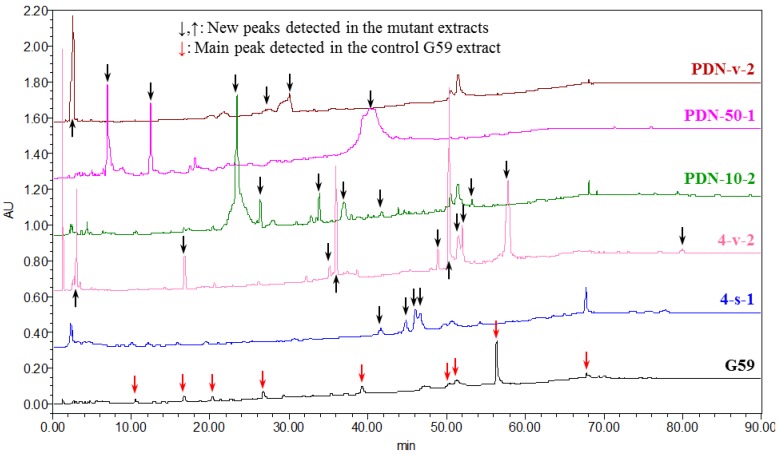
HPLC profiles of the EtOAc extracts of parent G59 strain and selected mutants, detected at 210 nm. The new peaks detected in mutant extracts, indicated by black arrows, were all verified by comparison of their UV spectra with those of G59 extract, respectively.

Activated secondary metabolite production in mutants by the introduction of neomycin resistance was examined by HPLC-PDAD-UV and HPLC-ESI-MS analyses. The EtOAc extracts of the control G59 strain and the 28 bioactive mutants were subjected to HPLC-PDAD-UV analysis, and the EtOAc extracts of four bioactive mutants, 2-50-1, 3-f-31, 4-30, and PDN-10-2, and control G59 strain were further subjected to HPLC-ESI-MS analysis. In the HPLC-PDAD-UV analysis, the G59 and 28 mutant extracts produced different HPLC profiles, and many new peaks were detected in the mutant extracts, as shown in Figure 8 and Supplementary Figure S1. The followed HPLC-ESI-MS analysis also evidenced the activated secondary metabolite production in the mutants compared to the parent G59 strain (Supplementary Figure S2). New peaks in the mutant extracts were verified by their UV (Supplementary Figure S1) and MS (Supplementary Figure S2) spectra and indicated that diverse secondary metabolites were being newly produced by these mutants. These analyses, together with the above mentioned bioassay results, indicated that some biosynthetic pathways originally silent in parent G59 strain were activated in these mutants to produce bioactive secondary metabolites. Further, it is worthy emphasizing that each bioactive mutant showed considerably different metabolite patterns, as seen in Figure 8 and Supplementary Figures S1 and S2. This revealed that these mutants seem to be applied to fully access the dormant secondary metabolites in the parent strain by investigating newly produced metabolites in the mutants. This thus also further indicated, coupled with our previous studies [13,15,26], that the method for introducing drug-resistance in fungi by the use of antibiotics and DMSO is likely to be a relatively simple, practical method for investigating the silent fungal secondary metabolites to search for new bioactive compounds.

### 2.5. Bioactive Metabolites **1**–**5** Newly Produced by Introducing Drug-Resistance in Mutant 4-30

#### 2.5.1. Fermentation, Isolation and Identification

Large-scale fermentation and extraction of the bioactive mutant 4-30 gave an EtOAc extract that inhibited K562 cells with an IR% of 51.2% at 100 μg/mL. However, the control G59 extract that was obtained by the fermentation of the G59 strain at the same time with the same conditions did not show any inhibitory effect on K562 cells (an IR% of 5.0% at 100 µg*/*mL). Repeated column chromatography of the mutant 4-30 extract, tracing newly produced bioactive metabolites by direct comparison with the control G59 extract, afforded **1**–**5** shown in Figure 1.

Compound **1**, ${\left[\text{α}\right]}_{\text{D}}^{20}$ −34.1 (*c* 0.7, EtOH), *m/z* 293 [M + H]^+^ in ESI-MS, was identified as curvularin [28] on the basis of its [α]_D_ and ^1^H and ^13^C NMR data. Compound **2**, ${\left[\text{α}\right]}_{\text{D}}^{20}$ −20.1 (*c* 0.5, EtOH), *m/z* 251 [M + H]^+^ in ESI-MS, and compound **3**, ${\left[\text{α}\right]}_{\text{D}}^{20}$ +81.3 (*c* 0.2, MeOH), *m/z* 381 [M + H]^+^ in ESI-MS, were identified as citrinin [13] and penicitrinone A [29] by their [α]_D_ and ^1^H and ^13^C NMR data, respectively. Compound **4**, ${\left[\text{α}\right]}_{\text{D}}^{20}$ +87.9 (*c* 1.2, MeOH), *m/z* 415 [M + H]^+^ in ESI-MS, and compound **5**, ${\left[\text{α}\right]}_{\text{D}}^{25}$ −53.6 (*c* 0.4, CHCl_3_), *m/z* 465 [M + Na]^+^ in ESI-MS, were identified as *erythro*-23-*O*-methyl neocyclocitrinol [19] and 22*E*-7α-methoxy-5α,6α-epoxyergosta-8(14),22-dien-3β-ol [30] according to their [α]_D_ and ^1^H and ^13^C NMR data, respectively.

#### 2.5.2. Inhibitory Effect of **1**–**5** on Several Human Cancer Cell Lines

The inhibitory effect of **1**–**5** was tested by the MTT assay on human cancer K562, HL-60, HeLa and BGC-823 cell lines. Compounds **1**–**5** inhibited the tested four human cancer cell lines by the inhibition rate (IR%) values ranging from 27.5% to 88.5% at the 100 μg/mL in Table 4, and the half inhibitory concentration (IC_50_) of **1**–**3** and **5** was determined as given in Table 5. The positive control docetaxol inhibited these cell lines with the IR% values of 79.9% (K562), 86.9% (HL-60), 78.6% (HeLa) and 61.5% (BGC-823) at 100 μg/mL.

**Table 4:** IR% values of **1***–***5** on human cancer cell lines at the 100 μg/mL ^a^.

Compound	K562	HL-60	HeLa	BGC-823
**1**	73.7%	70.0%	68.8%	66.0%
**2**	60.1%	79.4%	51.9%	67.1%
**3**	77.5%	82.0%	84.1%	79.9%
**4**	33.1%	43.3%	38.0%	27.5%
**5**	87.5%	86.5%	88.5%	86.4%

^a^ The cells were treated with the samples at 37 °C for 24 h and then the IR% was measured by the MTT method.

**Table 5:** IC_50_ values of **1**–**3** and **5** on human cancer cell lines in μg/mL (μM) ^a^.

Compound	K562	HL-60	HeLa	BGC-823
**1**	80.1 (274.3)	85.2 (291.8)	88.1 (301.7)	85.1 (291.4)
**2**	58.2 (232.8)	44.4 (177.6)	58.3 (233.2)	57.1 (228.4)
**3**	50.8 (133.7)	43.2 (113.7)	65.6 (172.6)	54.2 (142.6)
**5**	27.2 (61.5)	15.4 (34.8)	20.8 (47.1)	24.1 (54.5)

^a^ The cells were treated with the samples at 37 °C for 24 h and then the IC_50_ was measured by the MTT method.

#### 2.5.3. HPLC-PDAD-UV/HPLC-ESI-MS Analyses for Detecting **1**–**5** in the Mutant 4-30 Extract

The EtOAc extracts of mutant 4-30 and parent G59 strain were subjected to HPLC-PDAD-UV and HPLC-ESI-MS analyses to detect **1**–**5**. In the HPLC-PDAD-UV analysis, we could detect all **1**–**5** in the mutant 4-30 extract but not in the control G59 extract both by retention times and UV spectra using **1**–**5** as reference standards (Supplementary Figure S3). In parallel, **1**–**5** were also all detected in the mutant 4-30 extract by selective *pseudo*-molecular ion monitoring with both extracted ion chromatograms and related MS spectra, but none of these metabolites were detected in the G59 extract in the HPLC-ESI-MS analysis (Supplementary Figure S4). These analyses indicated that the production of **1**–**5** in the mutant 4-30 was caused by the activation of silent metabolic pathways in the parent G59 strain by the introduction of neomycin resistance in the mutant. Roughly, **1**–**3** and **4**–**5** belong to two different classes of natural products; polyketides and sterols, respectively. Therefore, at least the polyketide biosynthetic pathways including three subsets for **1**–**3** production and the sterol biosynthetic pathways including two subsets for **4**–**5** production should be activated in mutant 4-30 although details of the affected pathways remain unknown.

### 2.6. Discussion

In the present study, the acquired resistance of marine-derived *P. purpurogenum* G59 to neomycin was introduced by the treatment of G59 spores with neomycin in combination with DMSO, resulting in the activation of silent biosynthetic pathways for the secondary metabolite production in G59 strain. The same method using drug and DMSO was originally developed by our group as a new approach for introducing drug-resistance in fungi to activate silent secondary metabolites [13]. Previously [13], we tested and used the DMSO concentration up to 50% for introducing gentamicin-resistance into the G59 strain relying on the effect of DMSO on the penetration of drug into cells. In a continuation, we further extended the DMSO concentration up to 100% for introducing drug-resistance in the G59 strain using neomycin, instead of the previously used gentamicin [13], in the present study.

The treatment of G59 spores only with neomycin in the present study demonstrated the insensitivity of the G59 strain to neomycin (Figure 2), as previously done for gentamicin [13]. This indicated that the neomycin-resistance could not be introduced into the G59 strain by the treatment of the G59 spores with neomycin alone. Incidentally, we also tested to introduce neomycin-resistance using neomycin-containing PDA plates by the general method for introducing drug-resistance in bacteria in ribosome engineering [21,22]. In the test, the G59 strain grew well at 28 °C on the PDA plates containing 5, 6.7 and 10 mg/mL neomycin (data not shown), as the same as its growth on the PDA plates that did not contain neomycin. This thus also evidenced the insensitivity of the G59 strain to neomycin and further indicated that the drug-containing plate method routinely used for selecting bacterial resistant mutants in ribosome engineering [21,22] could not be used to the fungal strain for selecting neomycin-resistant mutants in the present study. In contrast, the treatment of G59 spores with 2.0–10.0 mg/mL neomycin in 20%–100% DMSO at 4 °C for different times (Table 1) could significantly inhibit the strain growth on PDA plates at 28 °C, allowing us to select drug-resistant mutants by single colony isolation (Table 2). The treatment of G59 spores with only 50%, 67% or 100% DMSO inhibited the strain growth on PDA plates at 28 °C to different extents. However, the treatment of G59 spores with 5.0, 6.7 or 10.0 mg/mL neomycin in combination with 50%, 67% or 100% DMSO could inhibit the strain growth more significantly (Figure 2), so as to allow development of drug-resistant colonies from which neomycin-resistant mutants can be selected by single colony isolation (Table 2). The spore suspension in 100% DMSO became solidified during saving at 4 °C for treatment. Even so, the treatment of G59 spores with neomycin in 100% DMSO showed stronger inhibition on strain growth on PDA plates at 28 °C than the treatment in 67% and 50% DMSO. During mutant selection, it was shown that the G59 strain did not grow at 28 °C on PDA plates (the complete inhibition of the strain growth) after treatment of the G59 spores by 10 mg/mL neomycin in 100% DMSO at 4 °C for 15 days and thereafter (Group V in Table 2). The similar phenomenon was also observed after treatment of the G59 spores with 6.7 mg/mL neomycin in 67% DMSO at 4 °C for 30 days and thereafter (Group IV in Table 2). In contrast, the treatment of G59 spores with neomycin for short times in Groups I–IV (the 1–10 days in Group I, the 1–3 days in Group II, and the 1 day in Groups III and IV in Table 2) did not significantly inhibit the strain growth, so as to allow the strain growth on PDA plates at 28 °C throughout the plate, as the same as the corresponding control group (Table 2). These observations indicated that the inhibition of the strain growth by treatment of G59 spores with neomycin in combination with DMSO at 4 °C was dependent on the concentrations of neomycin/DMSO and also on the treatment times. Upon treatment of the G59 spores with neomycin in combination with DMSO (Table 1), a total of 56 mutants were obtained by single colony isolation (Table 2), and acquired resistance of mutants to neomycin was testified by the resistance test as shown in Figure 4, Figure 5, Figure 6 and Figure 7. Thus, the use of DMSO was extended up to 100% DMSO for introducing neomycin resistance into the G59 strain in the present study.

Activated production of silent secondary metabolites in G59 strain by the introduction of neomycin-resistance in mutants was demonstrated both by bioassays and chemical analyses in the present study. This was further evidenced by followed elucidation of five bioactive secondary metabolites **1**–**5** from a mutant, 4-30, confirming that **1**–**5** were all newly produced by the mutant compared to the G59 strain. A literature survey showed that **1**–**5** were also not yet found in the secondary metabolites of other wild type *P. purpurogenum* strains. The biosynthesis of curvularins [31], including curvularin (**1**), and citrinin (**2**) [32] have been well investigated [31,32], and **3** was known as a citrinin derivative [29]. Compound **4** is one of the C25 steroids with an unusual bicycle [4.4.1]A/B ring system, and a plausible biosynthetic route has been proposed for the C25 steroids to explain the origin of their bicyclic system and side chain [33] from ergosterol for which regulation of its biosynthesis has been well explored [34]. Compound **5** is surely an ergosterol derivative [30] and the biosynthesis of **5** would be involved in the post-modification of ergosterol. Although the present study has proved that the production of **1**–**5** in mutant 4-30 was caused by the introduction of neomycin-resistance, activating metabolic pathways that were originally silent in the G59 strain, additional investigations are required for the interpretation of affected pathways and related mechanisms of activation in future. The introduction of certain mutations to the gene encoding for the ribosomal protein S12 (the *rpsL* mutations that confer bacterial resistance to aminoglycosides) or RNA polymerase β-subunit (the *rpoB* mutations that confer bacterial resistance to rifampicin) have been known to be able to regulate the bacterial secondary metabolisms by modulating related gene expression [21,22], and several new bioactive compounds had also been discovered from bacterial mutants by activating the silent pathways by introducing the *rpsL* and *rpoB* mutations [23,24]. The identification of mutated genes encoding ribosomal proteins, such as the *rpsL* gene, or the *in vitro* characterization of the mutant chromosomes in comparison with those from the parent wild-type strain, therefore, will probably be a first choice in investigations exploring biological mechanisms of the activated **1**–**5** production in mutant 4-30 in future studies.

Except for the mutant 4-30, several other bioactive mutants were also subjected to the investigation on the bioactive secondary metabolites newly produced in the mutants. These works and further work on other more bioactive secondary metabolites in the mutant 4-30 are in progress. In fact, more many bioactive secondary metabolites other than **1**–**5**, including some with novel structures that have been estimated by the literature surveys according to their planar and/or relative stereo structures, have been isolated from the mutants including the mutant 4-30, and we are now going forward with a focus on structural studies, especially the absolute configuration determinations for new compounds. These results will be published elsewhere in the near future. The present and ongoing work has indicated that the method for introducing drug-resistance in fungi, initially developed by our group using antibiotics and DMSO [13,26], could be applicable for activating silent pathways to explore dormant fungal secondary metabolites, including even the discovery of novel bioactive compounds.

## 3. Experimental Section

### 3.1. General Experimental

Melting points were measured on a Beijing Tiandiyu X-4 exact micro melting point apparatus (Tiandiyu science and technology Co., Ltd., Beijing, China) and the temperatures were not corrected. Optical rotations were measured on an Optical Activity Limited polAAr 3005 spectropolarimeter (Optical Activity Limited, Ramsey, United Kingdom). ESIMS was recorded on an Applied Biosystems API 3000 LC-MS spectrometer (AB SCIEX, Framingham, MA, USA). ^1^H and ^13^C NMR spectra were taken on a JEOL JNM-GX 400 (400 MHz ^1^H and 100 MHz ^13^C NMR) NMR spectrometer (JEOL Ltd., Tokyo, Japan), and chemical shifts were recorded in δ values using solvent signals (CDCl_3_: δ_H_ 7.26/δ_C_ 77.16; DMSO-*d*_6_: δ_H_ 2.50/δ_C_ 39.52; acetone-*d*_6_: δ_H_ 2.05/δ_C_ 206.26) as references, respectively.

Precoated silica gel GF_254_ plates (10 cm × 20 cm, 0.25 mm thickness for analytical TLC and 20 cm × 20 cm, 0.5 mm thickness for preparative TLC; Yantai Chemical Industrial Institute, Yantai, China) were used in TLC and spots were detected under sunlight and UV light (254 and 365 nm) or by using Vaughan’s reagent [13,19,20], 5% FeCl_3_ reagent [20] or 10% sulfuric acid reagent. Silica gel H (200–300 mesh, Yantai Chemical Industrial Institute, Yantai, China), YMC*GEL^®^ ODS-A-HG (12 nm S-50 μm, YMC Co., Ltd., Kyoto, Japan), and Sephadex™ LH-20 (GE Healthcare, Uppsala, Sweden) were used for column chromatography. Both analytical and preparative HPLC were performed on Waters HPLC systems equipped with Waters 600 controller, Waters 600 pump, Waters 2414 refractive index detector, Waters 2996 (for analytical HPLC) or 2998 (for preparative HPLC) photodiode array detector, and Waters Empower™ software (Waters, Milford, MA, USA). Venusil MP C_18_ (5 μm, 100 Å, 4.6 mm × 250 mm; Agela Technologies, Tianjin, China) and Capcell Pak C_18_ (MG II, 4.6 mm × 250 mm; Shiseido Co., Ltd., Tokyo, Japan) columns were used in analytical HPLC, and Capcell Pak C_18_ (MG II, 20 mm × 250 mm; Shiseido Co., Ltd., Tokyo, Japan) column was used in preparative HPLC.

ZHWY-2102 rotary shakers (Shanghai ZhiCheng Analyzing Instrument Manufactory Co., Ltd., Shanghai, China) were used for fermentation. A VERSAmax-BN03152 micro plate reader (Molecular Devices, Silicon Valley, CA, USA) was used to read the optical density (OD) and an AE31 EF-INV inverted microscope (Motic China Group Co., Ltd., Xiamen, Fujian, China) was used for examination of the tumor cell morphology.

Human chronic myelogenous leukemia K562 cell line was provided by Prof. Dr. Lili Wang (Beijing Institute of Pharmacology and Toxicology, Beijing, China). Human acute promyelocytic leukemia HL-60, human cervical cancer HeLa, and Human gastric adenocarcinoma BGC-823 cell lines were from Prof. Dr. Wenxia Zhou (Beijing Institute of Pharmacology and Toxicology). Fetal bovine serum was purchased from Tianjin Hao Yang Biological manufacture Co., Ltd. (Tianjin, China). The RPMI-1640 medium (lot No. 1403238) was purchased from Gibco (Grant Island, NY, USA) and MTT (lot No. 0793) from Amresco (Solon, OH, USA). Streptomycin (lot No. 071104) and penicillin (lot No. X11303302) were purchased from North China Pharmaceutical Group Corporation, Beijing, China. The docetaxol (DOC, lot No.20110326) was purchased from Aladdin Chemistry Co., Ltd. (Shanghai, China).

### 3.2. MTT Assay

EtOAc extracts and fractions were dissolved in MeOH at 10 mg/mL, and the MeOH solutions were used in MTT assays. Pure compounds and DOC were dissolved in MeOH to prepare 10.0 mg/mL stock solutions, respectively, and serial dilutions for the test compounds were made for MTT assay. DOC was used as positive control, and MeOH was used as blank control.

MTT assay was performed according to the procedure that we have repeatedly used in the previous studies [13,14,15,18,19,20,27]. Exponentially growing K562, HL-60, HeLa and BGC-823 cells were treated with samples at 37 °C for 24 h. The assay was run in triplicate, and the OD value was read at 570 nm on a VERSAmax-BN03152 plate reader. The IR% was calculated using OD mean values according to the formula, IR% = (OD_control_ − OD_sample_)/OD_control_ × 100%. The IC_50_ value for each compound was obtained from its IR% values at different concentrations.

### 3.3. Experiments for Introducing Neomycin Resistance into G59 Strain to Activate Silent Metabolites

#### 3.3.1. Initial Strain and Spore Suspension Preparation

*Penicillium purpurogenum* G59, used as an initial strain in the present study, was isolated from a soil sample collected at the tideland of Bohai Bay around Lüjühe in Tanggu District of Tianjin, China, in September 2004 [25]. This strain has been deposited at the China General Microbiological Culture Collection Center under the accession number CGMCC No.9721. This strain did not produce bioactive metabolites with antitumor activities in repeated MTT assays using K562 cells at 100 µg/mL [13,14,15,18,19,20,26] and 1000 µg/mL [13,25].

Fresh G59 spores were suspended in 80 mL sterilized-distilled water with several glass beads in a 100 mL Erlenmeyer flask and scattered well by shaken enough to prepare a crude spore suspension. A 100 μL portion of the suspension was added into a well of 96-well plates, diluted with water with its OD at 600 nm measured on a VERSAmax-BN03152 plate reader, and the dilution ratio was recorded when the OD reached 0.35. The whole crude spore suspension was then diluted with sterilized-distilled water in the same proportion to obtain a G59 spore suspension. This G59 suspension was used in the following experiments to maintain the same spore density in the all experiments concerned.

#### 3.3.2. Treatment of G59 Spores with Neomycin Coupled with DMSO and Mutant Selection

DMSO: fresh DMSO; H_2_O: sterilized-distilled water; ETs: sterilized 5 mL Eppendorf tubes. We prepared at first the neomycin (Neo) solutions in DMSO at 10.0 mg/mL (Neo-D) and in H_2_O at 10.0 mg/mL (Neo-H). Then, Neo-D, Neo-H, DMSO and H_2_O were mixed with the G59 spore suspension (G59-S) in ETs by the compositions given in Table 6, respectively, to prepare mixed spore suspensions for the test, Neo control, DMSO control and blank control groups in Groups I–IV (Table 6). Separately, approximately equal amounts of fresh G59 spores were suspended in each 3.0 mL of Neo-D, Neo-H, DMSO and H_2_O in ETs, keeping the spore density as same as possible, to prepare spore suspensions for the test, Neo control, DMSO control and blank control groups in Group V (Table 6).

**Table 6:** Composition of spore suspensions in Groups I–V for treatment of G59 spores at 4 °C ^a^.

Group	Concentration of DMSO and Neo in Test Group		Mixed Volume (mL) of Solution or Solvent with G59 Spore Suspension for I–IV Groups							
			Test Group		Neo Control		DMSO Control		Blank Control	
	DMSO%	Neo mg/mL	Neo-D	G59-S	Neo-H	G59-S	DMSO	G59-S	H_2_O	G59-S
I	20%	2.0	0.6	2.4	0.6	2.4	0.6	2.4	0.6	2.4
II	33%	3.3	1.0	1.0	1.0	1.0	1.0	1.0	1.0	1.0
III	50%	5.0	1.5	1.5	1.5	1.5	1.5	1.5	1.5	1.5
IV	67%	6.7	2.0	1.0	2.0	1.0	2.0	1.0	2.0	1.0
V	100%	10.0	Approx. eq. amt. of G59 spores in each 3.0 mL of Neo-D, Neo-H, DMSO and H_2_O.							

^a^ Neo is the abbreviation of neomycin. Neo-D: The neomycin solution in DMSO at 10.0 mg/mL; Neo-H: The neomycin solution in H_2_O at 10.0 mg/mL; G59-S: Fresh G59 spore suspensions in water (Section 3.3.1). H_2_O indicates sterilized-distilled water, and DMSO indicates fresh DMSO.

G59 spores in the above tubes were all treated at 4 °C for 1–60 days. During the treatment period, each 80 μL portion of the treated spore suspensions was spread on PDA plates at 1, 2, 5, 7, 10, 15, 30 and 60 days of the treatment and incubated at 28 °C for 5–7 days. Mutants from the test groups were obtained by single colony isolation, selecting colonies with different appearances.

#### 3.3.3. Resistance Test for Acquired Resistance of Four Mutants to Neomycin

The resistance test was performed on fresh spores using four mutants, 3-f-31, 4-30, PDN-10-2 and PDN-v-2, and the parent G59 strain as control. The spore suspensions of the four mutants and the G59 strain were prepared as described in Section 3.3.1 and Section 3.3.2. The spores of the mutants 3-f-31 and 4-30 were treated along with corresponding control G59 spores with 5.0 mg/mL neomycin in 50% DMSO at 4 °C for 15 days and 6.7 mg/mL neomycin in 67% DMSO at 4 °C for 7 days, respectively. The spores of the mutants PDN-10-2 and PDN-v-2 were treated along with corresponding control G59 spores with 10.0 mg/mL neomycin in 100% fresh DMSO at 4 °C for 1 and 7 days, respectively. Then, each 50 μL of the treated spore suspensions was spread on PDA plates, incubated at 28 °C for 6 days, and day-by-day growth of the mutants and G59 strain was examined.

#### 3.3.4. Fermentation and Preparation of EtOAc Extract for MTT Assay and Chemical Analysis

For the first round MTT test, the G59 strain and all 56 mutants were inoculated onto PDA plates from their PDA tube slants stocked at 4 °C and activated by incubation at 28 °C for 3–5 days. Then, approximately equal, suitable amounts of the activated, fresh G59 strain and 56 mutants growing on the PDA plates were inoculated using an inoculating loop into 200 mL of liquid medium (glucose 2%, maltose 1%, mannitol 2%, glutamic acid 1%, peptone 0.5%, and yeast extract 0.3% in distilled water) in a 500 mL Erlenmeyer flask and fermented at 28 °C for 12 days on a rotary shaker at 200 rpm. To each 200 mL of the whole fermentation broth was added 400 mL acetone, and then extracted by ultra-sonication for 2 h to give an aqueous acetone solution. The aqueous acetone solution was evaporated under reduced pressure to remove acetone. Then, the remaining water layer was extracted three times with the equal volumes of EtOAc to obtain an EtOAc solution. The evaporation of the EtOAc solution under reduced pressure followed by freeze-drying afforded EtOAc extracts.

An additional two rounds of individual fermentations and extractions were carried out for the control G59 strain and the 28 mutants that their EtOAc extracts showed antitumor activities in the first round MTT test. Fermentations and extractions were performed in the same manner at the same conditions as mentioned above. The EtOAc extracts from the two rounds of fermentations were all subjected to the MTT tests to confirm their activities. The EtOAc extracts from last round of fermentation was further used in the HPLC-PDAD-UV and HPLC-ESI-MS analyses.

#### 3.3.5. HPLC-PDAD-UV and HPLC-ESI-MS Analyses

The EtOAc extracts of 28 bioactive mutants and the G59 strain were subjected to HPLC-PDAD-UV analysis. The HPLC-PDAD-UV analysis was performed using an analytical Venusil MP C_18_ column (5 μm, 100 Å, 4.6 mm × 250 mm) on a Waters HPLC system equipped with Waters 600 controller, Waters 600 pump, Waters 2414 refractive index detector, Waters 2996 photodiode array detector and Waters Empower™ software. Each 10 μL of sample solutions in MeOH at 10.0 mg/mL was injected into the column and eluted with MeOH–H_2_O in a linear gradient (20% → 100% MeOH in 60 min followed by 30 min with isocratic 100% MeOH) as mobile phase (flow rate, 1 mL/min). The acquired photodiode array data were processed by the Empower software to obtain targeted HPLC-PDAD-UV data. Newly produced secondary metabolites in the mutant extracts were verified both by retention times (*t*_R_) and UV spectra compared to the control G59 extract.

The EtOAc extracts of the G59 strain and four bioactive mutants, 2-50-1, 3-f-31, 4-30, and PDN-10-2, were subjected to HPLC-ESI-MS analysis. The HPLC-ESI-MS analysis was performed on an LC-MS equipment equipped with an Agilent 1100 HPLC system, AB Sciex API 3000 LC-MS/MS system, and AB Sciex Analyst 1.4 software (AB SCIEX, Framingham, MA, USA). HPLC was carried out on the same Venusil MP C_18_ column (5 µm, 100 Å, 4.6 mm × 250 mm) at the same conditions of the HPLC-PDAD-UV analysis. The mass detector was set to scan a range from *m*/*z* 150–1500 in both positive and negative modes. The acquired data were processed by Analyst 1.4 software, and the newly produced secondary metabolites in mutant extracts were confirmed both by *t*_R_ values and MS spectra compared to the control G59 extract.

### 3.4. Experiments for Investigation on Compounds **1**–**5** from Mutant 4-30

#### 3.4.1. Large-Scale Fermentation and EtOAc Extract Preparation

The mutant 4-30 was inoculated into two Erlenmeyer flasks (500 mL) each containing 220 mL of liquid medium (glucose 2%, maltose 1%, mannitol 2%, glutamic acid 1%, peptone 0.5% and yeast extract 0.3% in distilled water, adjusted to pH 6.0 prior to sterilization) and cultured at 28 °C for 24 h on a rotary shaker at 200 rpm to obtain a seed culture (660 mL). Each 3 mL portion of the seed culture was inoculated into 116 of the 500 mL Erlenmeyer flasks each containing 220 mL of the same liquid medium. Then, the producing fermentation was performed on rotary shakers at 200 rpm at 28 °C for 12 days to obtain approximate 25 L of fermentation broth.

The whole broth (25 L) was filtrated to separate into filtrate and mycelium portions. The filtrate (20 L) was extracted three times with equal volumes of EtOAc (3 × 20 L) to obtain an EtOAc extract. The mycelium portion was extracted with 80% (*v*/*v*) aqueous acetone (4 × 5 L) by ultra-sonication for 3 h, followed by extraction at room temperature for 12 h, to give an aqueous acetone solution. The aqueous acetone solution was evaporated under reduced pressure and remaining water layer (4 L) was extracted three times with equal volumes of EtOAc (3 × 4 L) to obtain another EtOAc extract. Both the extracts from the filtrate and mycelia showed the same spots on TLC examinations and thus were combined to afford a total of 13.1 g of the EtOAc extract. The EtOAc extract inhibited K562 cells with an IR% of 51.2% at 100 µg*/*mL. This extract was used for investigation on **1**–**5** in the following experiments.

On the other hand, the parent G59 strain was also fermented in 220 mL of the same liquid medium in a 500 mL Erlenmeyer flask along with the mutant 4-30 fermentation. Extraction of the whole broth (220 mL) as described for the mutant 4-30 provided an EtOAc extract (89 mg), which did not show inhibitory effect on K562 cells (an IR% value of 5.0% at 100 µg*/*mL). This extract was used in the MTT assays and TLC analyses, and also in the HPLC-PDAD-UV and HPLC-ESI-MS analyses for detecting **1**–**5** in the mutant 4-30 extract, all as negative control.

#### 3.4.2. Isolation of Compounds **1**–**5**

The EtOAc extract (13 g) of the mutant 4-30 was subjected to silica gel column (silica gel, 150 g; bed, 6 cm × 22 cm) chromatography by stepwise elution with b.p. 60–90 °C petroleum ether (P)–dichloromethane (D)–methanol (M) (PD 1:0 → 0:1 and then DM 99:1 → 0:1) to obtain 11 fractions: **Fr-1** (3.5 g, eluted by P → PD 1:1), **Fr-2** (2.2 g, eluted by PD 1:1 → D), **Fr-3** (1.5 g, eluted by D → DM 99:1), **Fr-4** (2.2 g, eluted by DM 98:2 → 97:3), **Fr-5** (0.8 g, eluted by DM 97:3 → 96:4), **Fr-6** (0.5 g, eluted by DM 95:5 → 92:8), **Fr-7** (0.7 g, eluted by DM 92:8 → 90:10), **Fr-8** (0.5 g, eluted by DM 90:10 → 80:20), **Fr-9** (0.6 g, eluted by DM 80:20 → 70:30), **Fr-10** (0.6 g, eluted by DM 70:30 → 50:50), **Fr-11** (0.4 g, eluted by M). By TLC analysis, the spots corresponding to the newly produced **1**–**5** in the mutant extract were all detected in **Fr-4**, which inhibited K562 cells with an IR% of 49.7% at 100 μg/mL. Thus, **Fr-4** (2.2 g) was subjected to a Sephadex LH-20 column (bed, 5 cm × 40 cm in ethanol) and eluted with ethanol to afford six fractions in the order of elution: **Fr-4-1** (0.16 g), **Fr-4-2** (1.10 g), **Fr-4-3** (0.35 g), **Fr-4-4** (0.25 g), **Fr-4-5** (0.22 g) and **Fr-4-6** (0.10 g). Four of them, **Fr-4-2**–**Fr-4-4** and **Fr-4-6**, inhibited the K562 cells with the IR% of 67.0% (**Fr-4-2**), 54.0% (**Fr-4-3**), 55.5% (**Fr-4-4**) and 29.5% (**Fr-4-6**) at 100 μg/mL.

**Fr-4-2** (1.1 g) was further subjected to vacuum liquid chromatography on an ODS column (bed, 4 cm × 20 cm) dry-packed with 30 g of ODS. A stepwise elution with H_2_O (H)–MeOH (M) (100:0 → 0:100) gave five fractions: **Fr-4-2-1** (87 mg, eluted by HM 80:20 → 60:40), **Fr-4-2-2** (718 mg, eluted by HM 60:40 → 40:60), **Fr-4-2-3** (46 mg, eluted by HM 40:60), **Fr-4-2-4** (180 mg, eluted by HM 40:60 → 20:80), **Fr-4-2-5** (35 mg, eluted by HM 10:90) and **Fr-4-2-6** (28 mg, eluted by HM 10:90 → 0:100). Three fractions, **Fr-4-2-2**–**Fr-4-2-4**, inhibited the K562 cells with the IR% of 45.0% (**Fr-4-2-2**), 88.0% (**Fr-4-2-3**) and 81.2% (**Fr-4-2-4**) at 100 μg/mL. **Fr-4-2-2** was known to contain **4** by TLC examination, and total of this fraction (718 mg) was separated by preparative HPLC (column: Capcell Pak C_18_, MG II, 20 mm × 250 mm, room temperature; mobile phase: MeOH–H_2_O 70:30, flow rate: 8 mL/min; detecting wave length: 210 nm) to obtain **4** (15 mg, t*_R_* = 34.9 min). **Fr-4-2-3** (46 mg), known to contain **5** by TLC examination, was subjected to preparative HPLC as that for **4** except for the use of MeOH–H_2_O 95:5 as mobile phase to afford **5** (6 mg, *t*_R_ = 29.9 min). **Fr-4-2-4** also contained newly produced antitumor metabolites and these metabolites are being under separation.

**Fr-4-3** (0.35 g) was subjected to preparative HPLC as that for **4** except for the use of MeOH–H_2_O 72:28 as mobile phase to give **3** (19 mg, *t*_R_ = 35.3 min). **Fr-4-4** (0.25 g) was separated by preparative HPLC as that for **4** except for the use of MeOH–H_2_O 60:40 as mobile phase to obtain **1** (10 mg, *t*_R_ = 35.6 min). **Fr-4-6** (0.10 g) was subjected to preparative silica gel TLC developed by CHCl_3_–MeOH 95:5 and the silica gels on a band (R*_f_* ≈ 0.4) were harvested. Then, the substances adsorbed by the silica gels were eluted by CHCl_3_–MeOH 70:30 and recrystallized in MeOH to give **2** (20 mg).

#### 3.4.3. Physicochemical and Spectroscopic Data of **1**–**5**

The physicochemical and spectroscopic data of **1**–**4** are given in the Supplementary Information.

22*E*-7α-Methoxy-5α,6α-epoxyergosta-8(14),22-dien-3β-ol (**5**): A white amorphous powder (from MeOH), ${\left[\text{α}\right]}_{\text{D}}^{25}$ −53.6 (*c* 0.4, CHCl_3_). Positive ESI-MS *m/z*: 465 [M + Na]^+^, 907 [2M + Na]^+^. ^1^H NMR (400 MHz, CD_3_OD) δ: 5.26 (1H, dd, *J* = 15.2, 6.4 Hz, H-22), 5.22 (1H, dd, *J* = 15.2, 7.4 Hz, H-23), 4.20 (1H, d, *J* = 2.9 Hz, H-7), 3.75 (1H, tt, *J* = 11.3, 4.6 Hz, H-3), 3.43 (3H, s, CH_3_O-7), 3.25 (1H, d, *J* = 2.9 Hz, H-6), 2.53–2.41 (1H, m, Ha-15), 2.40–2.31 (1H, m, H-9), 2.29–2.18 (1H, m, Hb-15), 2.14 (1H, dd, *J* = 13.1, 11.7 Hz, Ha-4), 2.16–2.07 (1H, m, H-20), 2.00–1.82 (3H, m, Ha-2, Ha-12 and H-24), 1.77–1.64 (2H, m, Ha-1 and Ha-16), 1.60–1.16 (9H, m, Hb-1, Hb-2, Hb-4, H_2_-11, Ha-12, Hb-16, H-17 and H-25), 1.04 (3H, d, *J* = 6.7 Hz, H_3_-21), 0.95 (3H, d, *J* = 6.8 Hz, H_3_-28), 0.91 (3H, s, H_3_-18), 0.89 (3H, s, H_3_-19), 0.87 (3H, d, *J* = 6.8 Hz, H_3_-26), 0.85 (3H, d, *J* = 6.8 Hz, H_3_-27). ^13^C NMR (100 MHz, CD_3_OD) δ: 154.5 (C-14), 136.8 (C-22), 133.4 (C-23), 124.1 (C-8), 74.3 (C-7), 69.3 (C-3), 67.0 (C-5), 59.3 (C-6), 58.2 (C-17), 55.1 (CH_3_O-7), 44.4 (2C, C-13 and C-24), 41.5 (C-9), 40.7 (C-4), 40.4 (C-20), 37.9 (C-12), 37.1 (C-10), 34.4 (C-25), 33.4 (C-1), 31.9 (C-2), 28.4 (C-16), 25.8 (C-15), 21.8 (C-21), 20.5 (C-26), 20.2 (C-27), 20.1 (C-11), 18.6 (C-18), 18.2 (C-28), 16.9 (C-19). The ${\left[\text{α}\right]}_{\text{D}}^{25}$ and ^1^H NMR data of **5** in CDCl_3_ in the Supplementary Information are identical with those in the literature [30].

#### 3.4.4. Examination of **1**–**5** in EtOAc Extracts of Mutant 4-30 and Parent G59 Strain

Examination of **1**–**5** in the EtOAc extracts of mutant 4-30 and strain G59 was carried out by HPLC-PDAD-UV and HPLC-ESI-MS analyses. A sample solution of crude **1**–**5** in MeOH at 10 mg/mL was used as reference standard in the HPLC-PDAD-UV analysis. HPLC-PDAD-UV and HPLC-ESI-MS were performed at the conditions given in Section 3.3.5. In the HPLC-PDAD-UV analysis, **1**–**5** were elucidated as peaks with retention times (*t*_R_), 40.88 min for **1**, 19.02 min for **2**, 54.30 min for **3**, 51.65 min for **4**, and 69.53 min for **5**. Compounds **1**–**5** were all detected in the mutant 4-30 extract but not in the G59 extract both by retention times and UV spectra (Supplementary Figure S3). In the HPLC-ESI-MS analysis, retention times of the **1**–**5** ion peaks were slightly shortened than those in the HPLC-PDAD-UV analysis because of the shortened flow length from the outlet of HPLC column to the inlet of MS in the HPLC-ESI-MS. Their ion peaks appeared at the retention times, 37.0–38.5 min for **1**, 17.0–17.5 min for **2**, 50.0–51.5 min for **3**, 48.0–49.0 min for **4**, and 66.5–67.5 min for **5**. By selective ion ([M + H]^+^, [M − H]^−^ and/or [M + Na]^+^) monitoring with both the extracted ion chromatograms and related MS spectra, **1**–**5** were all detected in the mutant 4-30 extract, but none of these metabolites were detected in the G59 extract (Supplementary Figure S4).

## 4. Conclusions

The introduction of acquired resistance of *P. purpurogenum* G59 to neomycin could activate silent secondary metabolite production. The acquired resistance could be introduced by treatment of the G59 spores with neomycin and DMSO at 4 °C. The present work not only extended the previous DMSO-mediated method for introducing drug-resistance in fungi both in DMSO concentrations and antibiotics, but also additionally exemplified the effectiveness of this method for activating silent fungal secondary metabolites. This method could be applied to other fungal isolates to elicit their metabolic potentials to investigate bioactive secondary metabolites from silent biosynthetic pathways.

## Supplementary Files

Supplementary File 1
