# Supplementary material for: Activation of the Silent Secondary Metabolite Production by Introducing Neomycin-Resistance in a Marine-Derived Penicillium purpurogenum G59
Source: Mar Drugs. 2015 Apr 22;13(4):2465–87. doi: 10.3390/md13042465 (PMC4413221; doi:10.3390/md13042465)
Supplement: Supplementary File 1 [file marinedrugs-13-02465-s001.pdf]

# Supplementary Information

## S1. Physicochemical and spectroscopic data of 1–5

Curvularin (**1**): White tabular crystals (from MeOH), m.p. 199–200 ℃, $[\alpha]_D^{20}$ −34.1 (*c* 0.7, EtOH) and $[\alpha]_D^{20}$ −38.3 (*c* 1.2, MeOH). Positive ESI-MS *m/z*: 293 $[M + H]^+$, 315 $[M + Na]^+$; negative ESI-MS *m/z*: 291 $[M + Cl]^-$. $^1$H NMR (400 MHz, acetone-$d_6$) δ: 9.09 (1H, br s, HO-7), 8.76 (1H, br s, HO-5), 6.38 (1H, d, *J* = 2.3 Hz, H-6), 6.35 (1H, d, *J* = 2.3 Hz, H-4), 4.96–4.87 (1H, m, H-15), 3.78 (1H, d, *J* = 15.7 Hz, Ha-2), 3.70 (1H, d, *J* = 15.7 Hz, Hb-2), 3.11 (1H, ddd, *J* = 15.5, 8.4, 3.0 Hz, Ha-10), 2.77 (1H, ddd, *J* = 15.5, 9.7, 2.9 Hz, Hb-10), 1.80–1.20 (8H, m, $H_2$-11 → $H_2$-14), 1.11 (3H, d, *J* = 6.3 Hz, $H_3$-16). $^{13}$C NMR (400 MHz, acetone-$d_6$) δ: 206.7 (C-9), 171.0 (C-1), 160.1 (C-5), 158.2 (C-7), 136.9 (C-3), 121.3 (C-8), 112.2 (C-4), 102.5 (C-6), 72.5 (C-15), 43.9 (C-10), 39.7 (C-2), 32.8 (C-14), 27.5 (C-12), 24.5 (C-13), 23.4 (C-11), 20.6 (C-16).

Citrinin (**2**): Lemon yellow needles (MeOH), m.p. 179–180 ℃, $[\alpha]_D^{20}$ −20.1 (*c* 0.5, EtOH). Positive ESI-MS *m/z*: 251 $[M + H]^+$, 273 $[M + Na]^+$; negative ESI-MS *m/z*: 249 $[M - H]^-$. $^1$H NMR (400 MHz, $CDCl_3$) δ: 15.88 (1H, s, HO-12), 15.11 (1H, s, HO-8), 8.23 (1H, s, H-1), 4.77 (1H, q, *J* = 6.7 Hz, H-3), 2.98 (1H, q, *J* = 7.2 Hz, H-4), 2.02 (3H, s, $H_3$-11), 1.34 (3H, d, *J* = 6.7 Hz, $H_3$-9), 1.22 (3H, d, *J* = 7.2 Hz, $H_3$-10). $^{13}$C NMR (100 MHz, $CDCl_3$) δ: 184.0 (C-6), 177.3 (C-8), 174.7 (C-12), 162.9 (C-1), 139.2 (C-4a), 123.2 (C-5), 107.5 (C-8a), 100.5 (C-7), 81.8 (C-3), 34.7 (C-4), 9.6 (C-10), 18.4 (C-9), 18.7 (C-11).

Penicitrinone A (**3**): An orange crystalline powder (MeOH), m.p. 153–155 ℃, $[\alpha]_D^{20}$ +81.3 (*c* 0.2, MeOH). Positive ESI-MS *m/z*: 381 $[M + H]^+$; negative ESI-MS *m/z*: 379 $[M - H]^-$. $^1$H NMR (400 MHz, $CDCl_3$) δ: 8.34 (1H, br s, HO-5'), 6.38 (1H, s, H-7), 4.97 (1H, br q, *J* = 6.6 Hz, H-3), 4.61 (1H, qd, *J* = 6.7, 4.1 Hz, H-2'), 3.16 (1H, qd, *J* = 6.7, 4.1 Hz, H-3'), 3.12 (1H, br q, *J* = 7.0 Hz, H-4), 2.20 (3H, s, $H_3$-10'), 2.11 (3H, s, $H_3$-11), 1.43 (3H, d, *J* = 6.4 Hz, $H_3$-9), 1.42 (3H, d, *J* = 6.2 Hz, $H_3$-8'), 1.33 (3H, d, *J* = 6.9 Hz, $H_3$-9'), 1.31 (3H, d, *J* = 7.0 Hz, $H_3$-10). $^{13}$C NMR (100 MHz, $CDCl_3$) δ: 184.5 (C-6), 158.2 (C-8), 155.7 (C-1), 147.6 (C-5'), 139.5 (C-3'a), 138.0 (C-7'a), 135.9 (C-7'), 132.0 (C-5), 131.0 (C-4a), 116.6 (C-4'), 103.4 (C-7), 102.4 (C-6'), 100.2 (C-8a), 88.1 (C-2'), 82.5 (C-3), 44.8 (C-3'), 35.1 (C-4), 21.1 (C-9), 19.2 (C-9'), 19.1 (C-10), 19.0 (C-8'), 11.6 (C-10'), 10.9 (C-11).

*Erythro*-23-*O*-methylneocyclocitrinol (**4**): yellowish solid (MeOH), $[\alpha]_D^{20}$ +87.9 (*c* 1.2, MeOH). Positive ESIMS *m/z*: 415 $[M + H]^+$, 437 $[M + Na]^+$; negative ESI-MS *m/z*: 413 $[M - H]^-$. $^1$H NMR (400 MHz, DMSO-$d_6$) δ: 5.56 (1H, dd, *J* = 8.3, 6.3 Hz, H-1), 5.43 (1H, s, H-7), 5.12 (1H, d, *J* = 9.0 Hz, H-22), 4.63 (1H, d, *J* = 4.3 Hz, HO-3), 4.44 (1H, d, *J* = 4.9 Hz, HO-24), 3.79/3.77(1H, dd, *J* = 8.9, 4.0 Hz, H-23), 3.65–3.56 (1H, m, H-24), 3.16/3.15 (3H, s, $CH_3O$-23), 3.15–3.07 (1H, m, H-3), 2.85 (1H, dd, *J* = 11.6, 5.5 Hz, H-9), 2.71–2.65 (1H, m, H-5), 2.63 (1H, br d, *J* = 13.0, Hα-4), 2.48 (2H, br s, $H_2$-18), 2.39–2.27 (2H, m, Hβ-2 and H-17), 2.26 (1H, br t, *J* = 9.0 Hz, H-14), 2.11–2.01 (1H, m, Hα-2), 1.90–1.62 (4H, m, Hβ-11, Hβ-12 and $H_2$-16), 1.68 (3H, s, $H_3$-21), 1.62–1.42 (5H, m, Hβ-4, Hα-11, Hα-12 and $H_2$-15), 0.99/0.97 (3H, d, *J* = 6.3 Hz, $H_3$-25), 0.54/0.53 (3H, s, $H_3$-19). $^{13}$C NMR (100 MHz, DMSO-$d_6$) δ: 204.2 (C-6), 156.96/156.98 (C-8), 145.6 (C-10), 138.71/138.82 (C-20), 124.4/124.3 (C-22), 124.3 (C-7), 122.1 (C-1), 81.3/81.4 (C-23), 68.32/68.38 (C-24), 63.1 (C-3), 58.87/58.92 (C-17), 55.5 ($CH_3O$-23), 54.4 (C-14), 53.3 (C-9), 48.1 (C-5), 46.78/46.80 (C-13), 41.4 (C-4), 37.08/37.13 (C-12), 36.0 (C-2), 27.5 (C-11), 27.2 (C-18), 23.8 (C-16), 22.36/22.40 (C-15), 19.0/18.8 (C-25), 18.29/18.07 (C-21), 13.47/13.51 (C-19).

22*E*-7α-Methoxy-5α,6α-epoxyergosta-8(14),22-dien-3β-ol (**5**): $[\alpha]_D^{25}$ −53.6 (*c* 0.4, $CHCl_3$). Positive ESI-MS *m/z*: 465 $[M + Na]^+$. $^1$H NMR (400 MHz, $CDCl_3$) δ: 5.22 (1H, dd, *J* = 15.2, 6.8 Hz, H-22), 5.17 (1H, dd, *J* = 15.2, 7.5 Hz, H-23), 4.16 (1H, d, *J* = 3.0 Hz, H-7), 3.93 (1H, tt, *J* = 11.6, 4.6 Hz, H-3), 3.41 (3H, s, $CH_3O$-7), 3.20 (1H, d, *J* = 3.0 Hz, H-6), 2.57–2.45 (1H, m, Ha-15), 2.43–2.36 (1H, m, H-9), 2.25–2.15 (1H, m, Hb-15), 2.14 (1H, dd, *J* = 13.0, 11.6 Hz, Ha-4), 2.13–2.07 (1H, m, H-20), 2.00–1.80 (3H, m, Ha-2, Ha-12, H-24), 1.75–1.60 (2H, m, Ha-1, Ha-16), 1.59–1.20 (9H, m, Hb-1, Hb-2, Hb-4, $H_2$-11, Ha-12, Hb-16, H-17, H-25), 1.01 (3H, d, *J* = 6.7 Hz, $H_3$-21), 0.91 (3H, d, *J* = 6.8 Hz, $H_3$-28), 0.86 (6H, s, $H_3$-18, $H_3$-19), 0.83 (3H, d, *J* = 6.6 Hz, $H_3$-26), 0.82 (3H, d, *J* = 6.6 Hz, $H_3$-27).

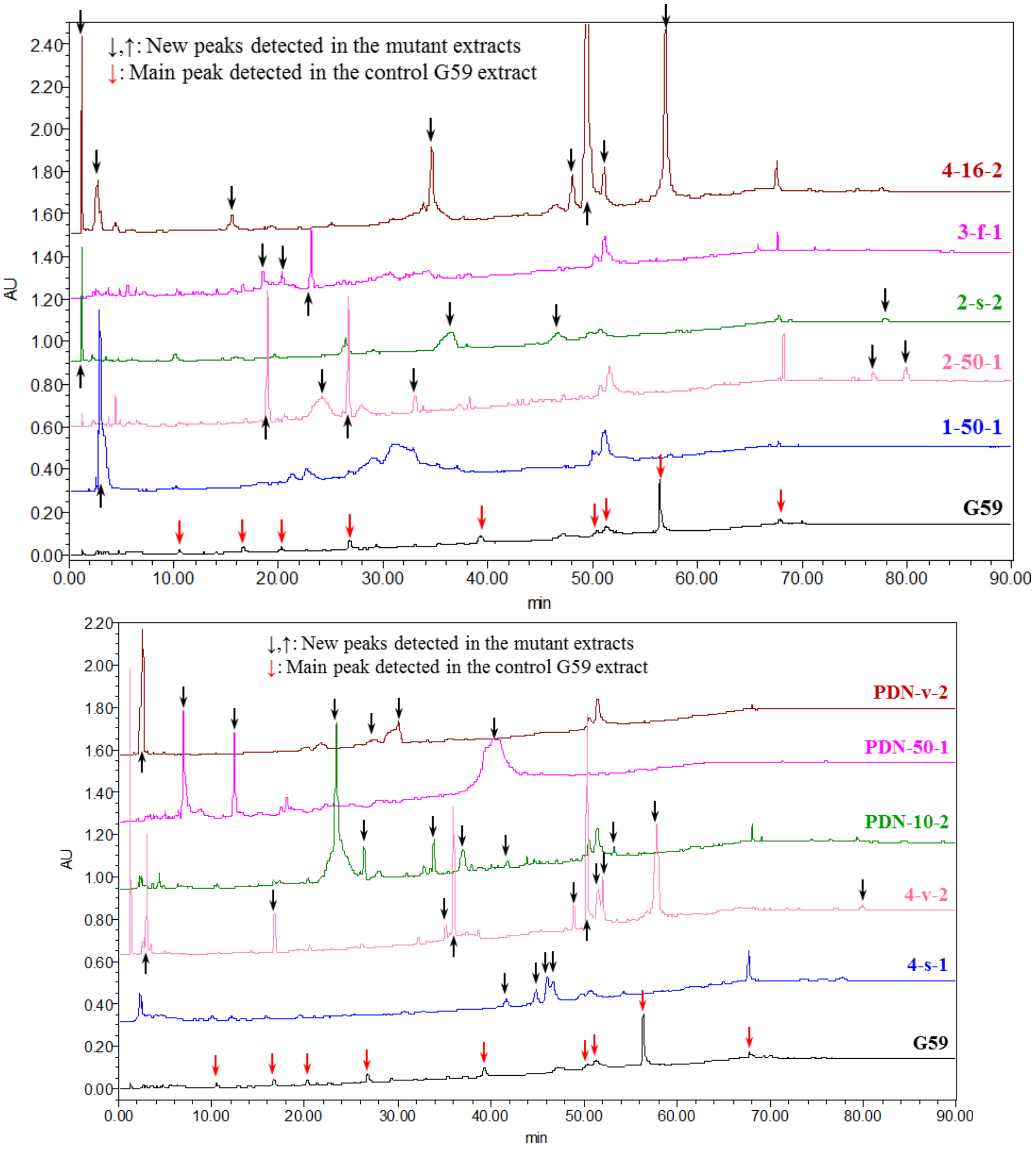


**(A1)**

**Figure S1.** *Cont.*

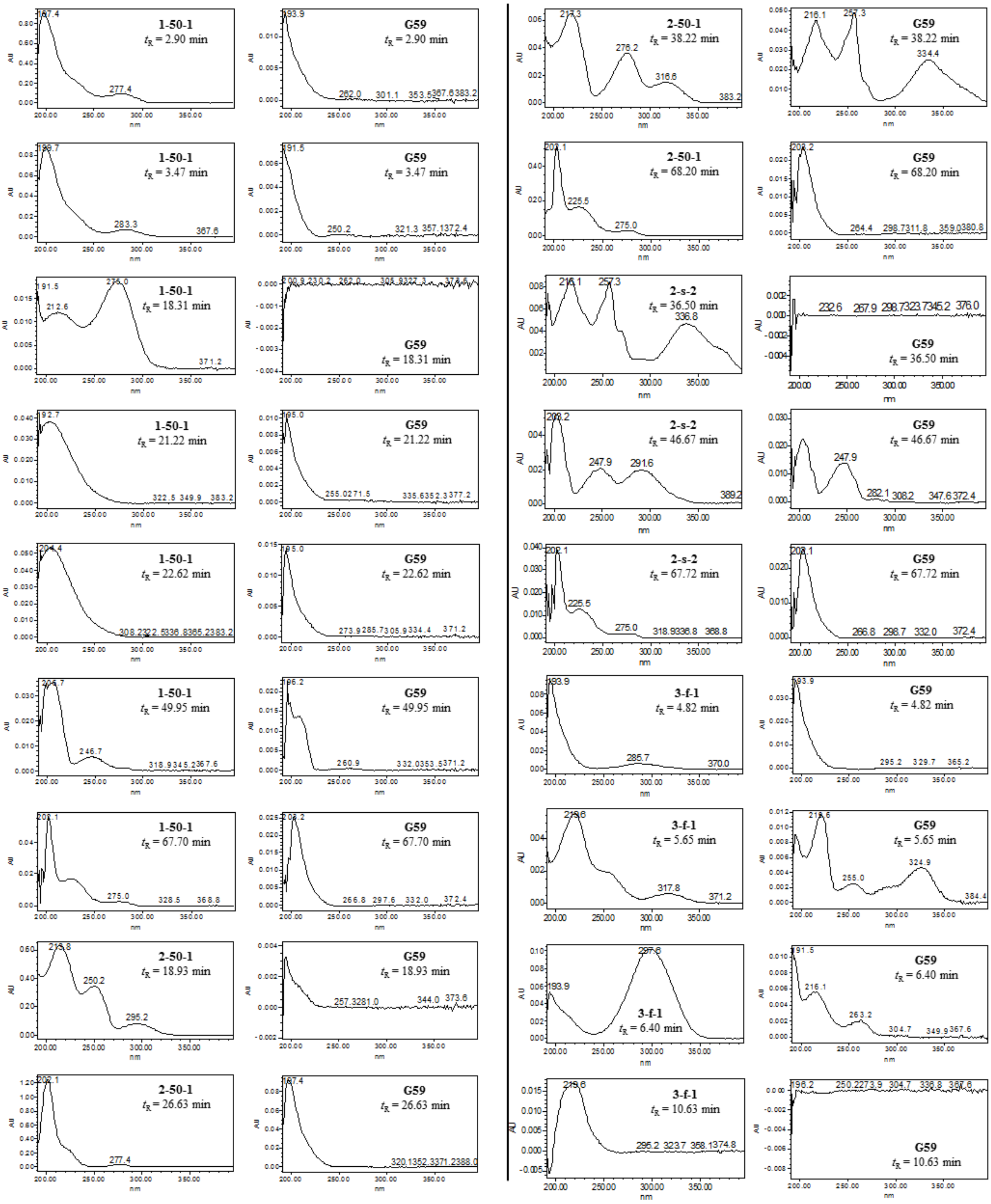


**(A2)**

**Figure S1.** *Cont.*

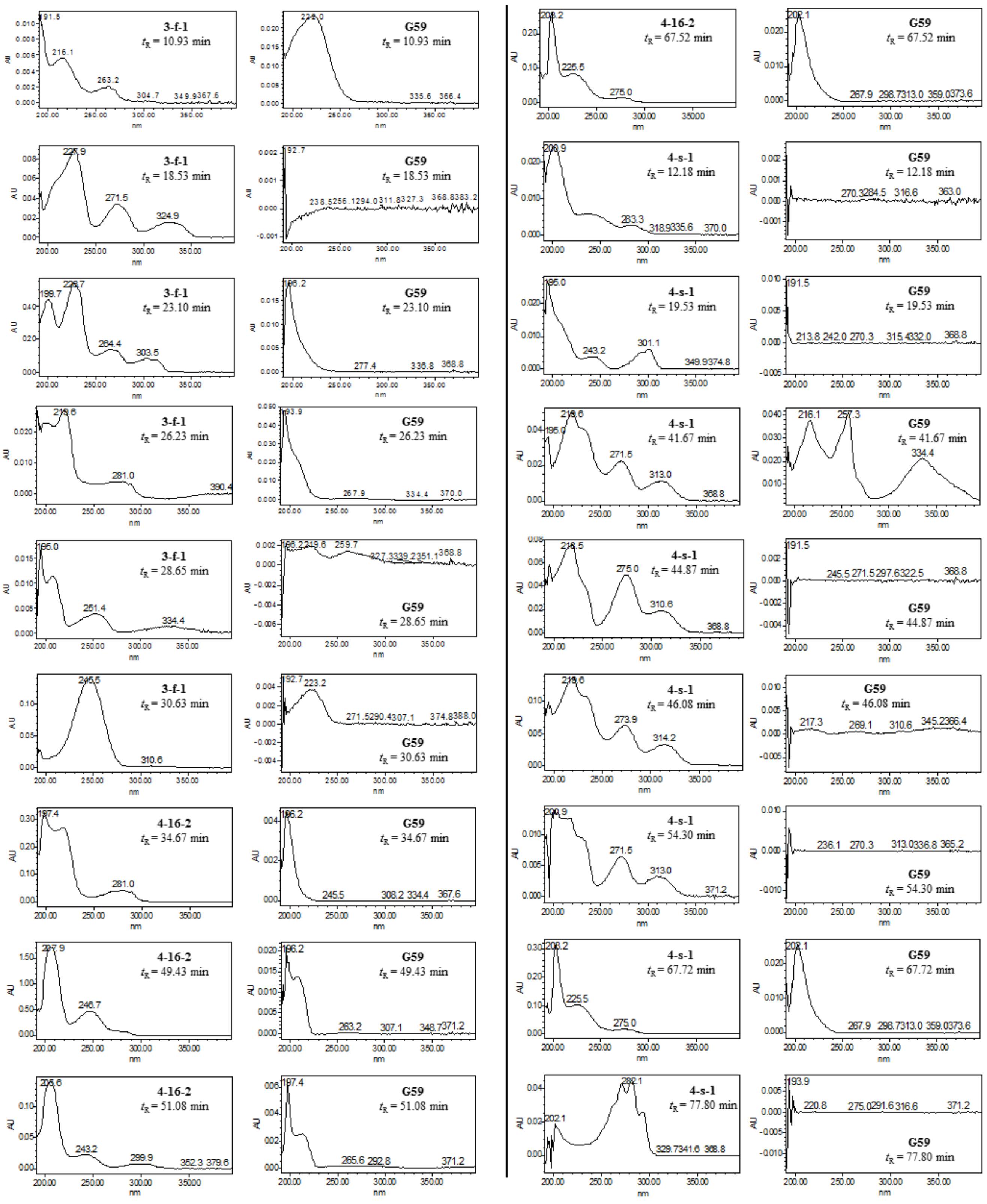


**(A2)**

**Figure S1.** *Cont.*

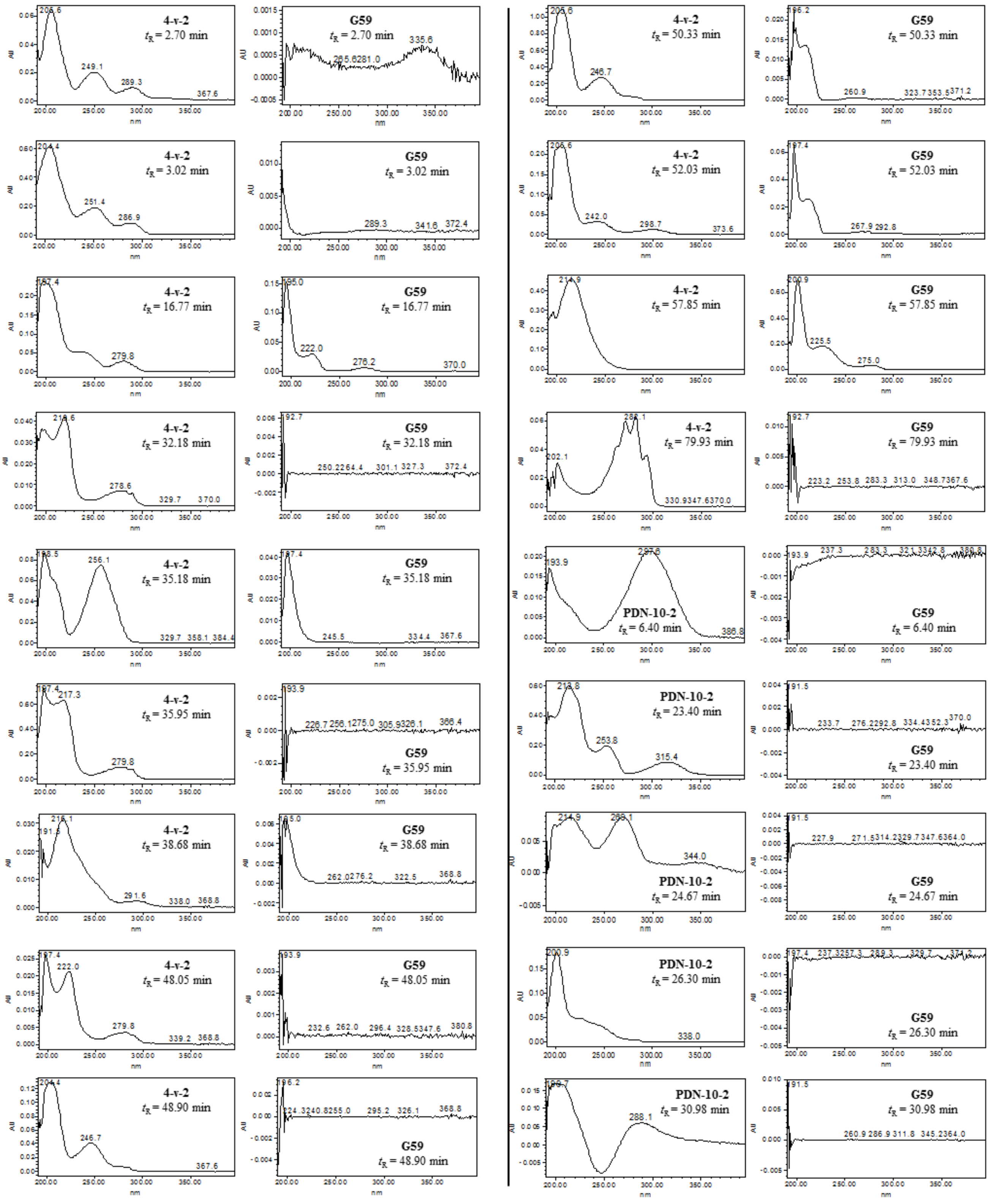


**(A2)**

**Figure S1.** *Cont.*

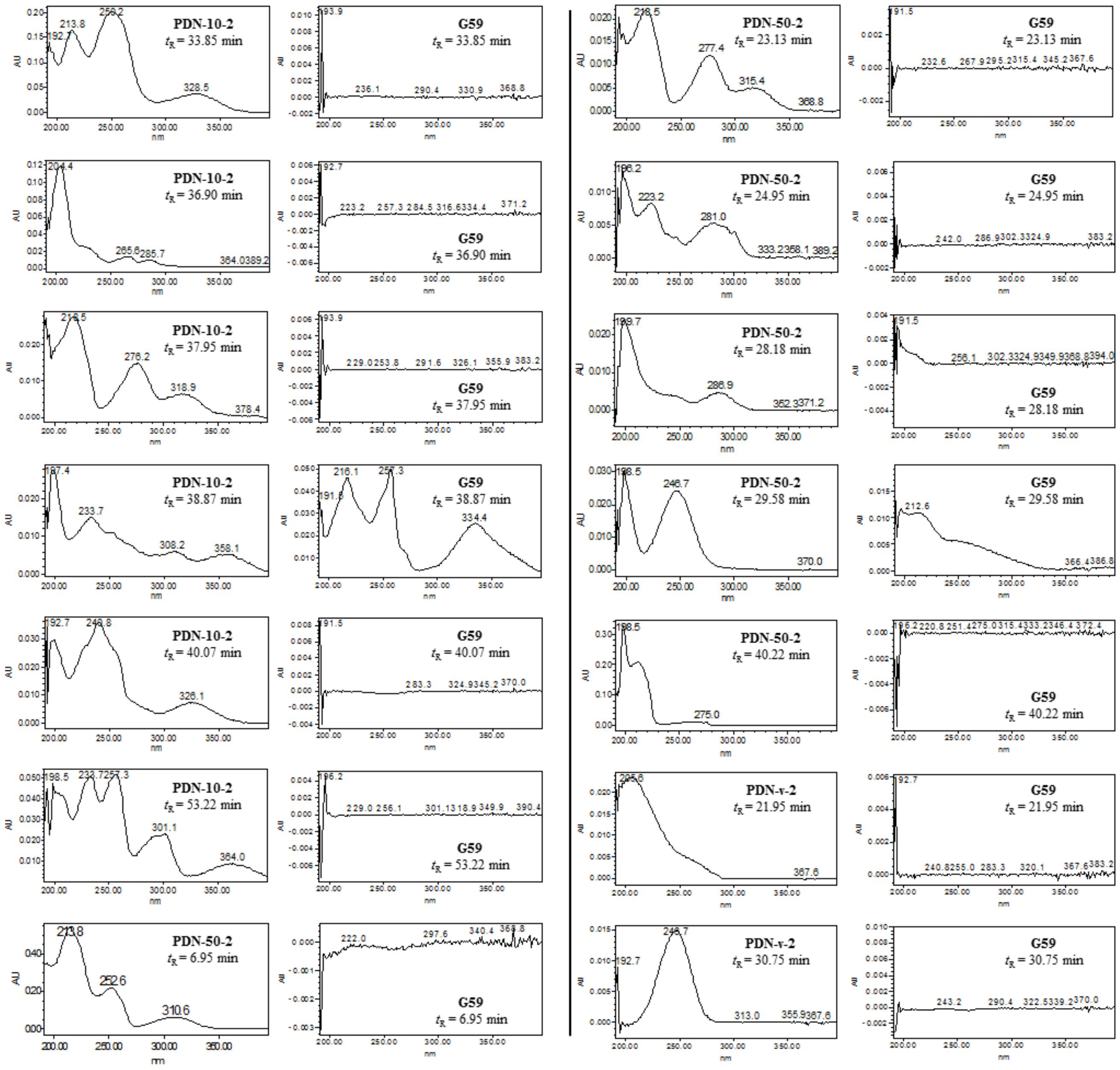


**(A2)**

**Figure S1.** *Cont.*

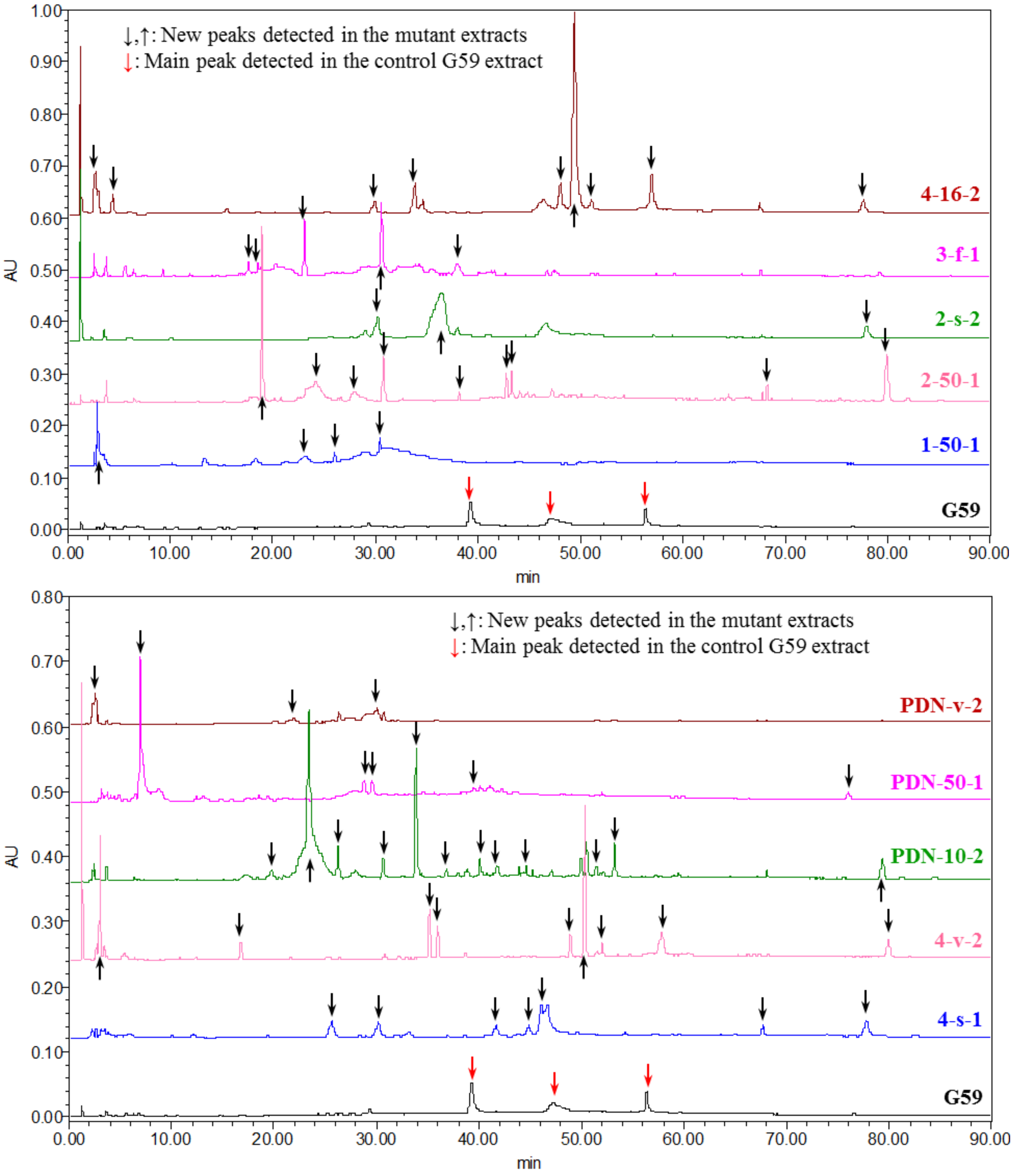


**(B1)**

**Figure S1.** *Cont.*

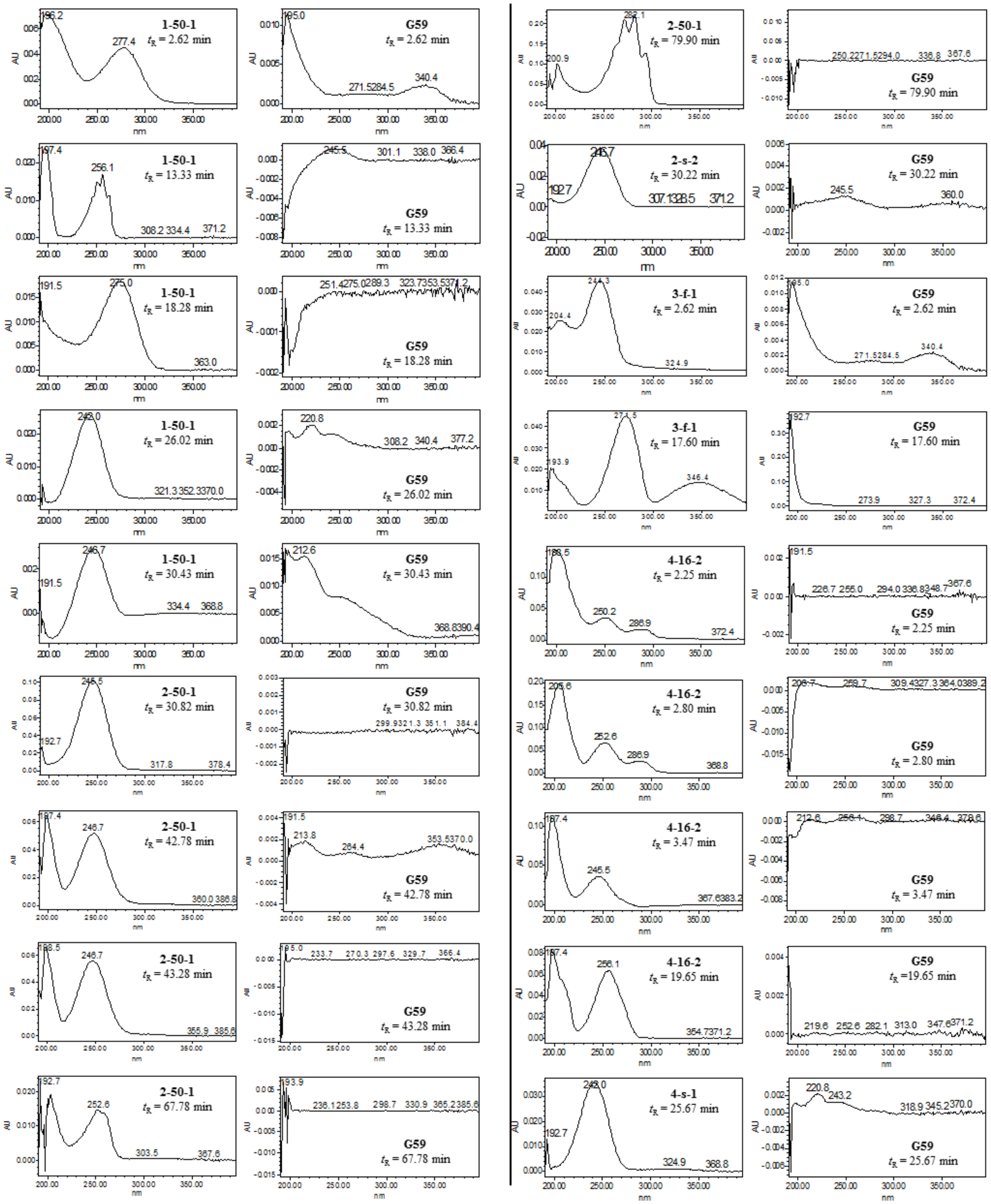


**(B2)**

**Figure S1.** *Cont.*

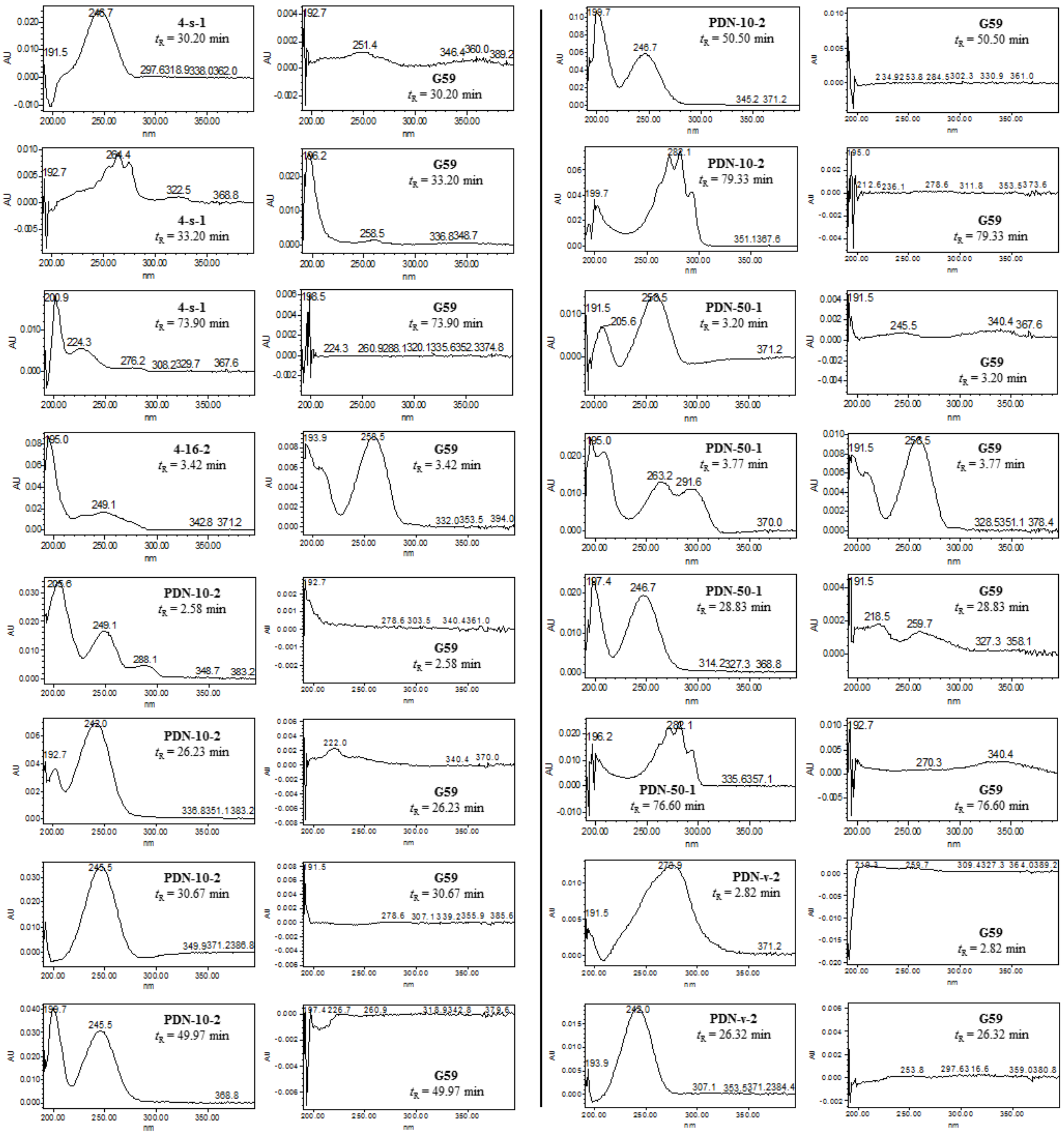


**(B2)**

**Figure S1.** *Cont.*

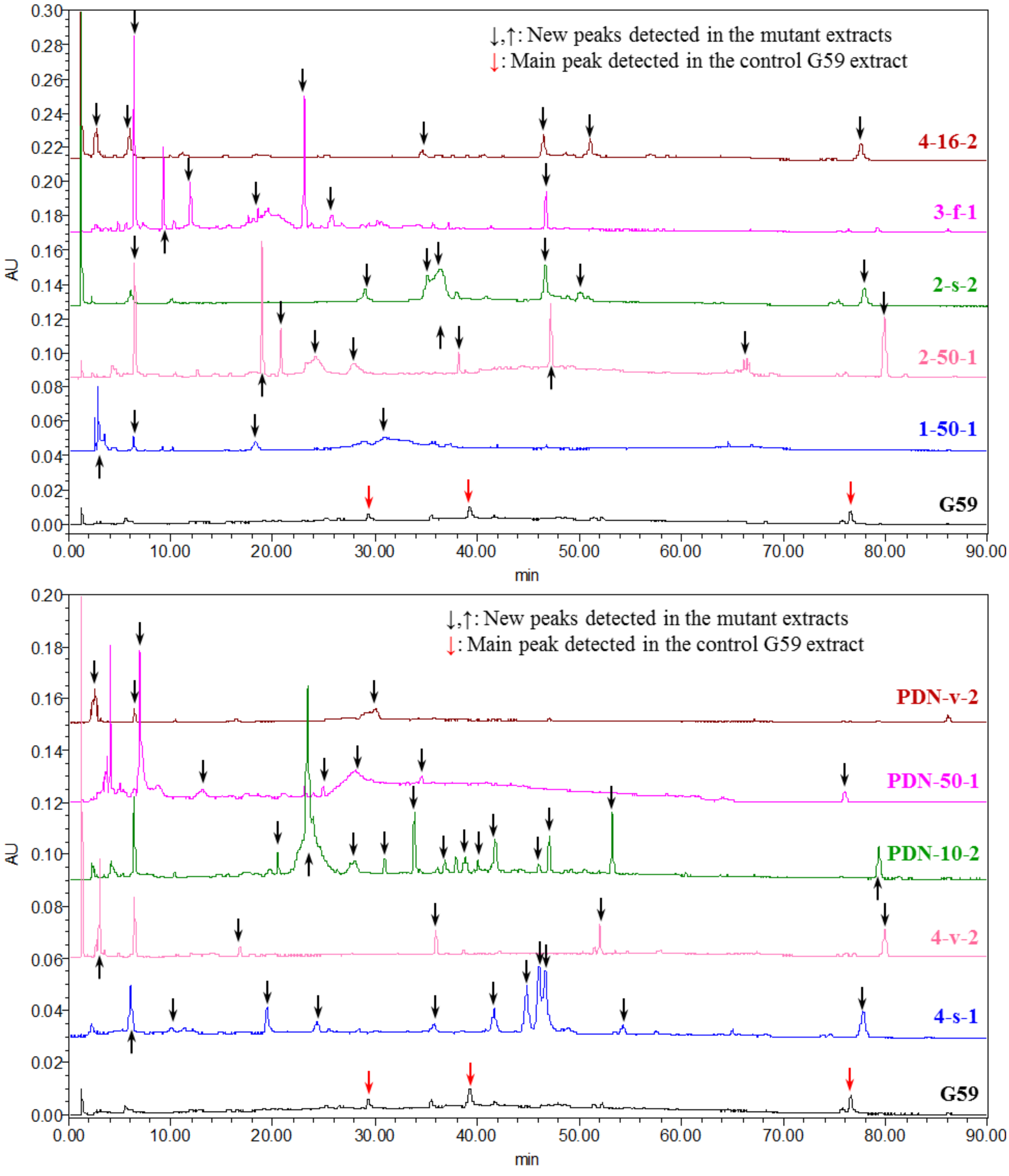


**(C1)**

**Figure S1.** *Cont.*

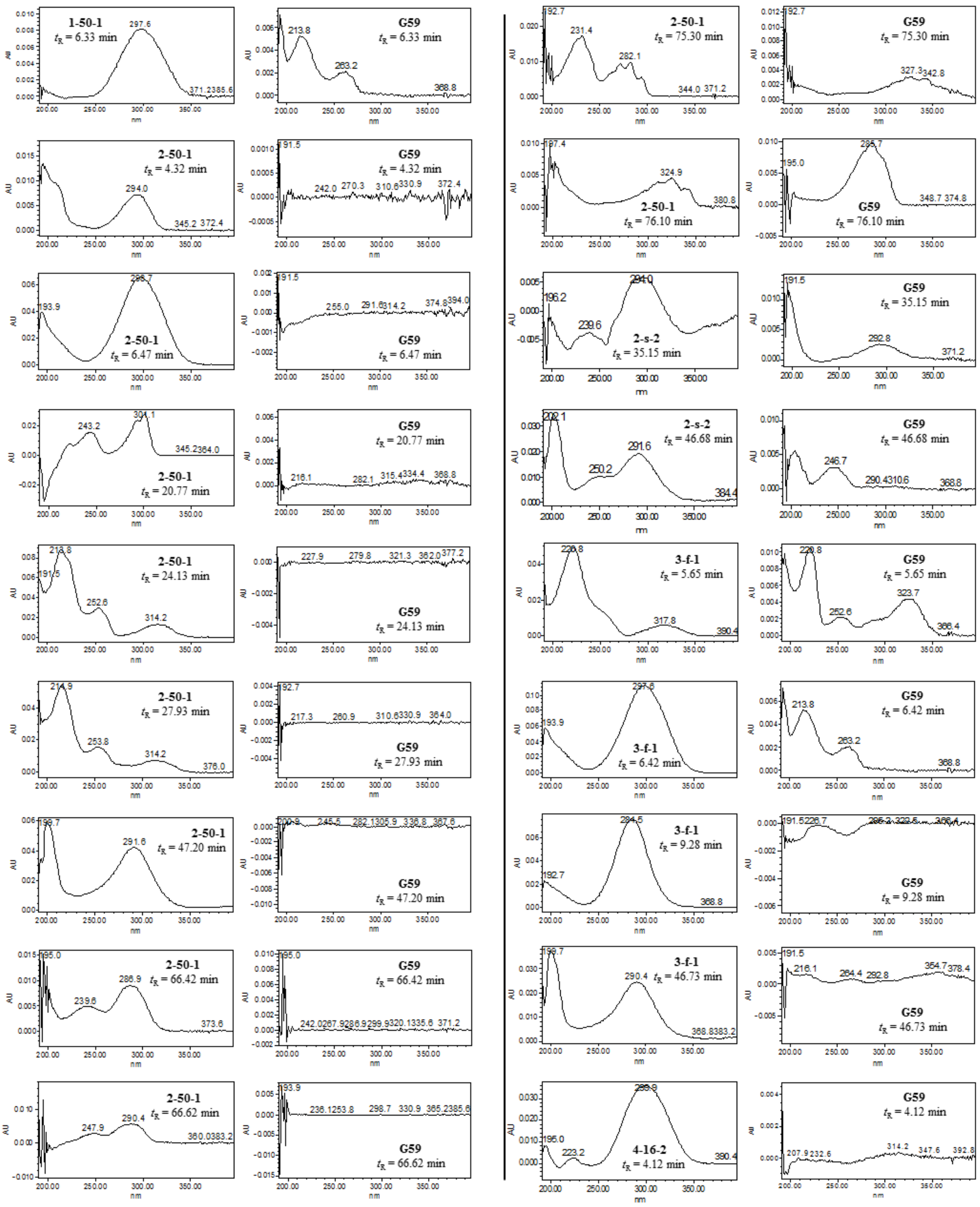


**(C2)**

**Figure S1.** *Cont.*

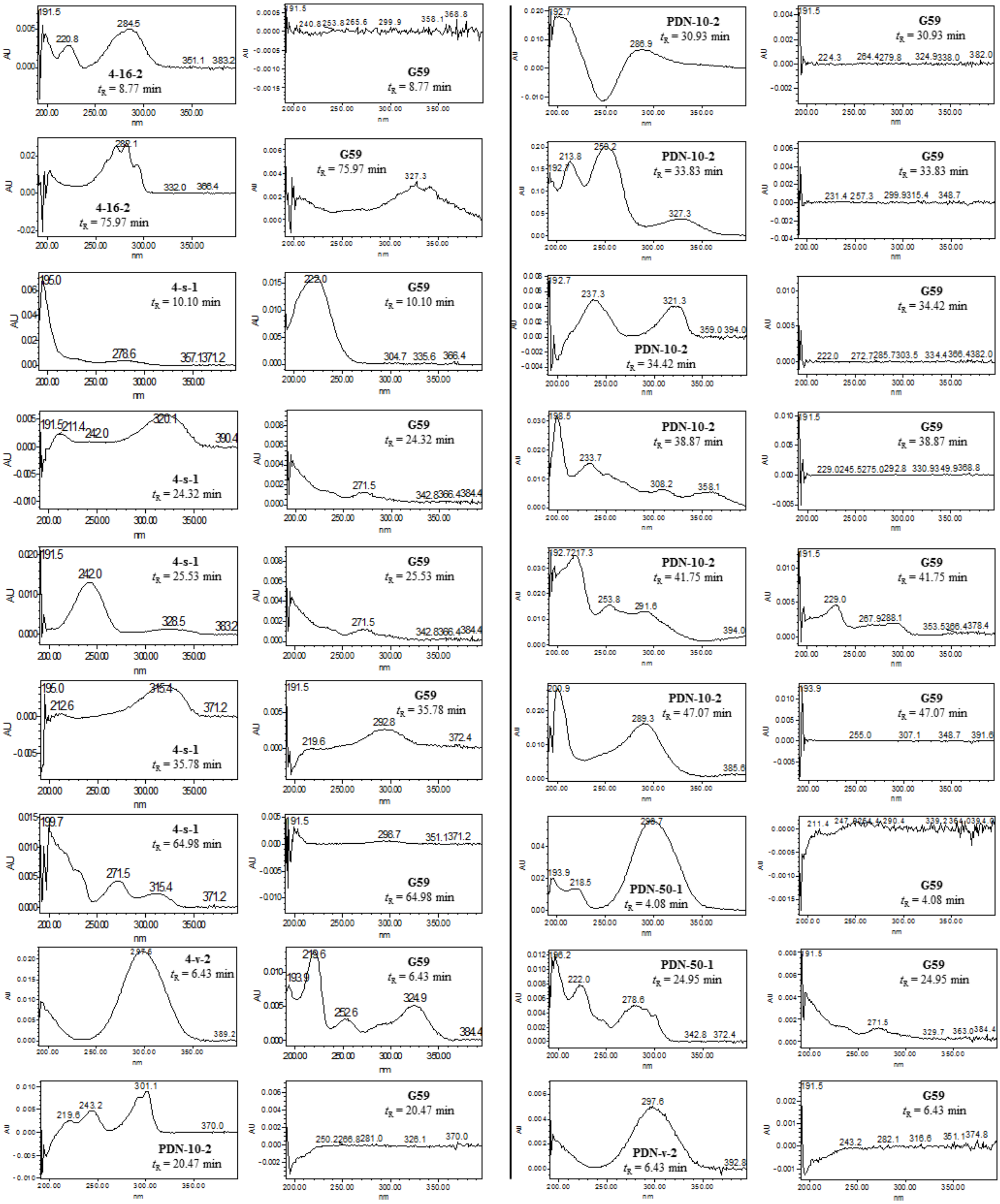


**(C2)**

**Figure S1.** *Cont.*

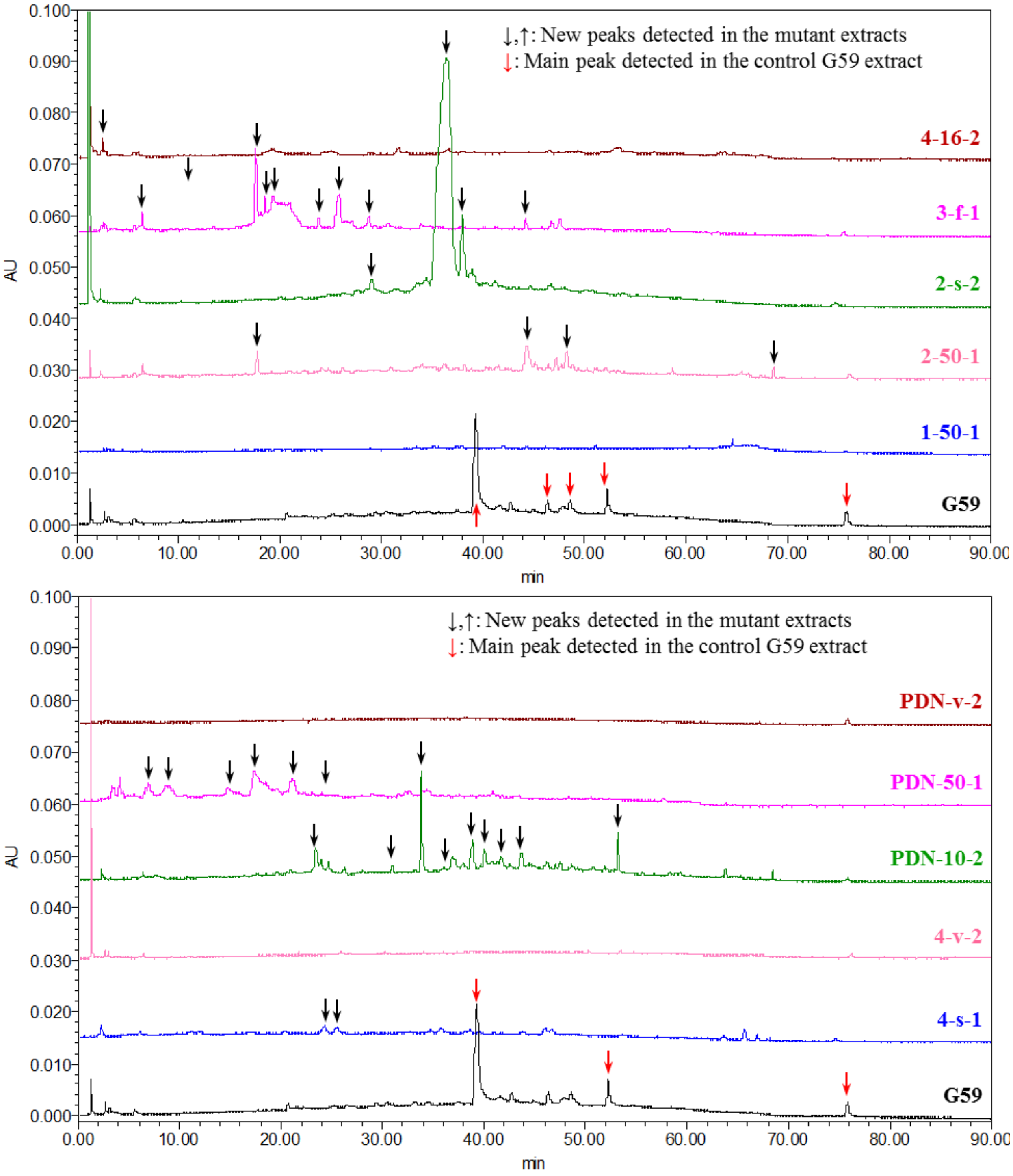


**(D1)**

**Figure S1.** *Cont.*

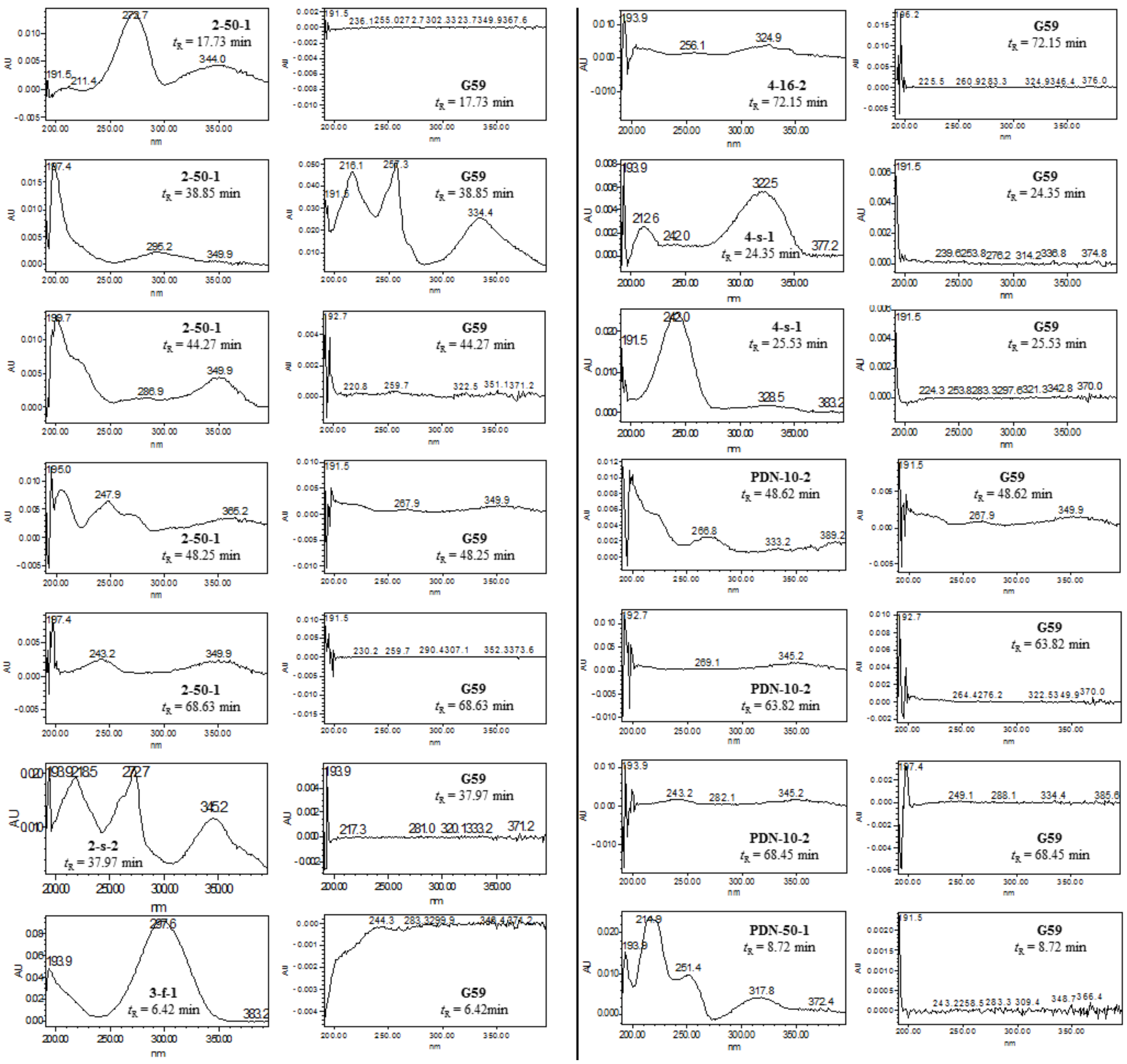


(**D2**)

**Figure S1.** *Cont.*

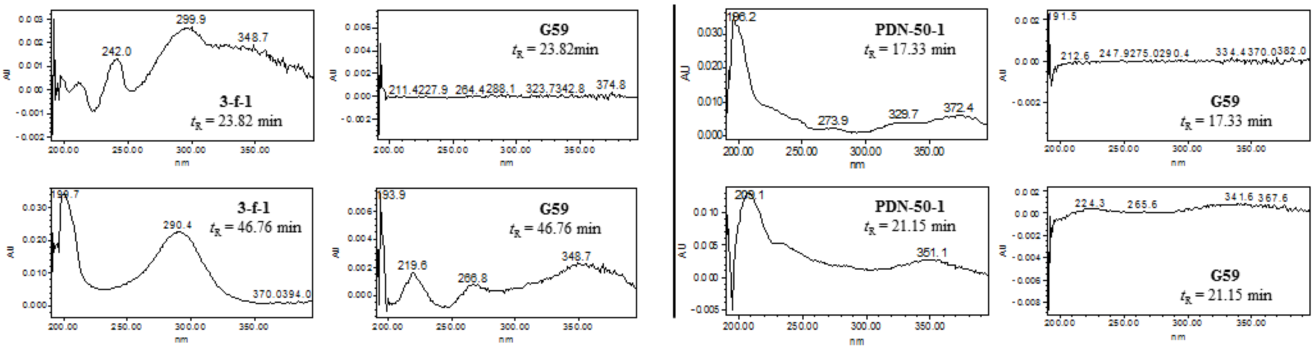


(**D2**)

**Figure S1.** HPLC-PDAD-UV analysis of the EtOAc extracts of control G59 strain and its selected mutants. (**A1**) HPLC profiles detected at 210 nm; (**A2**) UV spectra of the new peaks in mutant extracts in the HPLC profiles at 210 nm, and UV absorptions of the control G59 extract at the corresponding retention times; (**B1**) HPLC profiles detected at 254 nm; (**B2**) UV spectra of the new peaks in mutant extracts in the HPLC profiles at 254 nm, and UV absorptions of the control G59 extract at the corresponding retention times; (**C1**) HPLC profiles detected at 300 nm; (**C2**) UV spectra of the new peaks in mutant extracts in the HPLC profiles at 300 nm, and UV absorptions of the control G59 extract at the corresponding retention times; (**D1**) HPLC profiles detected at 350 nm; (**D2**) UV spectra of the new peaks in mutant extracts in the HPLC profiles at 350 nm, and UV absorptions of the control G59 extract at the corresponding retention times.

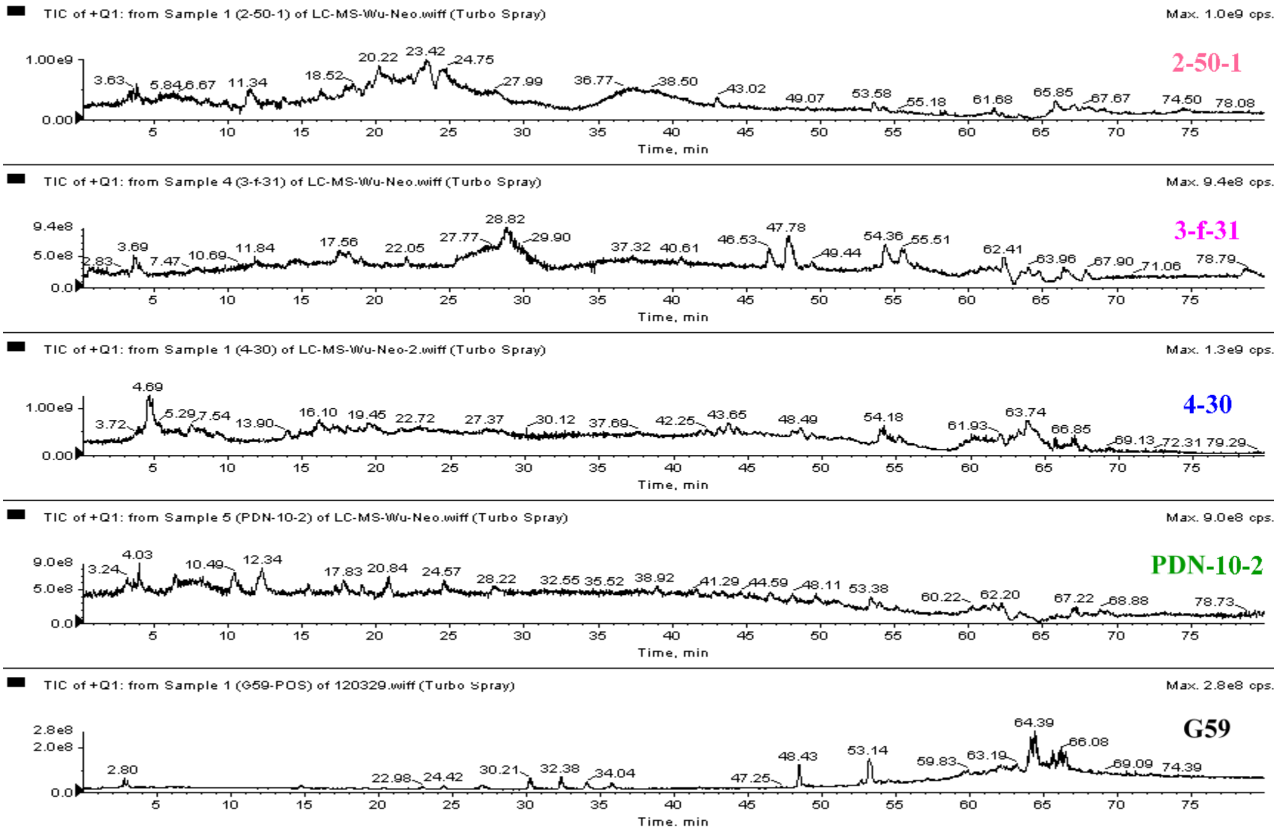


(**A1**)

**Figure S2.** *Cont.*

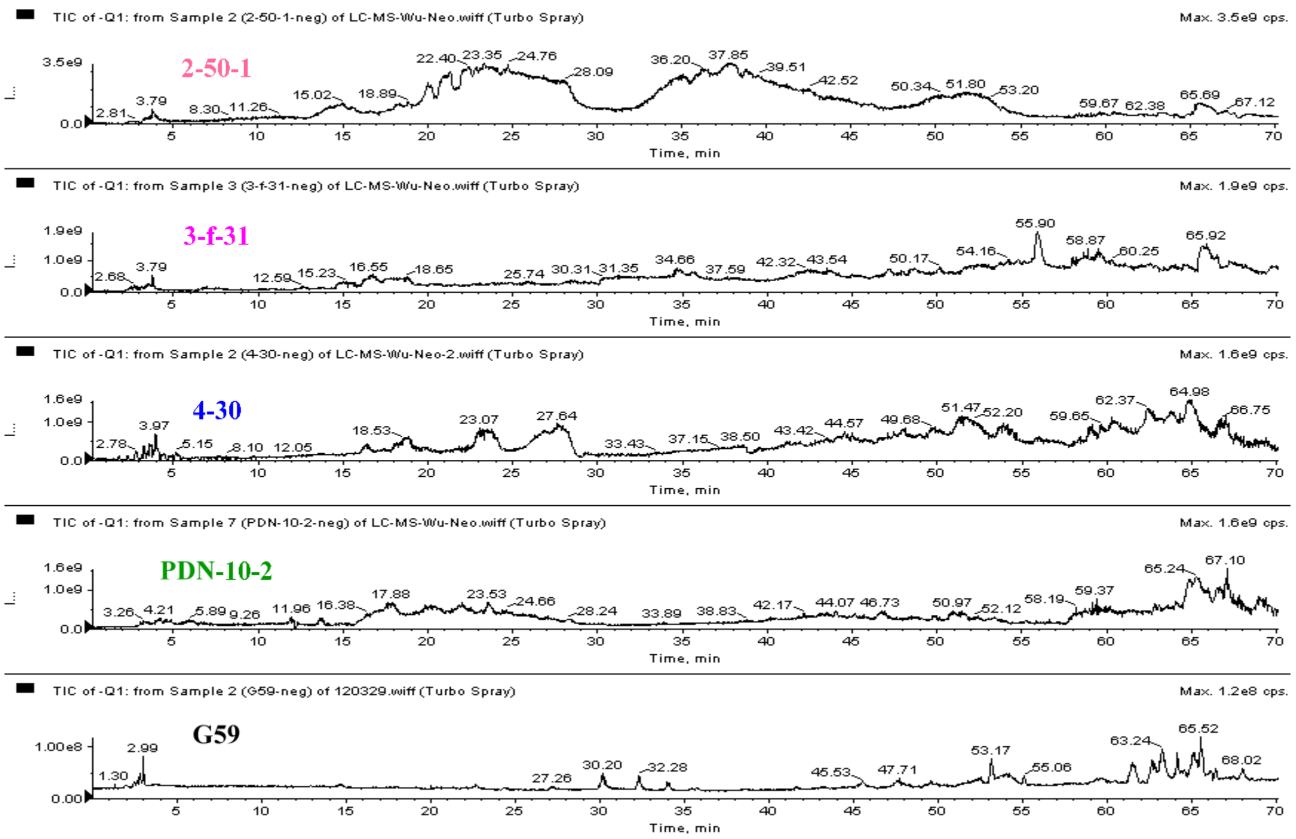


**(A2)**

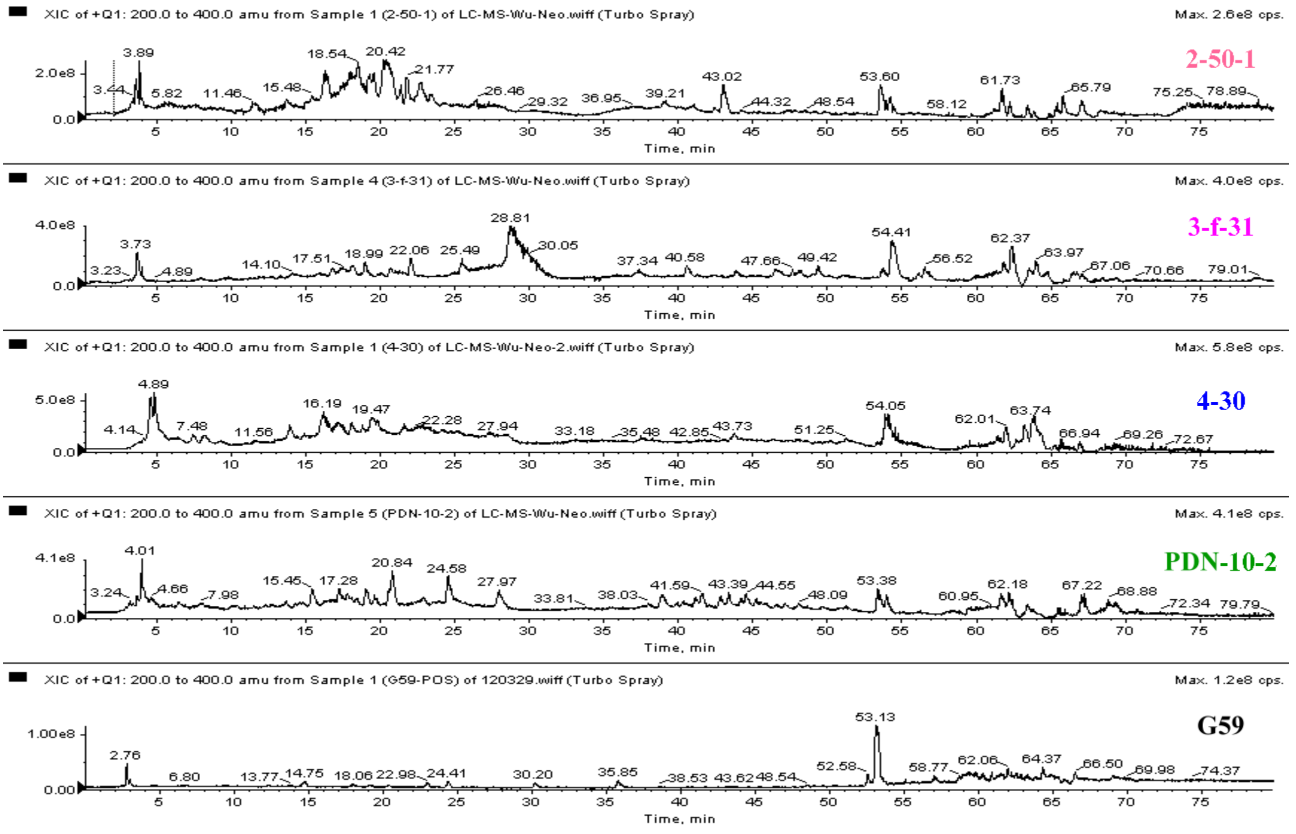


**(B1)**

**Figure S2.** *Cont.*

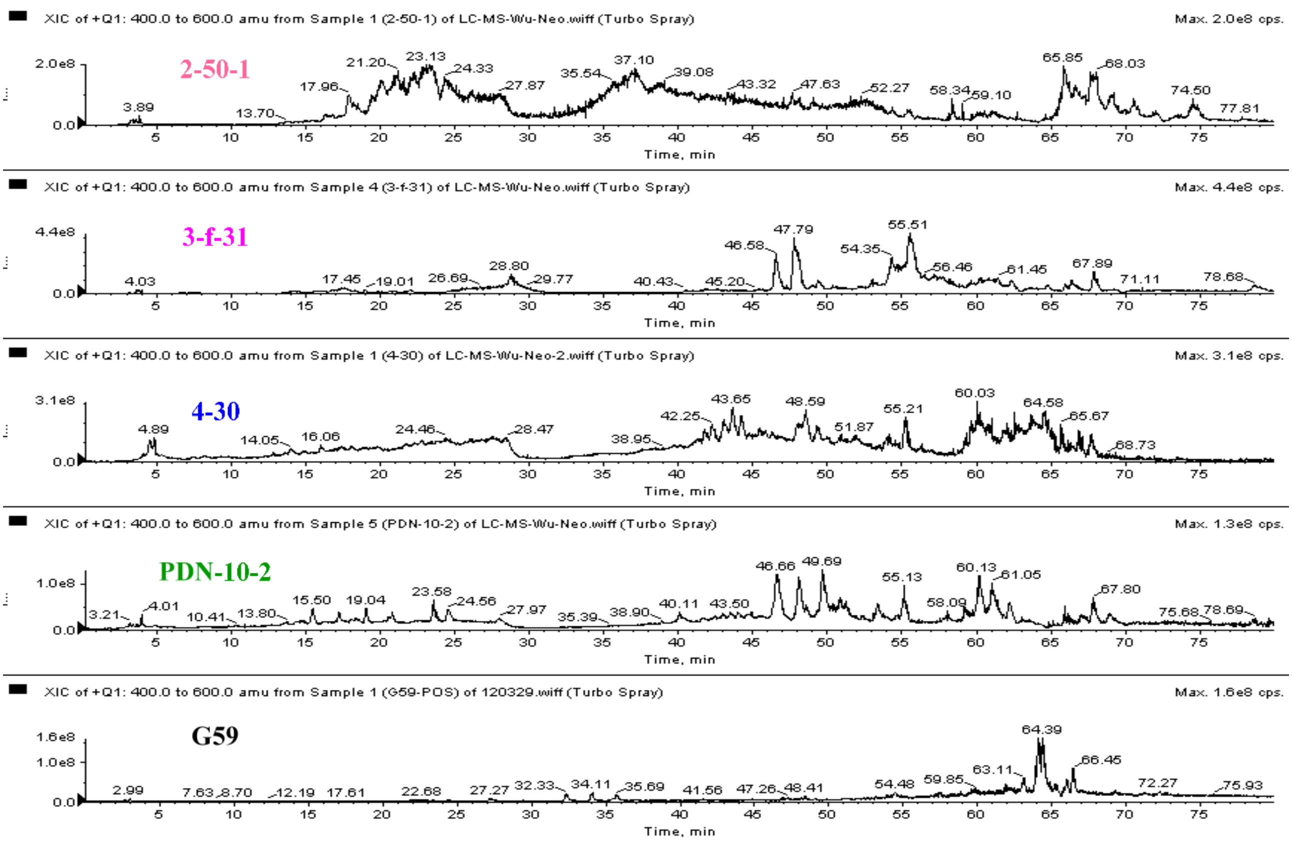


**(B2)**

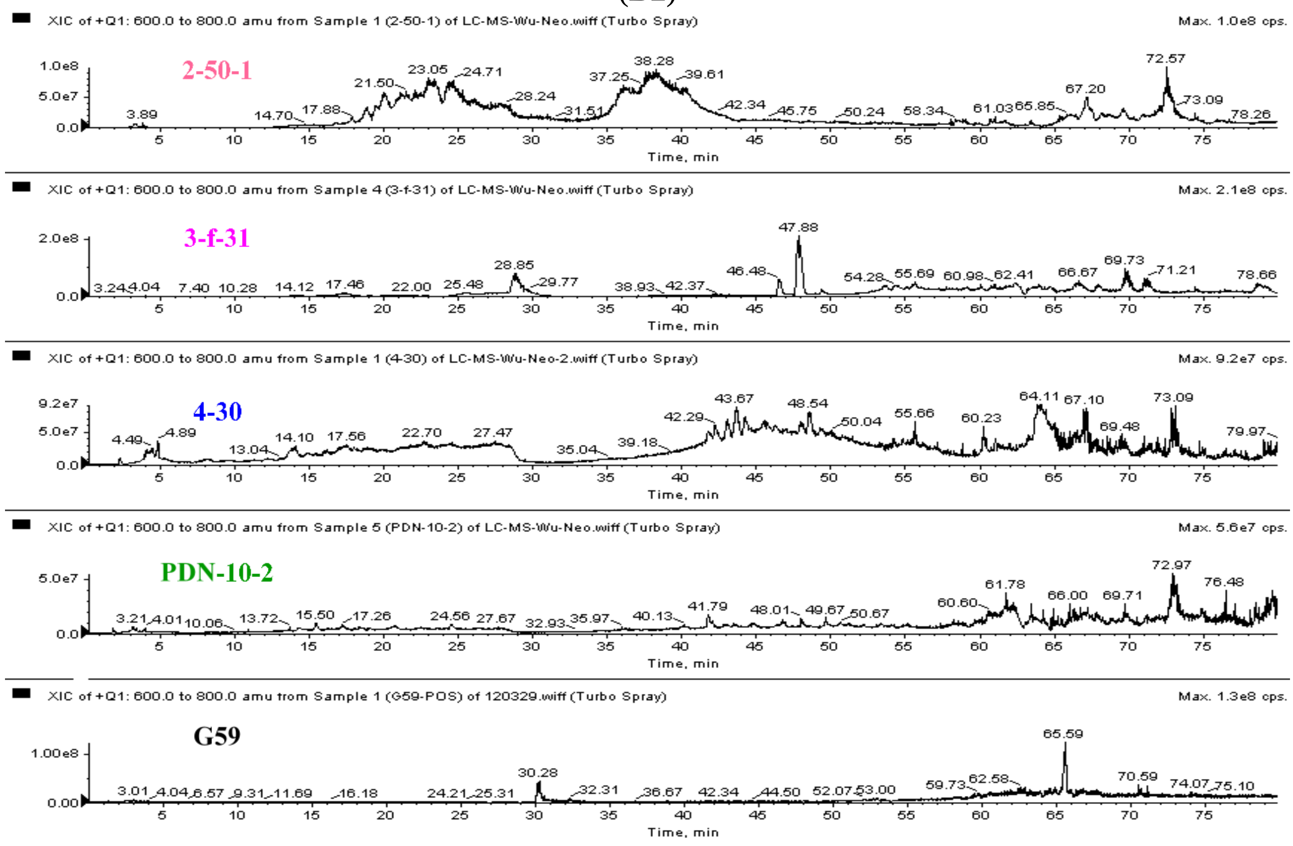


**(B3)**

**Figure S2.** *Cont.*

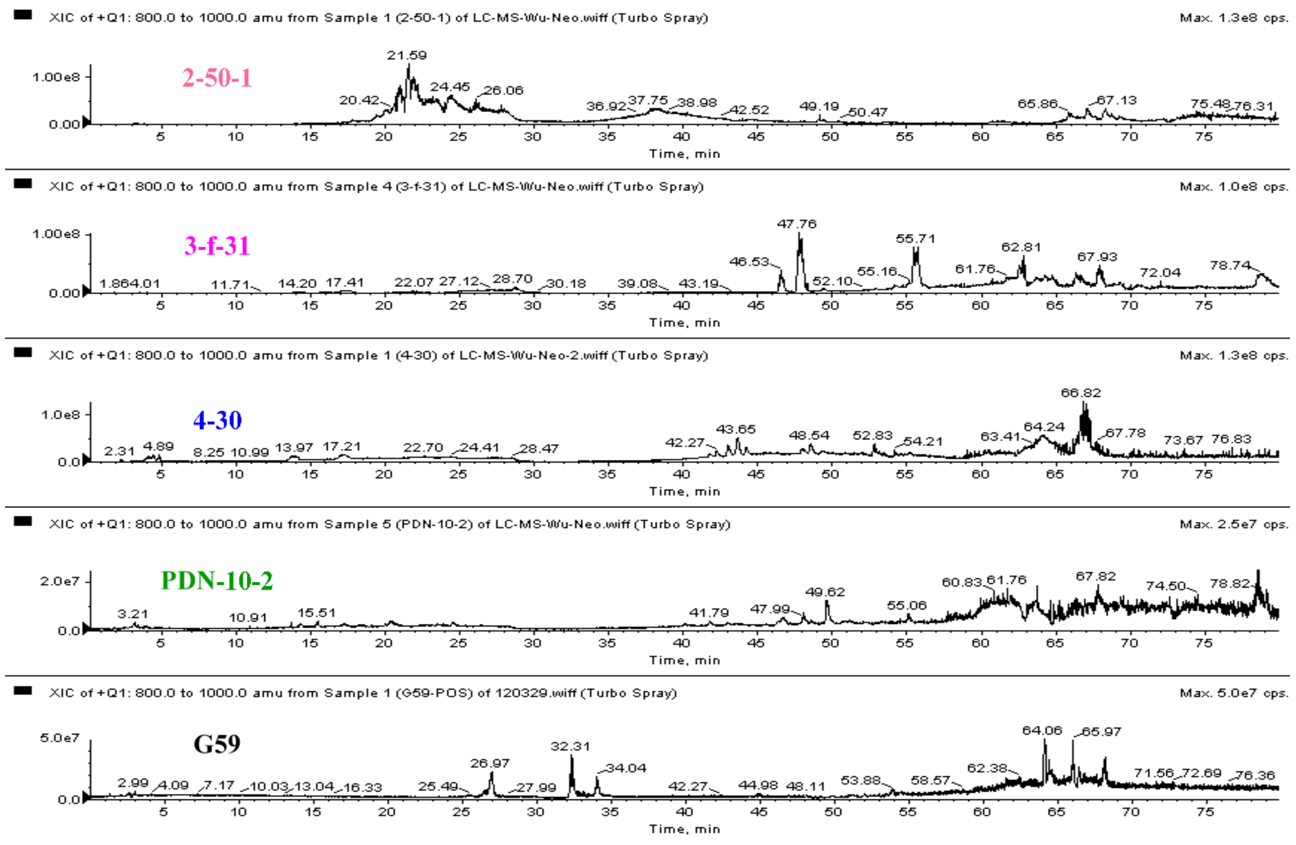


(B4)

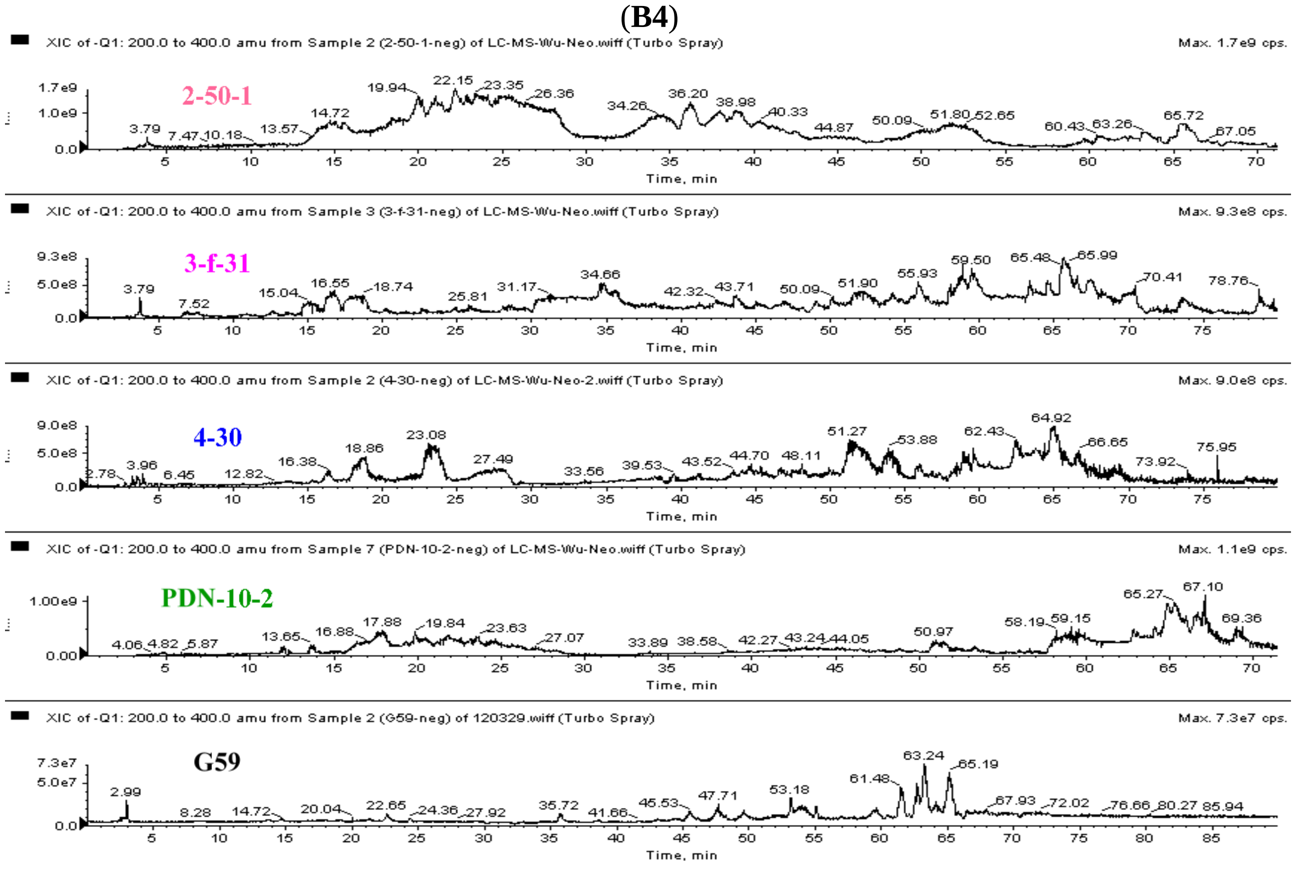


(C1)

**Figure S2.** *Cont.*

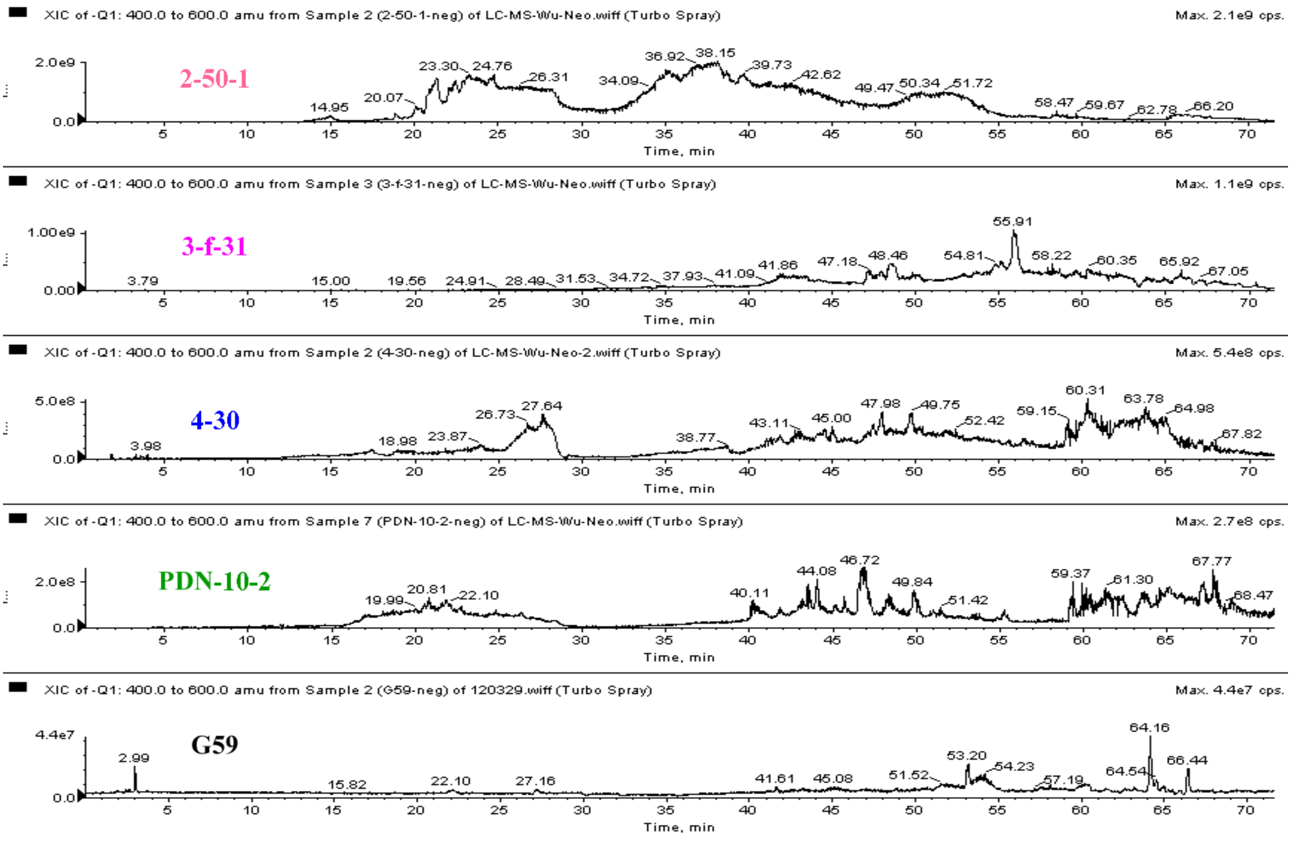


**(C2)**

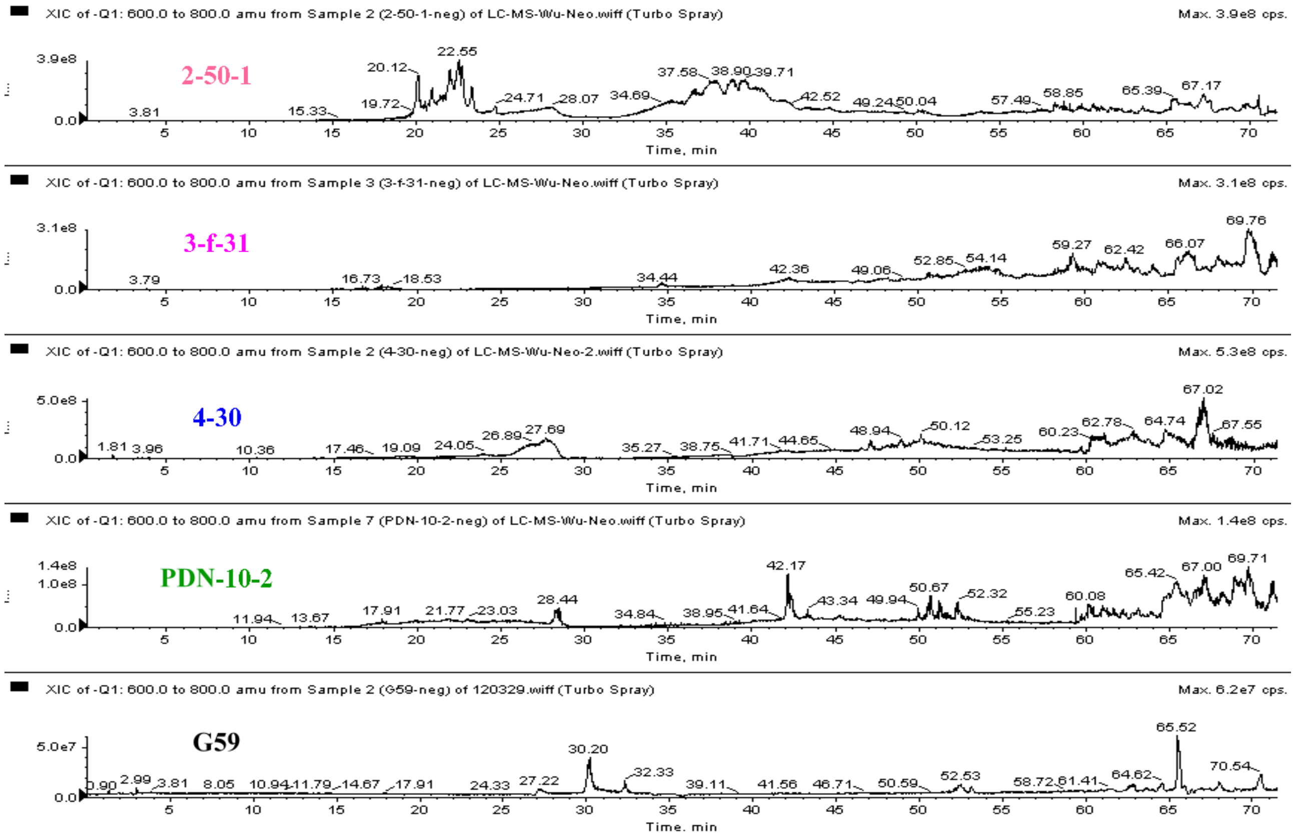


**(C3)**

**Figure S2.** *Cont.*

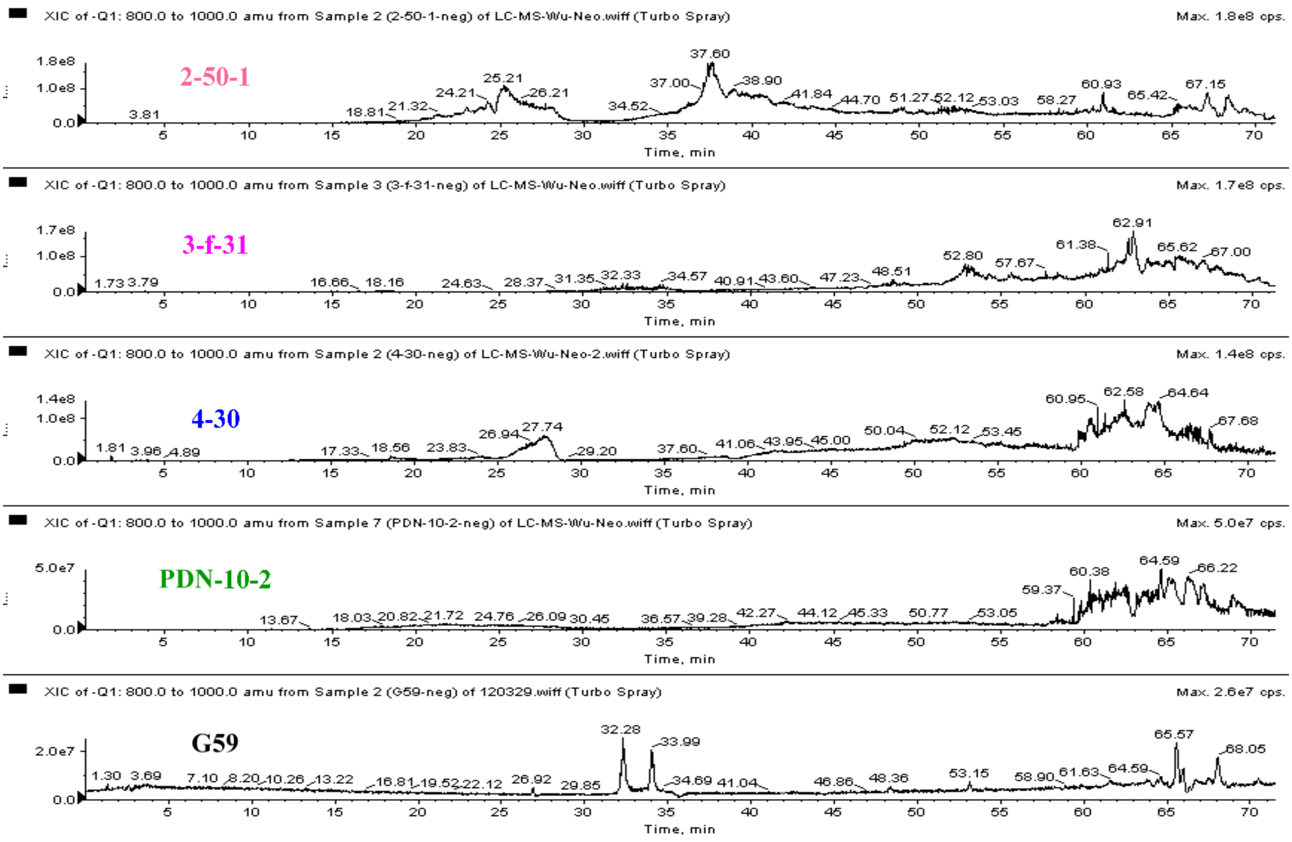


**(C4)**

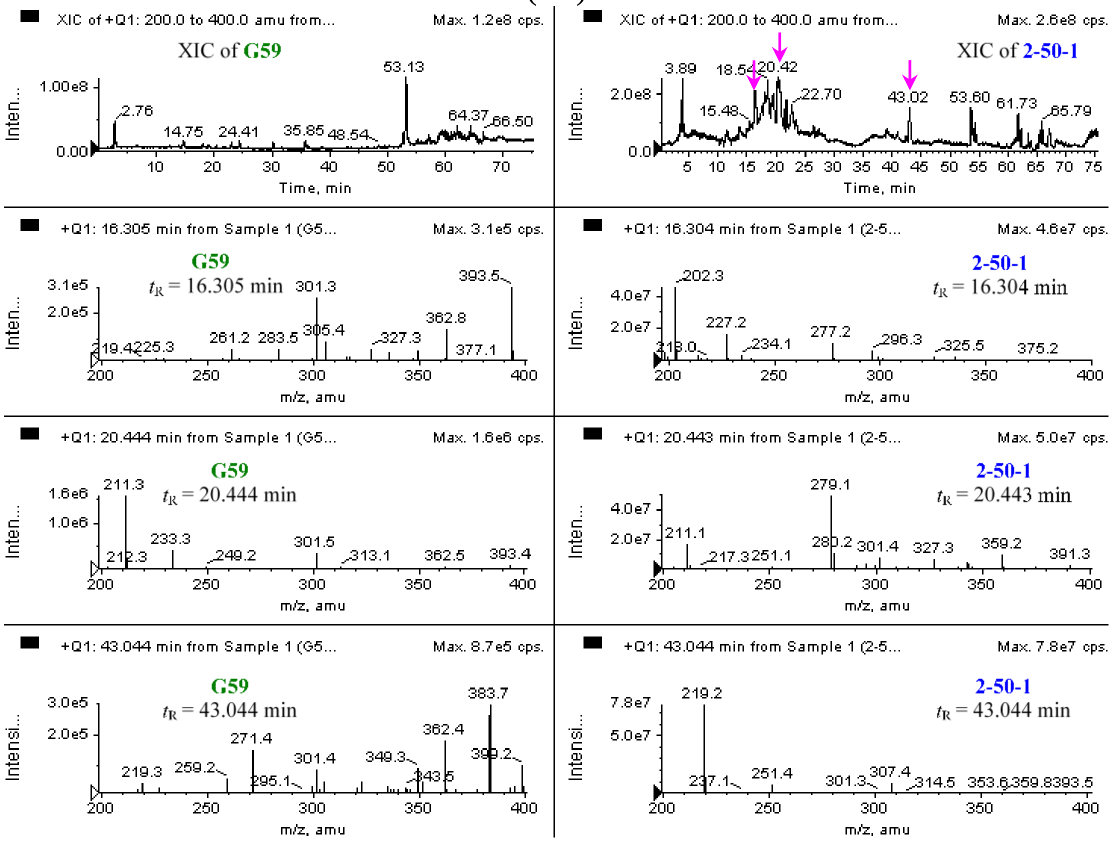


**(D1)**

**Figure S2.** *Cont.*

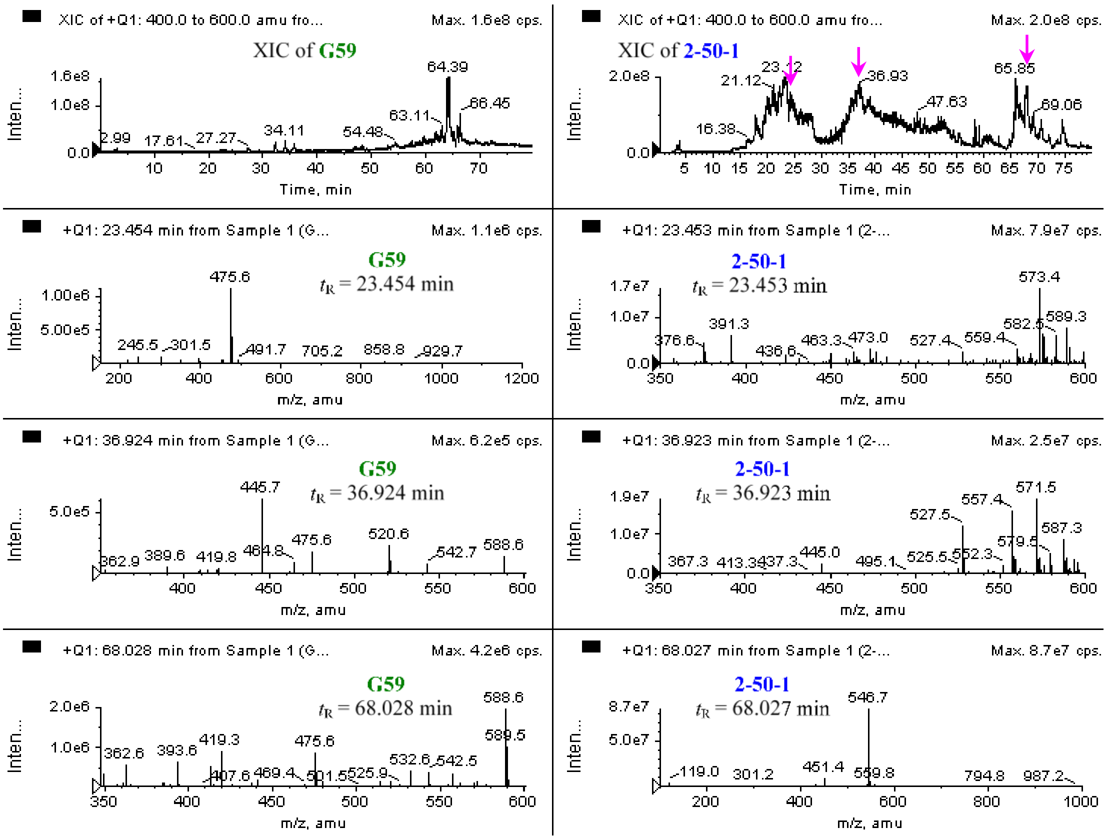


**(D2)**

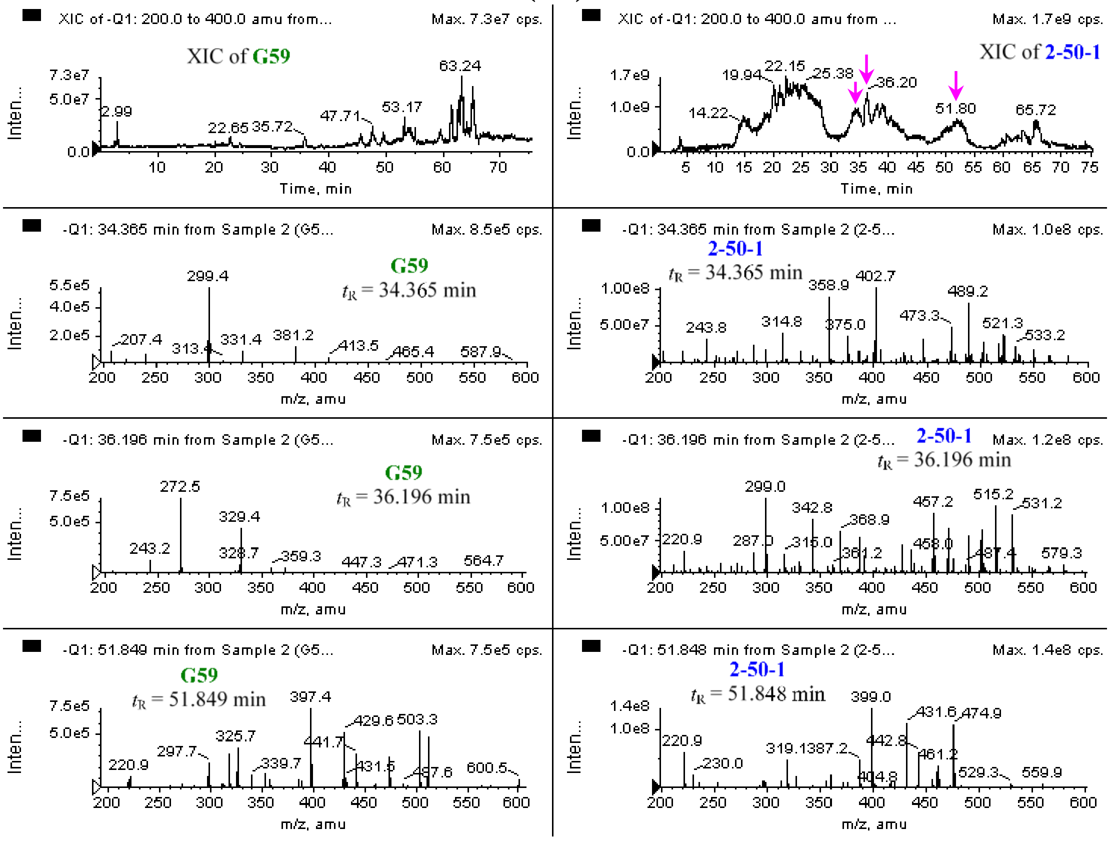


**(D3)**

**Figure S2.** *Cont.*

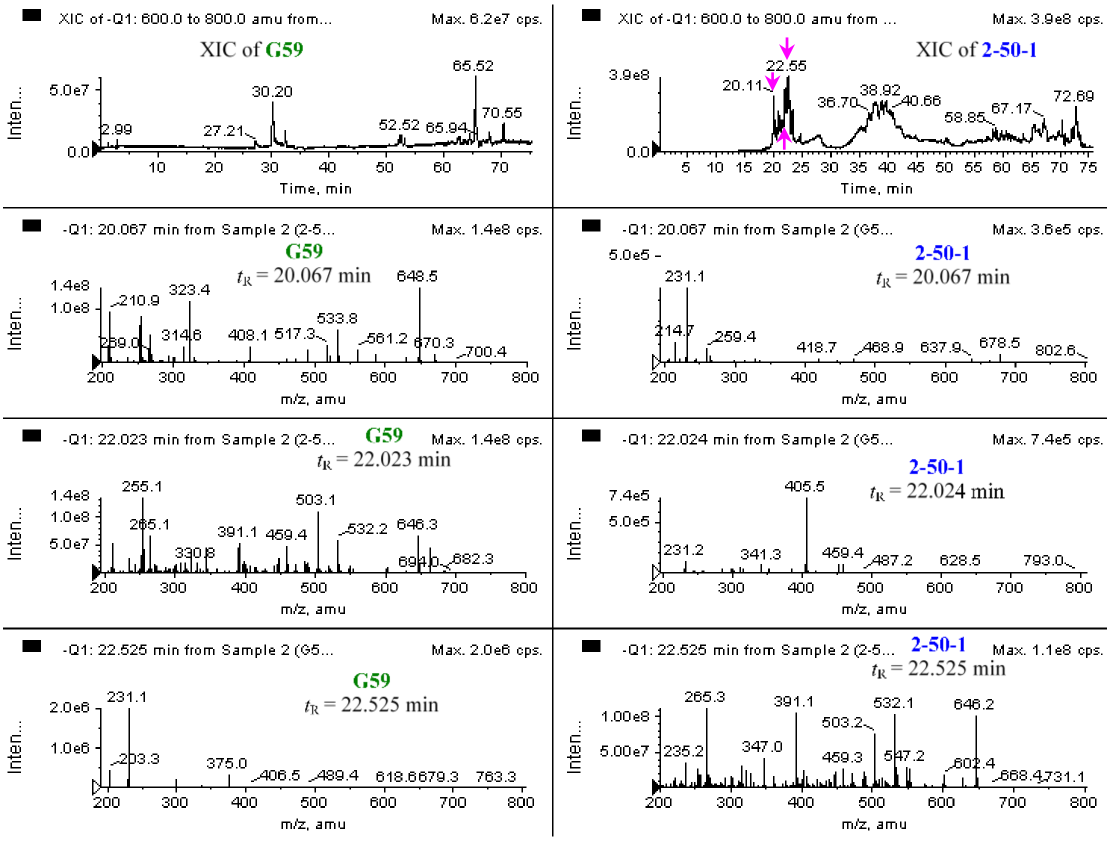


**(D4)**

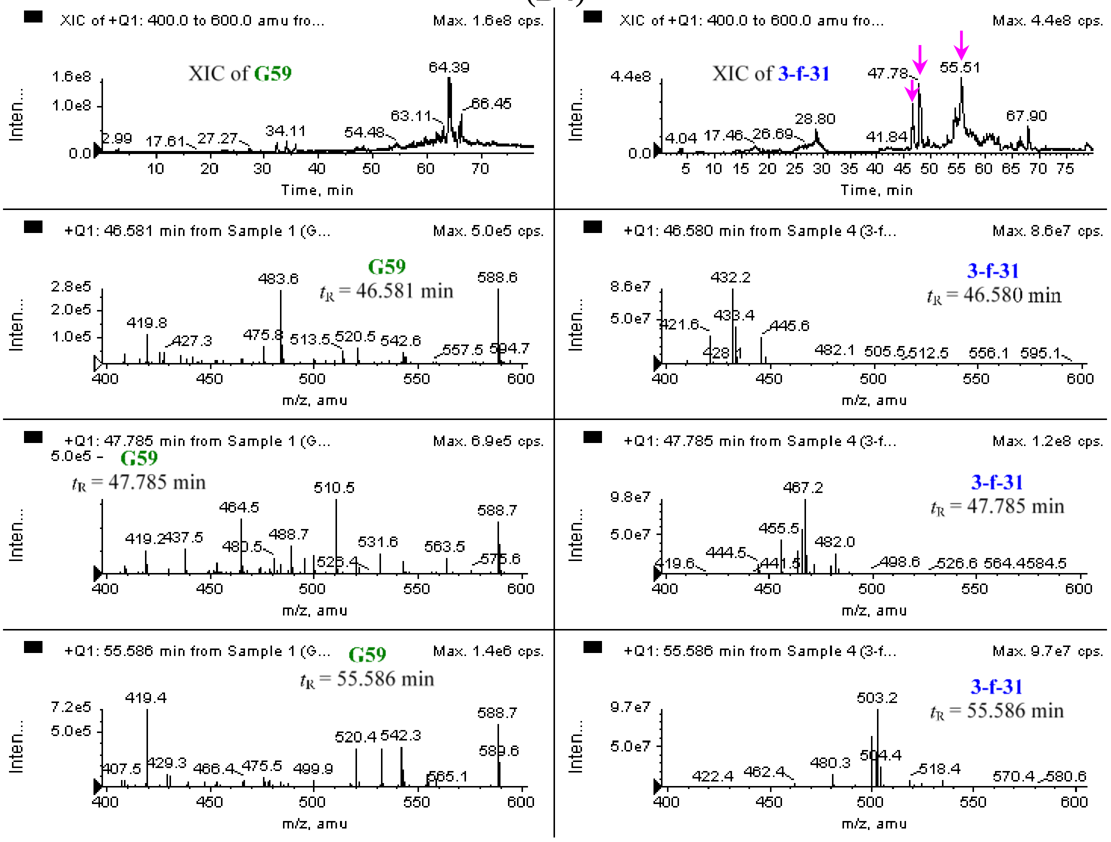


**(E1)**

**Figure S2.** *Cont.*

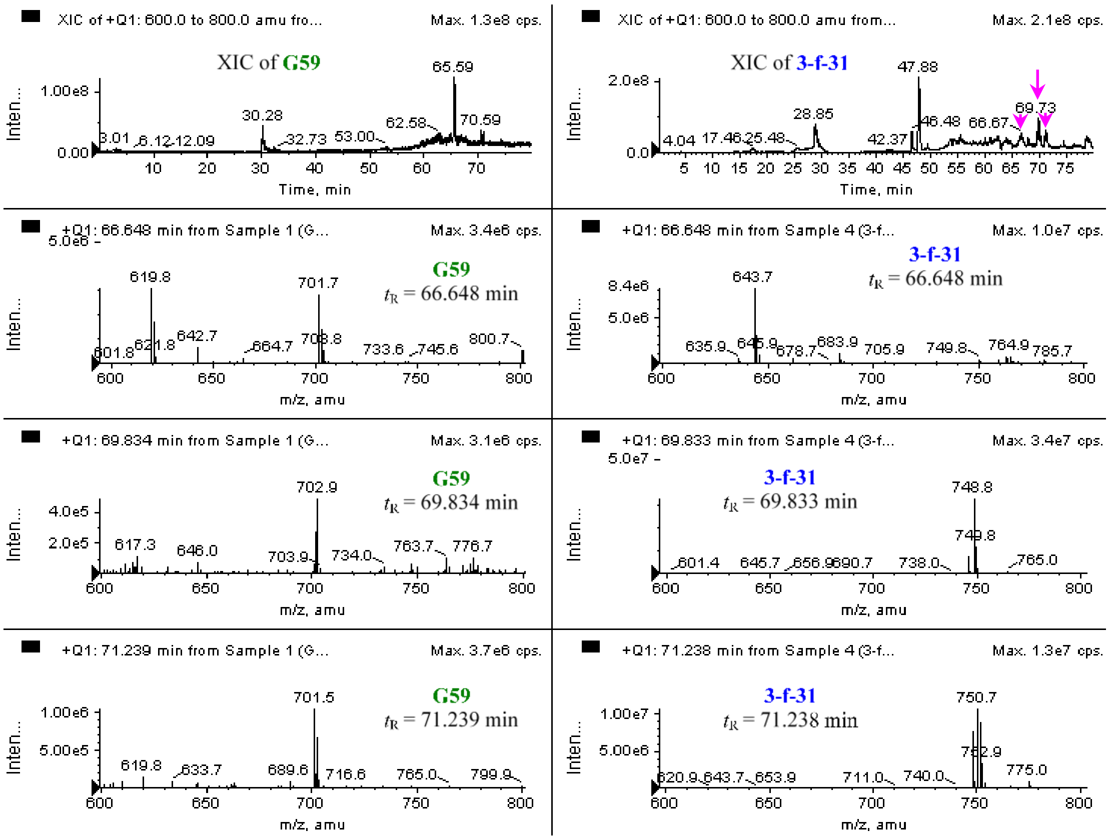


**(E2)**

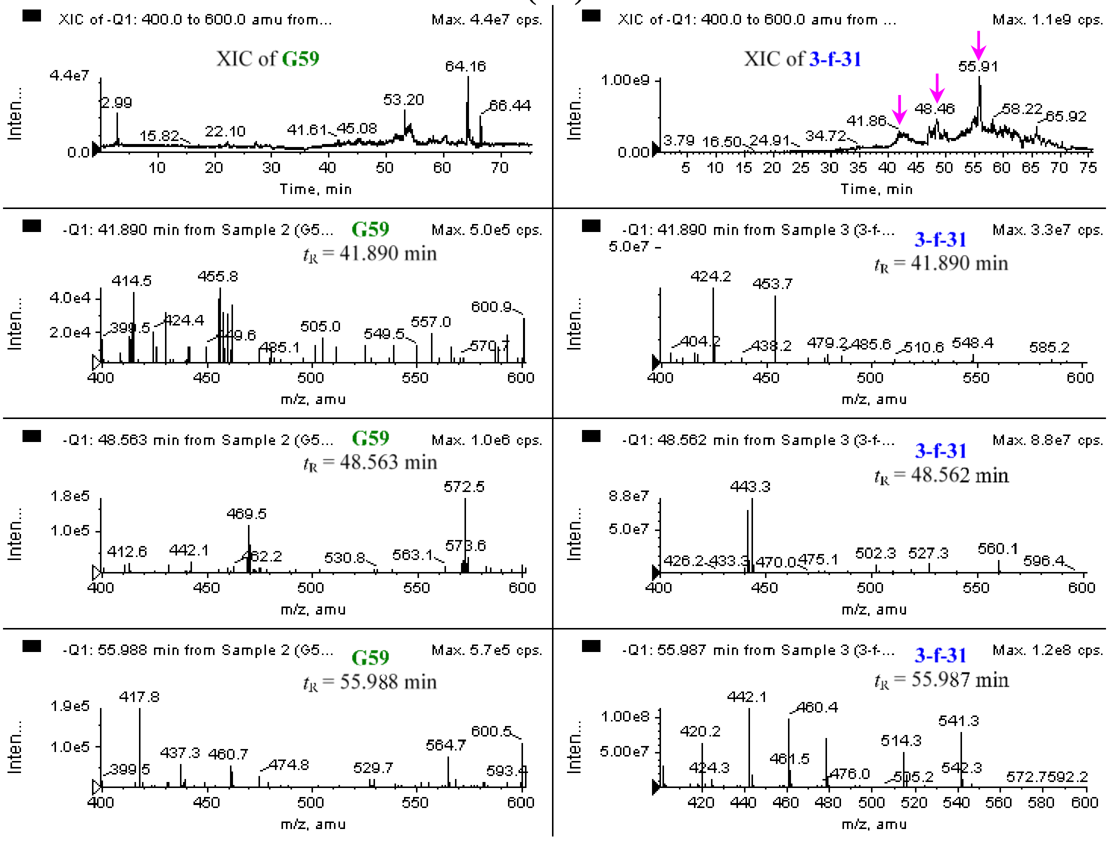


**(E3)**

**Figure S2.** *Cont.*

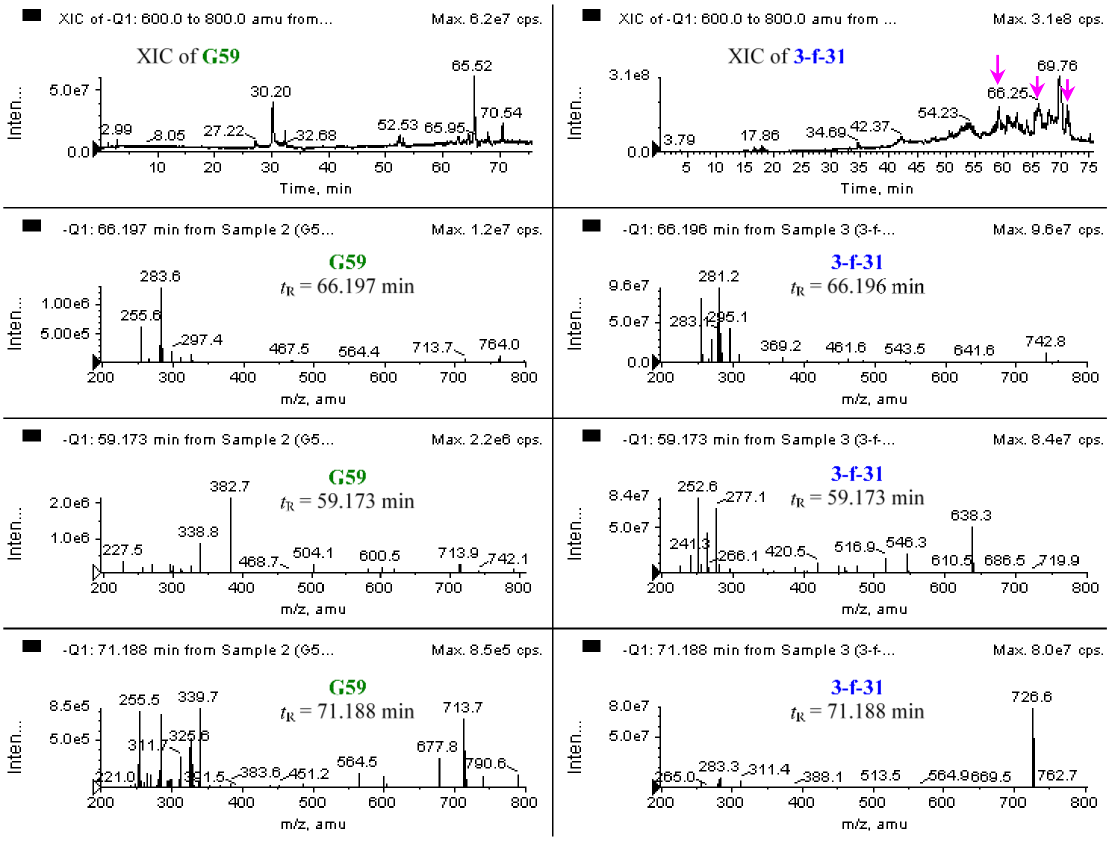


**(E4)**

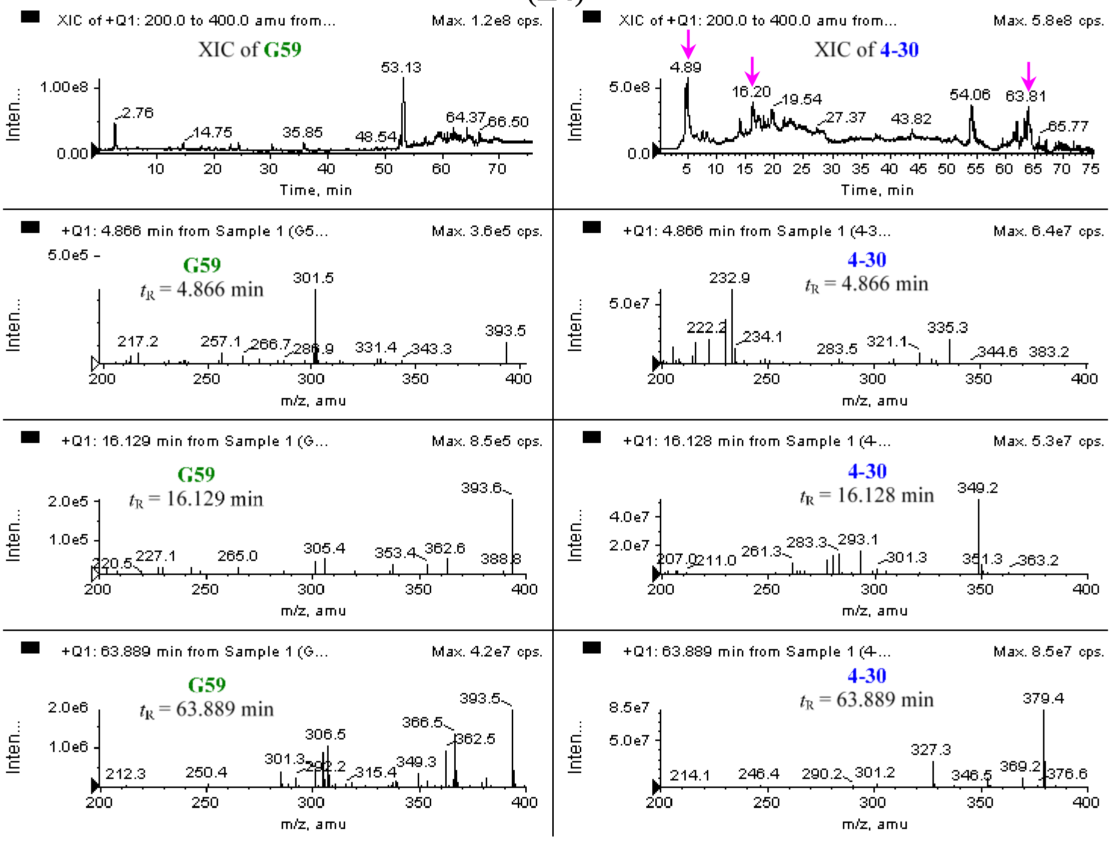


**(F1)**

**Figure S2.** *Cont.*

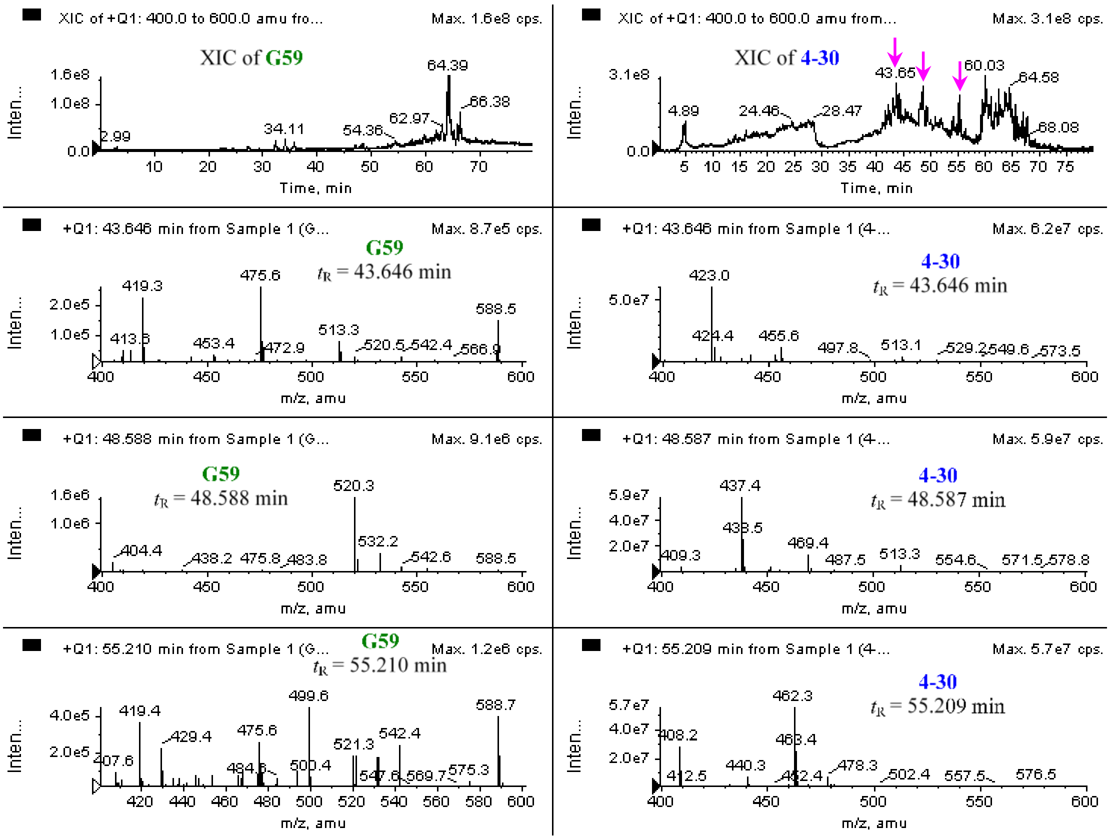


**(F2)**

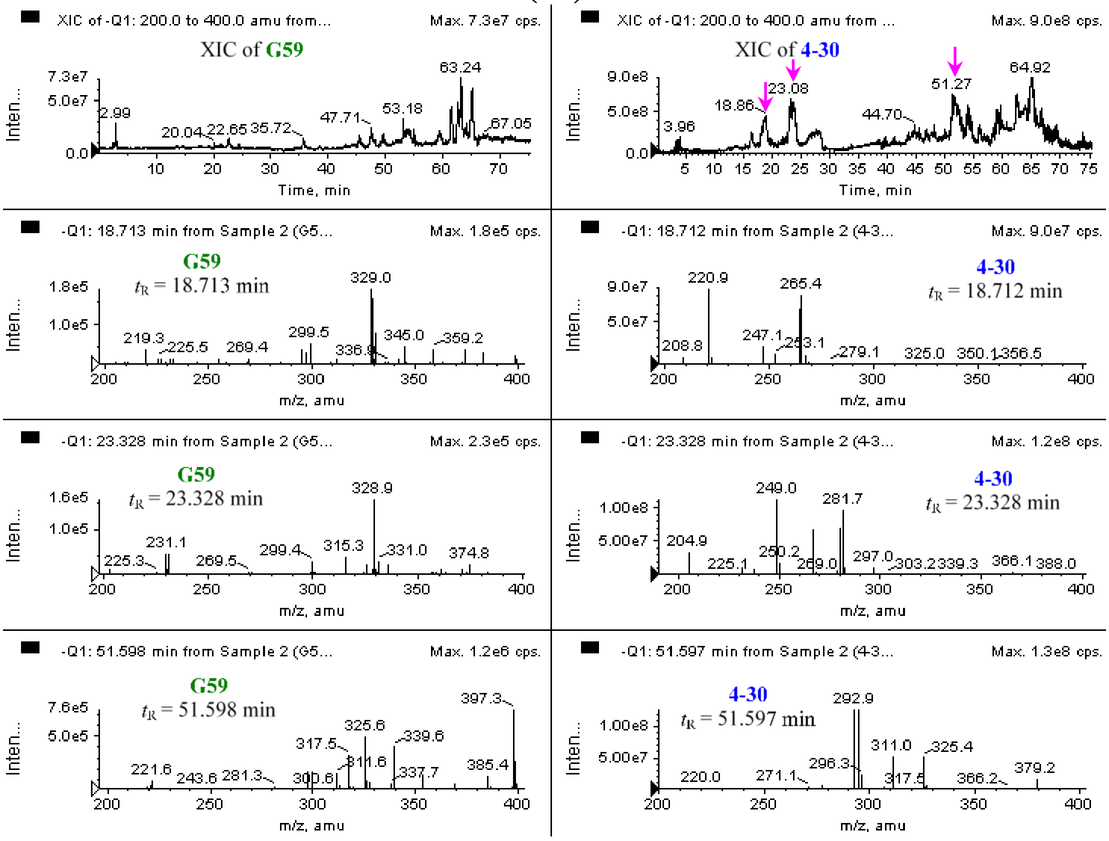


**(F3)**

**Figure S2.** *Cont.*

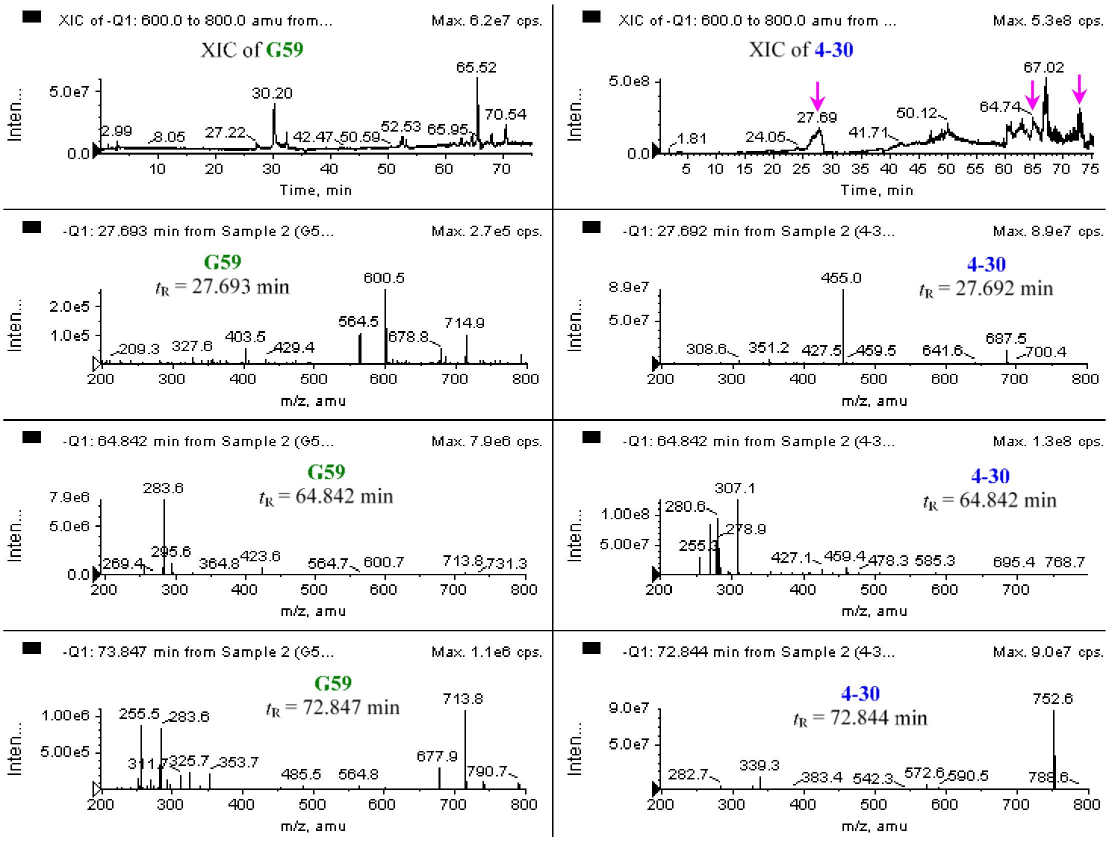


**(F4)**

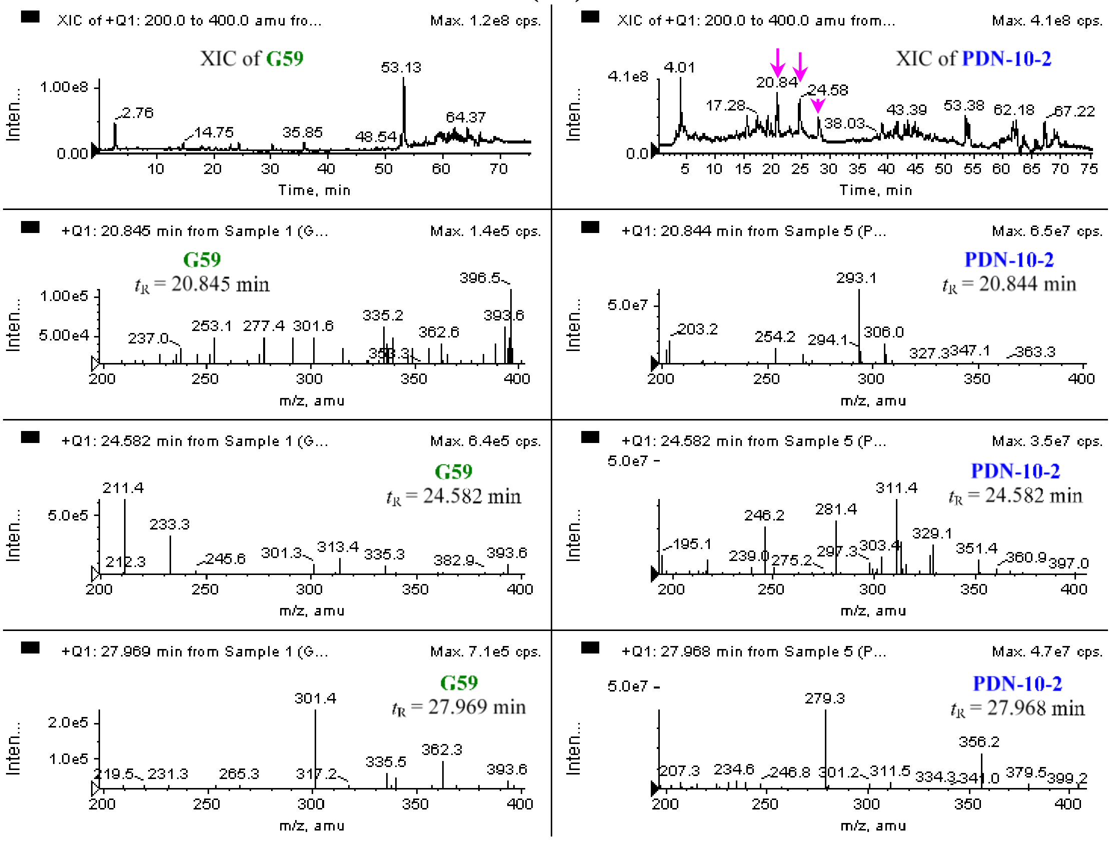


**(G1)**

**Figure S2.** *Cont.*

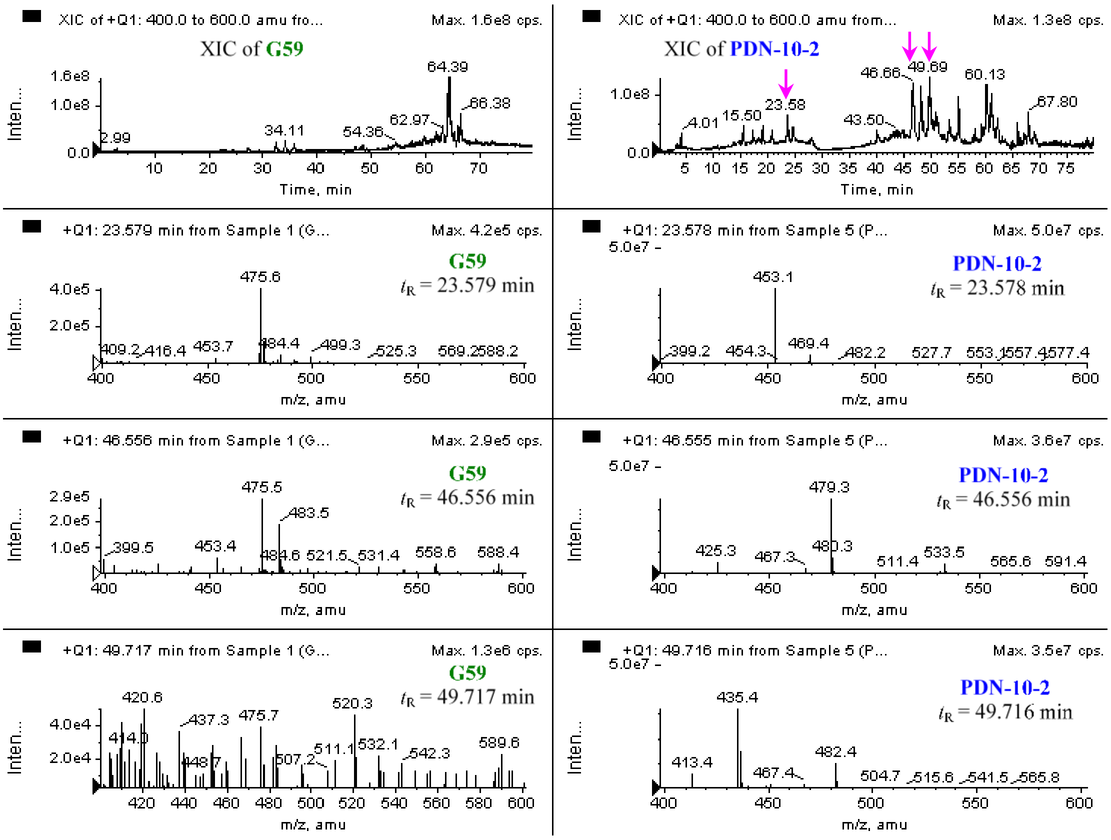


**(G2)**

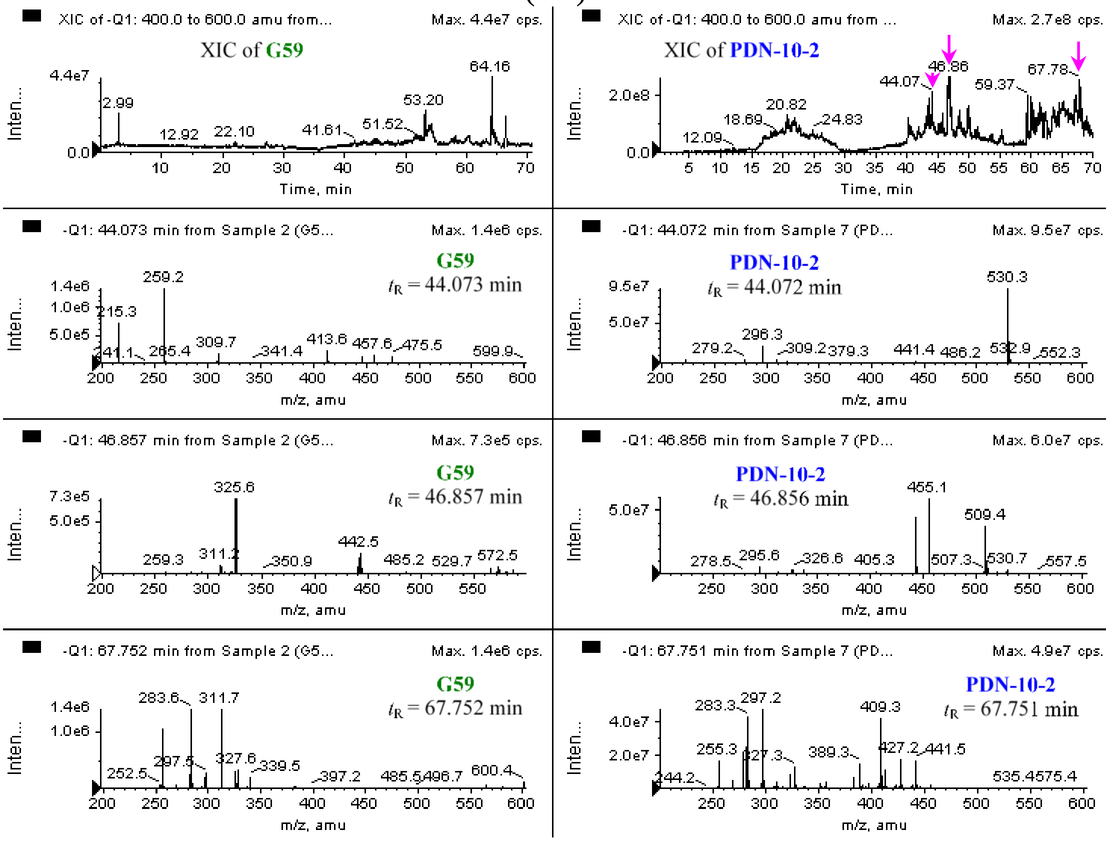


**(G3)**

**Figure S2.** *Cont.*

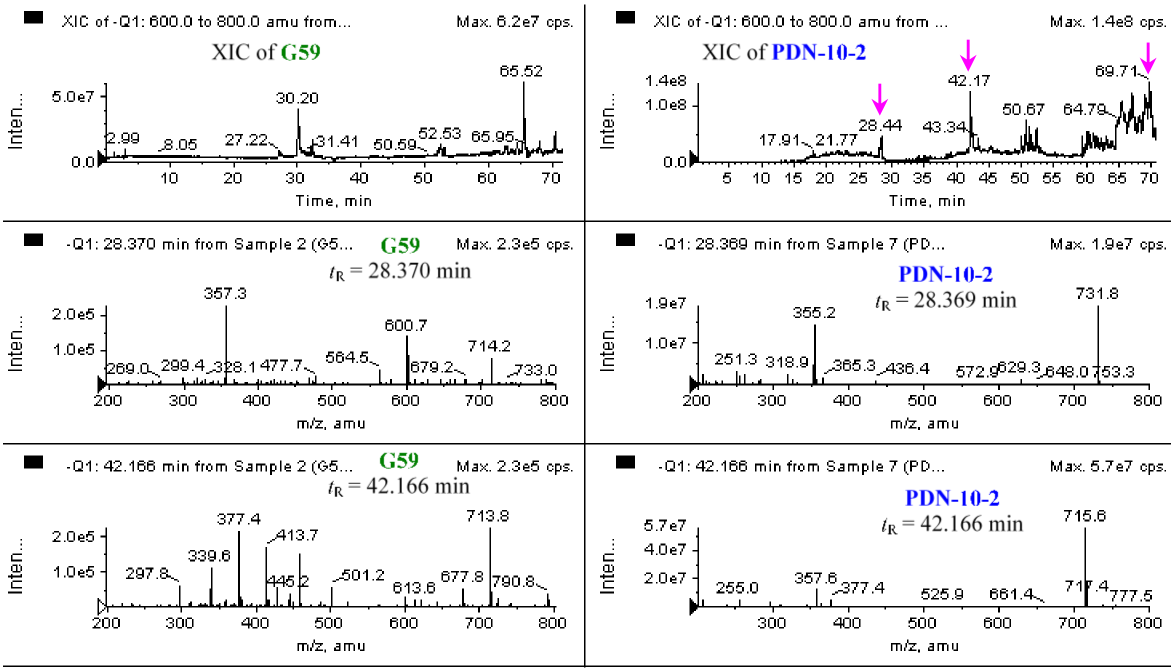


**(G4)**

**Figure S2.** *Cont.*

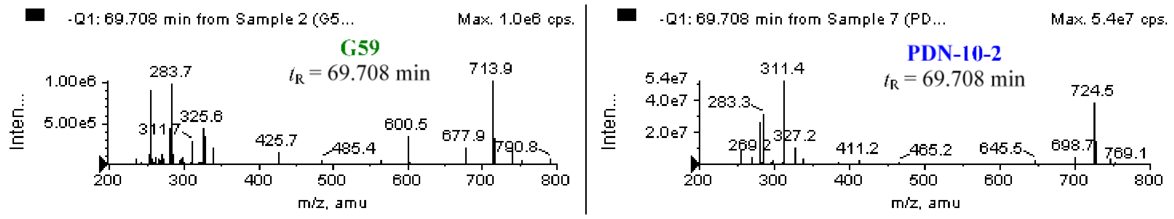


**(G4)**

**Figure S2.** HPLC-ESI-MS analysis of the EtOAc extracts of control G59 strain and its selected mutants. (**A1**) Positive total ion chromatograms; (**A2**) Negative total ion chromatograms; (**B1**) Positive extracted ion chromatograms (XICs) (200–400 *m/z*); (**B2**) Positive extracted ion chromatograms (XICs) (400–600 *m/z*); (**B3**) Positive extracted ion chromatograms (XICs) (600–800 *m/z*); (**B4**) Positive extracted ion chromatograms (XICs) (800–1000 *m/z*); (**C1**) Negative extracted ion chromatograms (XICs) (200–400 *m/z*); (**C2**) Negative extracted ion chromatograms (XICs) (400–600 *m/z*); (**C3**) Negative extracted ion chromatograms (XICs) (600–800 *m/z*); (**C4**) Negative extracted ion chromatograms (XICs) (800–1000 *m/z*); (**D1**) MS spectra extracted from the positive XICs (200–400 *m/z*) of control G59 and mutant 2-50-1; (**D2**) MS spectra extracted from the positive XICs (400–600 *m/z*) of control G59 and mutant 2-50-1; (**D3**) MS spectra extracted from the negative XICs (200–400 *m/z*) of control G59 and mutant 2-50-1; (**D4**) MS spectra extracted from the negative XICs (600–800 *m/z*) of control G59 and mutant 2-50-1; (**E1**) MS spectra extracted from the positive XICs (400–600 *m/z*) of control G59 and mutant 3-f-31; (**E2**) MS spectra extracted from the positive XICs (600–800 *m/z*) of control G59 and mutant 3-f-31; (**E3**) MS spectra extracted from the negative XICs (400–600 *m/z*) of control G59 and mutant 3-f-31; (**E4**) MS spectra extracted from the negative XICs (600–800 *m/z*) of control G59 and mutant 3-f-31; (**F1**) MS spectra extracted from the positive XICs (200–400 *m/z*) of control G59 and mutant 4-30; (**F2**) MS spectra extracted from the positive XICs (400–600 *m/z*) of control G59 and mutant 4-30; (**F3**) MS spectra extracted from the negative XICs (200–400 *m/z*) of control G59 and mutant 4-30; (**F4**) MS spectra extracted from the negative XICs (600–800 *m/z*) of control G59 and mutant 4-30; (**G1**) MS spectra extracted from the positive XICs (200–400 *m/z*) of control G59 and mutant PDN-10-2; (**G2**) MS spectra extracted from the positive XICs (400–600 *m/z*) of control G59 and mutant PDN-10-2; (**G3**) MS spectra extracted from the negative XICs (400–600 *m/z*) of control G59 and mutant PDN-10-2; (**G4**) MS spectra extracted from the negative XICs (600–800 *m/z*) of control G59 and mutant PDN-10-2.

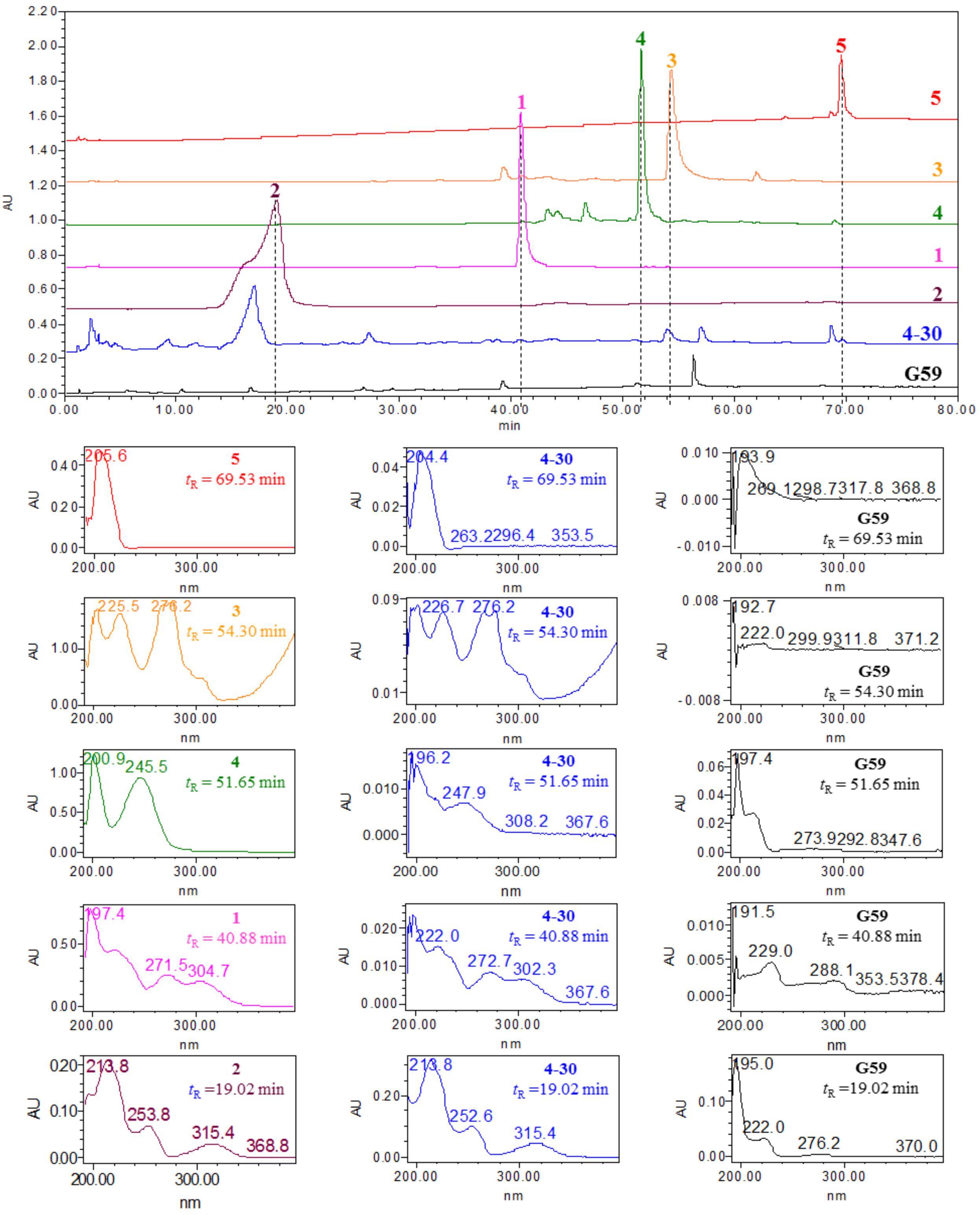

**Figure S3.** HPLC-PDAD-UV analysis of the EtOAc extracts of mutant 4-30 and the control G59 strain for detecting **1**–**5**. HPLC profiles of **1**–**5** and the EtOAc extracts of mutant 4-30 and the control G59 detected at 220 nm. UV spectra of **1**–**5** and the corresponding peaks in the mutant 4-30 and control G59 extracts.

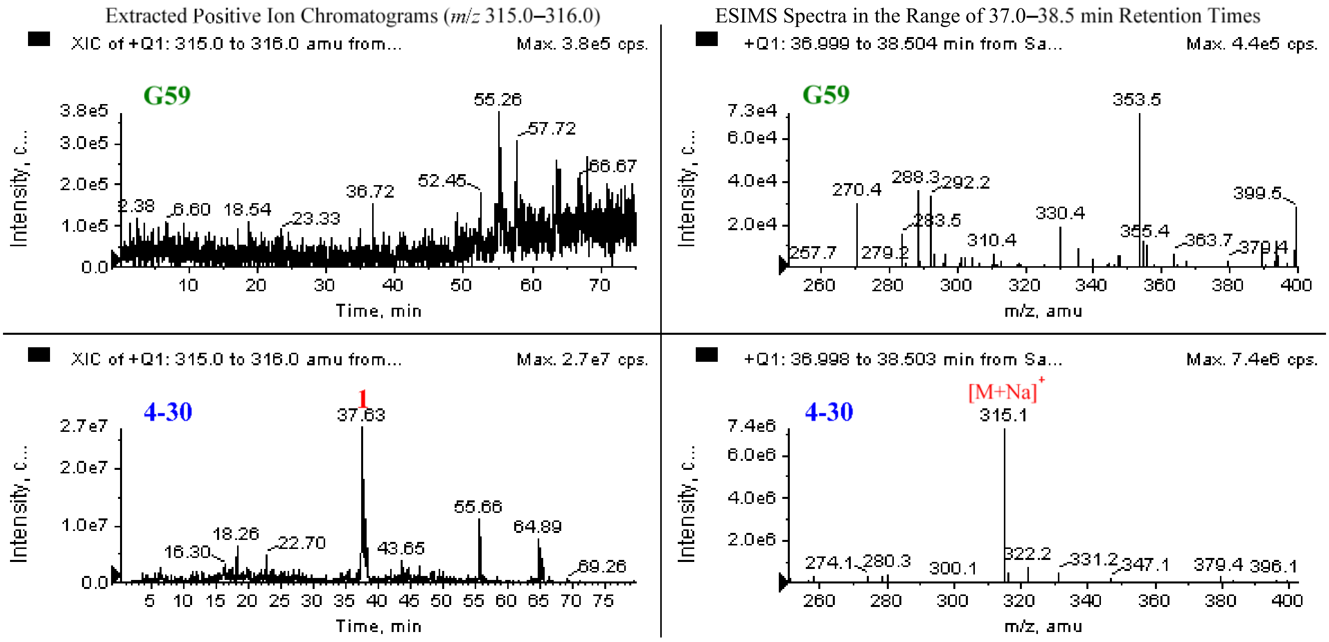


**(A)**

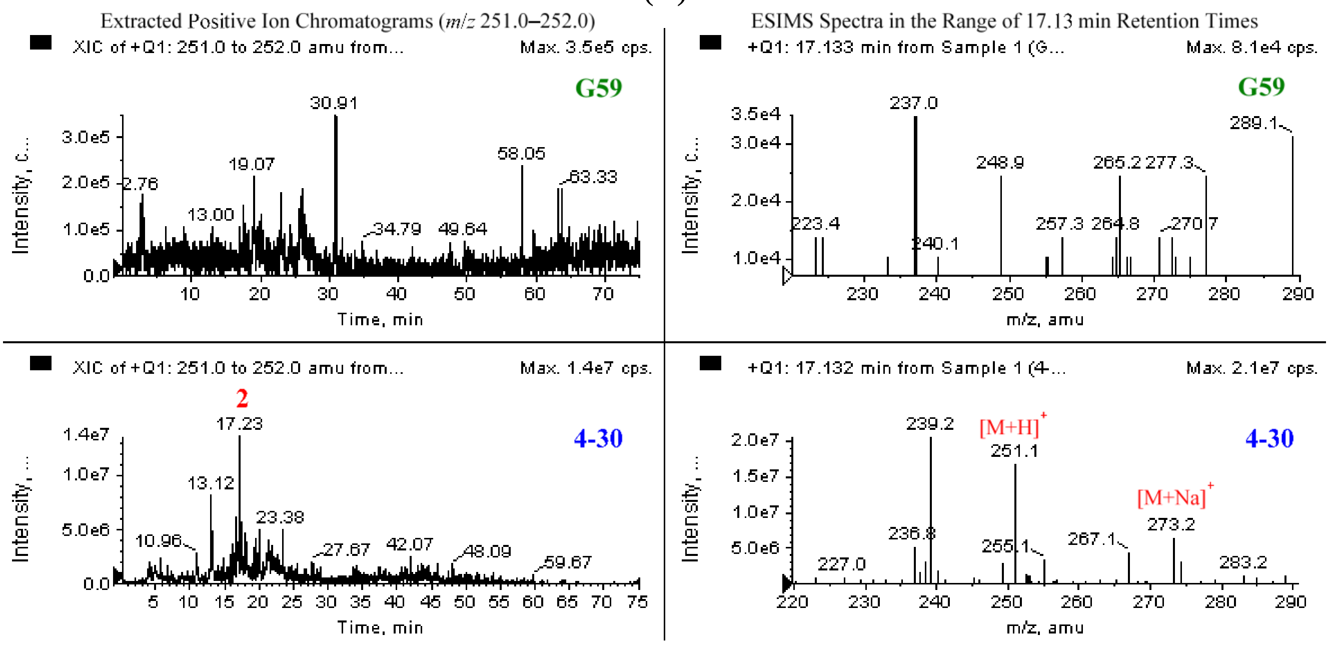


**(B)**

**Figure S4.** *Cont.*

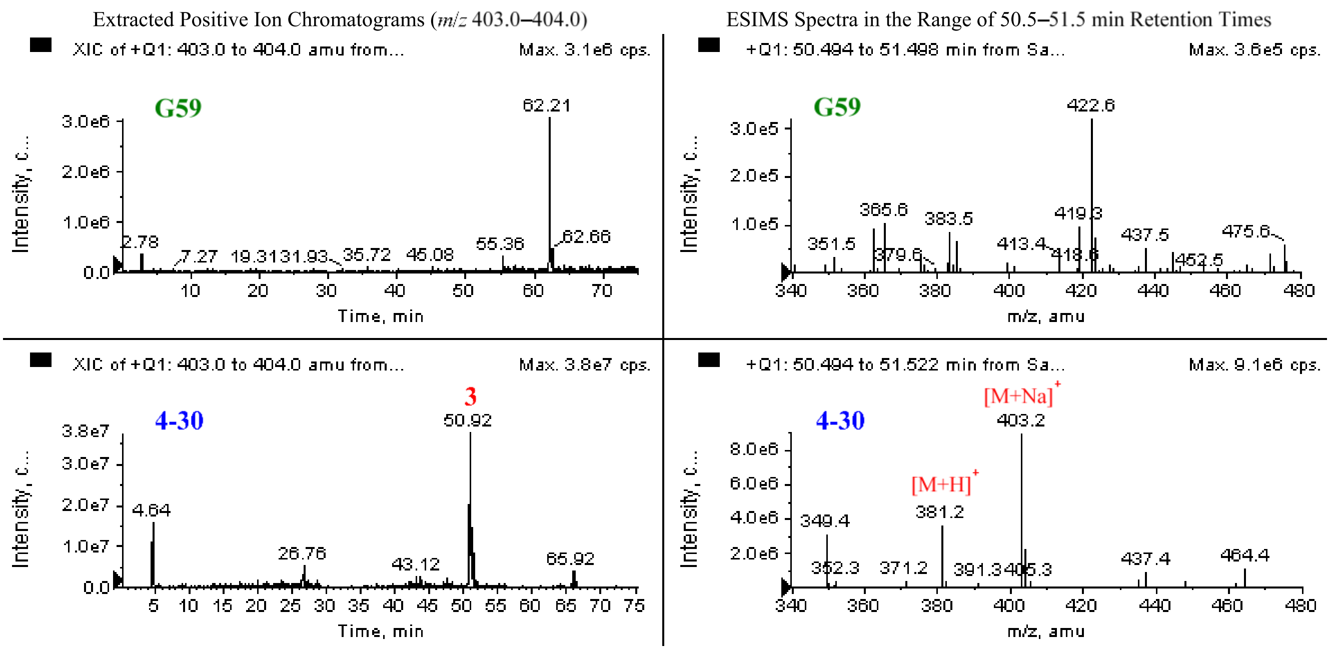


**(C)**

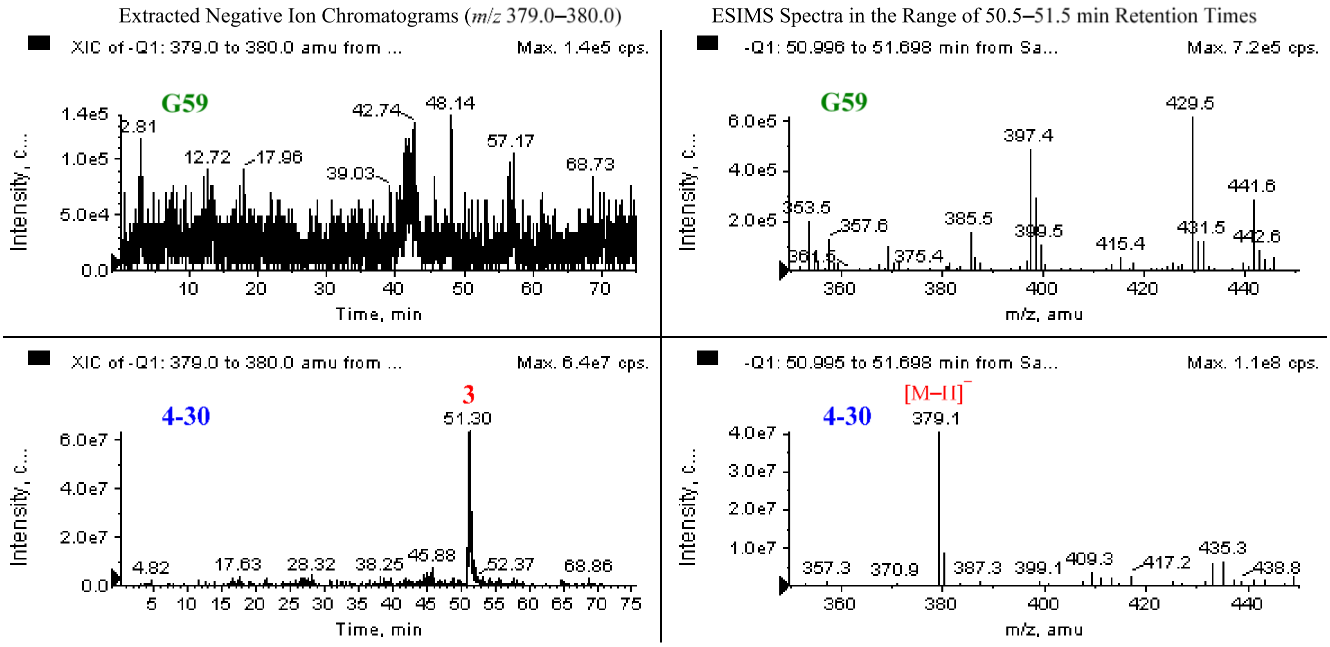


**(D)**

**Figure S4.** *Cont.*

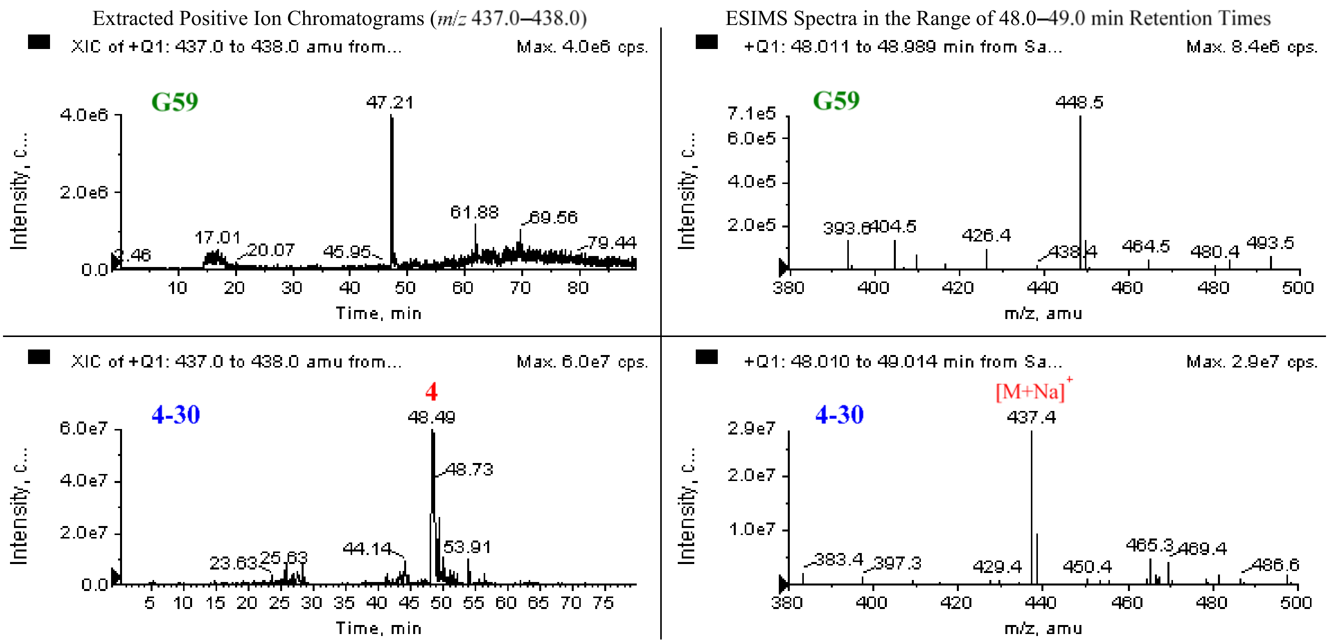


(E)

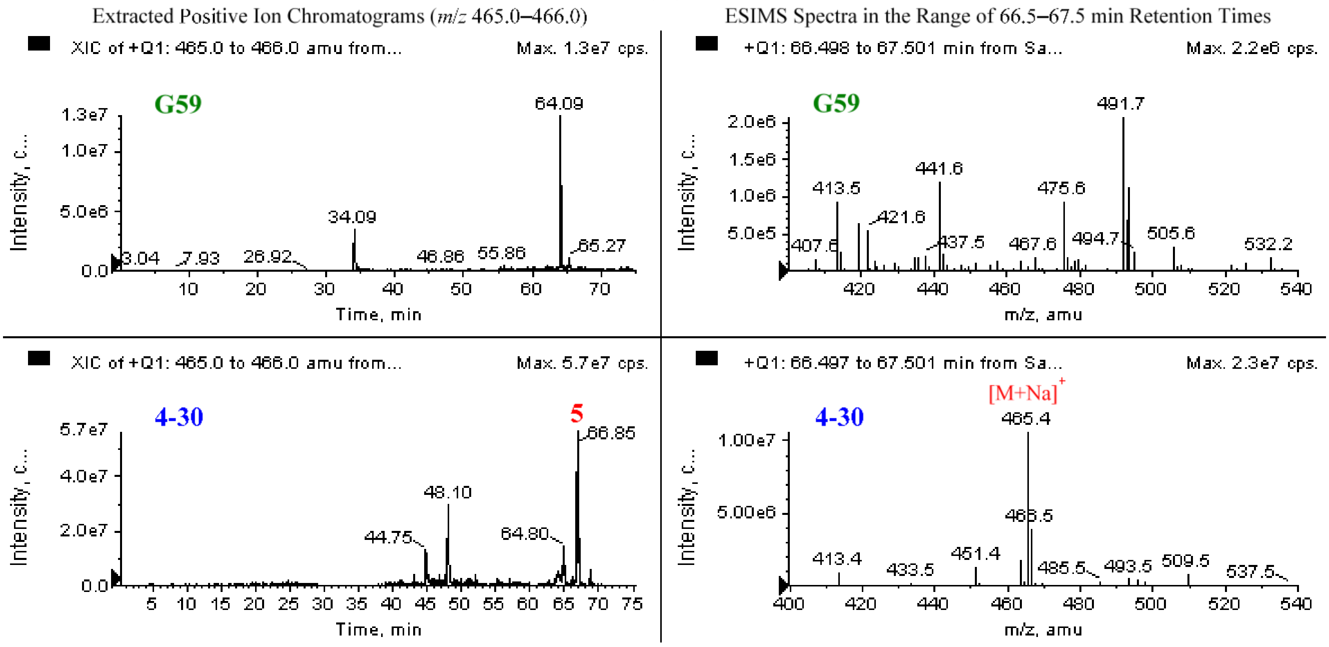


(F)

**Figure S4.** HPLC-ESI-MS analysis of the EtOAc extracts of mutant 4-30 and the control G59 strain for detecting **1**–**5**. (**A**) HPLC-Positive ion ESI-MS analysis (ESIMS *m*/*z*: 315 $[M + Na]^+$ for **1**); (**B**) HPLC-Positive ion ESI-MS analysis (ESIMS *m*/*z*: 251 $[M + H]^+$ for **2**); (**C**) HPLC-Positive ion ESI-MS analysis (ESIMS *m*/*z*: 403 $[M + Na]^+$ for **3**); (**D**) HPLC-Negative ion ESI-MS analysis (ESIMS *m*/*z*: 379 $[M - H]^-$ for **3**); (**E**) HPLC-Positive ion ESI-MS analysis (ESIMS *m*/*z*: 437 $[M + Na]^+$ for **4**); (**F**) HPLC-Positive ion ESI-MS analysis (ESIMS *m*/*z*: 465 $[M + Na]^+$ for **5**).

© 2015 by the authors; licensee MDPI, Basel, Switzerland. This article is an open access article distributed under the terms and conditions of the Creative Commons Attribution license (http://creativecommons.org/licenses/by/4.0/).
